# Inhibition of angiogenesis and regenerative lung growth in *Lep^ob/ob^* mice through adiponectin-VEGF/VEGFR2 signaling

**DOI:** 10.3389/fcvm.2024.1491971

**Published:** 2024-10-16

**Authors:** Tendai Hunyenyiwa, Priscilla Kyi, Mikaela Scheer, Mrudula Joshi, Mario Gasparri, Tadanori Mammoto, Akiko Mammoto

**Affiliations:** ^1^ Department of Pediatrics, Medical College of Wisconsin, Milwaukee, WI, United States; ^2^ Department of Cell Biology, Neurobiology and Anatomy, Medical College of Wisconsin, Milwaukee, WI, United States; ^3^ Department of Thoracic Surgery, Medical College of Wisconsin, Milwaukee, WI, United States; ^4^ Department of Pharmacology and Toxicology, Medical College of Wisconsin, Milwaukee, WI, United States

**Keywords:** angiogenesis, lung regeneration, obesity, adiponectin, VEGF

## Abstract

**Introduction:**

Obesity is associated with impairment of wound healing and tissue regeneration. Angiogenesis, the formation of new blood capillaries, plays a key role in regenerative lung growth after unilateral pneumonectomy (PNX). We have reported that obesity inhibits angiogenesis. The effects of obesity on post-PNX lung vascular and alveolar regeneration remain unclear.

**Methods:**

Unilateral PNX is performed on *Lep*^*ob/ob*^ obese mice to examine vascular and alveolar regeneration.

**Results:**

Regenerative lung growth and expression of vascular endothelial growth factor (VEGF) and its receptor VEGFR2 induced after PNX are inhibited in *Lep*^*ob/ob*^ obese mice. The levels of adiponectin that exhibits pro-angiogenic and vascular protective properties increase after unilateral PNX, while the effects are attenuated in *Lep*^*ob/ob*^ obese mice. Post-PNX regenerative lung growth and increases in the levels of VEGF and VEGFR2 are inhibited in adiponectin knockout mice. Adiponectin stimulates angiogenic activities in human lung endothelial cells (ECs), which is inhibited by decreasing the levels of transcription factor Twist1. Adiponectin agonist, AdipoRon restores post-PNX lung growth and vascular and alveolar regeneration in *Lep*^*ob/ob*^ obese mice.

**Discussion:**

These findings suggest that obesity impairs lung vascular and alveolar regeneration and adiponectin is one of the key factors to improve lung regeneration in obese people.

## Introduction

Obese population is increasing worldwide (1). In the United States, approximately 40% of the adult population is obese (2). Obesity is a risk factor for increased morbidity and mortality through its association with cardiovascular disease, type 2 diabetes (T2D) and certain types of cancer (2–4). Obesity is also a risk factor for respiratory diseases including acute lung injury (5), asthma (6), obstructive sleep apnea (7), and pulmonary hypertension (8). About 65% of the mild-moderate chronic obstructive pulmonary disease (COPD) population is overweight or obese (9).

Although lung transplantation is one of the strategies for end-stage lung diseases including COPD, it is not optimal due to the shortage of donor lungs, lower long-term survival rate, and serious complications (10). In addition, there is an association between obesity and poor outcomes in lung transplantation such as mortality and primary graft dysfunction after transplantation; obesity is currently a relative contraindication to adult lung transplantation (11). It has been reported that compensatory regenerative lung growth is induced after unilateral PNX in humans and other species (12–19); the remaining lung tissues grow to compensate for the initial loss. While it is known that obesity impairs wound healing (20) and attenuates tissue regeneration in other organs including muscle and liver (21, 22), the effects of obesity on post-PNX lung vascular and alveolar regeneration remain unknown. Understanding the mechanism by which obesity inhibits lung's regenerative ability will lead to the development of better therapeutic strategies to restore structures and functions in the end-stage lung diseases in obese patients.

We and other groups have demonstrated that endothelial cells (ECs) and angiogenic signaling are necessary for regenerative vascular and alveolar formation (13, 15, 17–19, 23, 24). In addition to their primary function to deliver oxygen, nutrients and cellular components, capillary ECs form the vascular niche and reciprocally crosstalk with resident lung cells (e.g., epithelial cells, mesenchymal cells, immune cells) to regulate lung homeostasis and regeneration (24). We have reported that angiogenesis is inhibited in obese adipose tissues (25). Insufficient blood vessels in obese animals result in decreases in oxygen tension, collagen synthesis, and immune responses, leading to suppression of wound healing and tissue repair processes (20). Stimulating angiogenesis may restore the regenerative program in the lungs in an obese condition.

One of the adipokines, adiponectin, exhibits pro-angiogenic and vascular protective properties (26, 27). Adiponectin induces angiogenesis through multiple signaling pathways [e.g., AMPK, eNOS, VEGF (3, 26)] and stimulates regeneration of muscle (28) and liver (29). The levels of adiponectin decrease in obese animals, which contributes to obesity-related diseases, such as type2 diabetes and cardiovascular diseases (26, 30). It has been reported that obesity-induced imbalance of adipokines leads to lung EC dysfunction and impairs injury repair (27). Although it is known that adiponectin stimulates proliferation and migration in human bronchial epithelial cells (31), the role of adiponectin in regenerative lung growth remains unclear.

Transcription factor Twist1 controls expression of angiogenic factors, including VEGFR2 and Tie2, and regulates angiogenesis (18, 32–34). We have demonstrated that Twist1 mediates age-dependent inhibition of angiogenesis and lung regeneration (18). Twist1 is also involved in pulmonary fibrosis (34) and endotoxin-induced decreases in vascular integrity (32). The levels of TWIST1 are lower in obese human subcutaneous adipose tissue ECs compared to that in lean adipose tissues, which results in impairment of angiogenesis in obese adipose tissues (25). The role of Twist1 in lung vascular regeneration in the obese lungs has not been studied before.

Here, we found that post-PNX regenerative lung growth is attenuated in *Lep^ob/ob^* obese mice. Knockdown of adiponectin decreases expression of Twist1, VEGF and VEGFR2, and inhibits post-PNX regenerative lung growth and vascular formation in the mouse lung, while adiponectin agonist, AdipoRon restores post-PNX lung growth in obese mice. Adiponectin could be one of the efficient targets for lung vascular and alveolar regeneration in obese patients.

## Materials and methods

### Materials

AdipoRon hydrochloride was from Tocris (Minneapolis, MN). Adiponectin was from R&D (Minneapolis, MN). Anti-CD31 antibody (553370) was from BD Pharmingen (San Jose, CA). Anti-SPB (ab40876), -AQP5 (ab78486), -TWIST1 (ab50887) and -ERG (ab92513) antibodies were from Abcam (Cambridge, MA). Anti-VEGF164 (AF-493-NA) and -RAGE (MAB1179) antibodies were from R&D. Anti-VEGFR2 antibody (2479) was from Cell Signaling (Danvers, MA). Anti-adiponectin antibody (MA1-054) was from Thermo Fisher Scientific (Waltham, MA). Anti-actin antibody (A5441) was from Sigma (St. Louis, MO).

### Molecular biological and biochemical methods

Quantitative reverse transcription (qRT)-PCR was performed with the iScript reverse transcription and iTaq SYBR Green qPCR kit (BioRad, Hercules, CA) using the BioRad real time PCR system. Cyclophilin and beta-2-microglobulin controlled for overall cDNA content. The primers for mouse Vegf, Vegfr2, Twist1 and cyclophilin and human TWIST1, VEGFR2, and beta-2-microglobulin were previously described (18, 35). The primers for mouse adiponectin forward; 5′-TGTTCCTCTTAATCCTGCCCA-3′ and reverse; 5′-CCAACCTGCACAAGTTCCCTT-3′, Adipor1 forward; 5’-AGACAACGACTACCTGCTACA-3’ and reverse; 5’-GTGGATGCGGAAGATGCTCT-3’; Adipor2 forward; 5’-GCCAAACACCGATTGGGGT-3’ and reverse; 5’-GGCTCCAAATCTCCTTGGTAGTT-3’. The protein levels of mouse VEGF and adiponectin were measured using ELISA (R&D systems). Immunoblotting was performed as we previously reported (19, 36). Gene knockdown was performed using the RNA interference technique as we previously reported (18, 35). In brief, we used siLentfect transfection reagent (BioRad) with siRNA (10 nM) following manufacturer instruction. The siRNA for human TWIST1 was previously described (18). As a control, siRNA duplex with an irrelevant sequence (QIAGEN, Hilden, Germany) was used.

### Mouse and human lung EC isolation

C57BL6 (stock# 664), adiponectin knockout (*B6;129-Adipoq^tm1Chan^/J*; *Adipoq^−/−^*, stock# 8195) and control B6129SF2/J (stock# 101045) mice were obtained from the Jackson Laboratory (Bar Harbor, ME). Adiponectin mRNA expression decreased by 82% in lungs isolated from *Adipoq^−/−^* mice compared with those from control B6129SF2/J mice (Figure 3A). Heterozygote *B6.V-Lepob/J* mice (stock# 632, *Lep^ob/+^*) were obtained from the Jackson Laboratory and bred to obtain homozygote (*Lep^ob/ob^*). *Lep^ob/+^* mice were maintained with standard diet (LabDiet 5LOD, 4.5% fat). For the PNX experiments, *Lep^ob/+^* and *Lep^ob/ob^* mice were fed with LabDiet 5K20 (10% fat) for 8 weeks, starting at 4 weeks of age. Human lung tissues were obtained as discarded surgical specimens from patients [Medical College of Wisconsin (MCW) tissue bank; Table 1]. De-identified patient demographic data were collected using the Generic Clinical Research Database at MCW. All protocols are approved by the Institutional Review Board (IRB) of MCW and Froedtert Hospital and ECs isolated from de-identified human lungs are determined as non-human subjects (PRO00047689).

**Table 1:** Sample demographics.

ID	Age	BMI	Sex	Race
Lean -1	60	26	Male	N/A
Lean -2	75	23	Female	White
Lean -3	68	21	Male	White
Lean -4	34	26	Female	White
Lean -5	65	27	Female	Black
Obese -1	67	37	Female	White
Obese -2	60	31	Female	White
Obese -3	42	31	Male	N/A
Obese -4	57	33	Male	White
Obese -5	57	39	Male	White

ECs from mouse lungs and human lung ECs were isolated using anti-CD31 conjugated magnetic beads as previously described (17, 19, 25, 36). Briefly, lung tissue was cut into small pieces using small scissors and treated with collagenase A (1 mg/ml) for 30 min at 37°C. The tissue suspension was filtered through a 40 mm cell strainer (Falcon) to remove the undigested cell clumps and separate single cells. Cells were centrifuged (1,000 rpm, 5 min) at room temperature (RT) and the pellet was resuspended into 0.5 ml RBC Lysis Buffer (sigma, 1 min, RT). The lysis reaction was stopped by adding 10 ml 10% FBS/DMEM, and centrifuged (1,000 rpm, 5 min, RT). For mouse lung EC isolation, the pellet was resuspended into 0.5 ml 4% FBS/PBS with APC anti-mouse CD31 antibody (Biolegend, San Diego, CA, 1/100), incubated (20 min, on ice) and washed three times with 4% FBS/PBS. Cells were centrifuged (1,000 rpm, 5 min, RT) and resuspended into 0.1 ml 4% FBS/PBS with anti-APC conjugated microbeads (Miltenyl Biotec, Bergisch Gladbach, Germany), incubated (10 min, on ice) and washed three times with 4% FBS/PBS. The cells were then resuspended in 0.5 ml 4% FBS/PBS and CD31-positive mouse ECs were magnetically separated using MACS column (Miltenyl Biotec) according to the manufacturer's instruction. To increase the purity of the magnetically separated fraction, the eluted fraction was enriched over a second new MACS column. For human lung EC isolation, the cell pellet was resuspended into 1 ml 4% FBS/PBS with CD31-conjugated Dynabeads (Invitrogen/Thermo Fisher), incubated (30 min, 4°C), washed three times with 4% FBS/PBS, and magnetically separated using a magnetic stand. FACS analysis confirmed that more than 89% of isolated ECs cells are CD31^+^ (Supplementary Figure S1A) (17, 19, 25, 36).

### Cell biological analysis

Human lung ECs were seeded (1 × 10^5^ cells/35 mm dish) and DNA synthesis was measured using the Click-iT EdU Cell Proliferation Kit (ThermoFisher). Cells were imaged using a Nikon A1R confocal laser scanning microscope and quantification was performed using ImageJ software (NIH) (18, 25, 36). EC migration was analyzed by seeding human lung ECs (1 × 10^5^ cells/100 μl) on a trans-well chamber (Corning Costar) coated with 0.5% gelatin. ECs migrating to 5% FBS for 16 h were stained with Wright Giemsa solution (Fisher Scientific) and counted (18, 25, 36).

### Unilateral PNX

The *in vivo* animal study was carried out in strict accordance with the recommendations in the Guide for the Care and Use of Laboratory Animals of the National Institutes of Health. The protocols were reviewed and approved by the Institutional Animal Care and Use Committee of MCW (AUA 5598). Unilateral PNX was performed as described (17–19). Briefly, mice (C57BL6, *Lep^ob/+^*, *Lep^ob/ob^*, *Adipoq^−/−^,* or B6129SF2/J, 12–15 week old) were anesthetized with Ketamine (100 mg/kg)/Xylazine (10 mg/kg, intraperitoneal injection), intubated and mechanically ventilated using a rodent ventilator (MiniVent, Harvard Apparatus, Holliston, MA). After ensuring adequate anesthesia, thoracotomy was performed, and the left lung was lifted through the incision and a 5-0 silk suture was passed around the hilum and tied. The hilum was then transected distal to the tie. The remaining portions of the hilum and tie were returned to the thoracic cavity. Sham-operated mice underwent thoracotomy without PNX.

### Bulk RNA sequencing and analysis

RNA was collected from ECs isolated from C57BL6 mouse lungs 7 days after PNX and sham-operated mouse lungs using the RNeasy mini kit (Qiagen). The quantity and quality of RNA isolated from mouse lung ECs (*n* = 3 per group, each *n* was pooled from 2 mice) were measured by Agilent 2200 TapeStation, and all samples have an RNA integrity number >9.3. Total RNA samples were submitted to the Institute for Systems Biology Molecular and Cell Core (Seattle, WA) for RNA sequencing. Library preparation was employed using the Illumina TruSeq Stranded mRNA kit. Sequencing was performed using the Illumina NextSeq500. Paired-end sequencing was performed on a high output 150 cycle kit v2.5. The RNA sequencing reads were aligned to the mouse genome (mm10 reference genome) and read counting and differential gene expression analysis were performed with Basepair Tech using the Deseq2 pipeline. 903 significantly differentially expressed genes defined as having a |log2 fold change| >1, and a *p*-adjusted value with the FDR cutoff of 0.01 calculated by the Benjamini-Hochberg adjustment and filtered to <0.01 were defined (Supplementary Table S1). Gene ontology (GO) analysis of significant targets was done via The Database for Annotation, Visualization and Integrated Discovery (DAVID) v 6.8 using the Functional Annotation Chart tool. Charts were filtered by Biological Processes Gene Ontology (BP GO) Terms and sorted by *p*-value (Supplementary Table S2). The 903 significantly differentially expressed genes generated 262 BP GO Term categories (Supplementary Table S2), which are further categorized into two different GO Term charts; 117 GO Term categories related to angiogenesis (Supplementary Table S3) and 141 GO Term related to metabolic process detected as appearing on a master list comprised of Gene Card (Supplementary Table S5). These GO Terms categories were color-coded into groups encompassing adhesion/migration, cell cycle/apoptosis, inflammatory/immune response, and cell signaling (Supplementary Tables S4, S6).

Network generation was performed on all genes from the top 50 GO Term categories by *p*-value related to angiogenic factors or metabolic process with Ingenuity Pathway Analysis (IPA) software (QIAGEN). The network of angiogenic genes was constructed by starting with the shortest connections between those connected to Twist1 and all others, and adding the shortest connections between all genes connected to Twist1 and the remaining unconnected genes. Genes were eliminated if they were connected to less than 3 other genes. The network of metabolic genes was constructed and interaction with adiponectin was analyzed as described for Twist1. The resulting IPA networks from the angiogenic factors and metabolic processes were combined to create a network illustrating the overlapping genes between the angiogenic factor and metabolic processes categories. RNAseq results are available in NCBI Geo (GSE179227).

### Statistics

All phenotypic analysis was performed by masked observers unaware of the identity of experimental groups. Error bars (SEM) and *p* values were determined from the results of three or more independent experiments. Student's *t*-test was used for statistical significance for two groups. For more than two groups, one-way ANOVA with a post-hoc analysis using the Bonferroni test was conducted.

## Results

### Post-PNX lung growth and vascular formation are inhibited in obese mice

**Figure 1:**
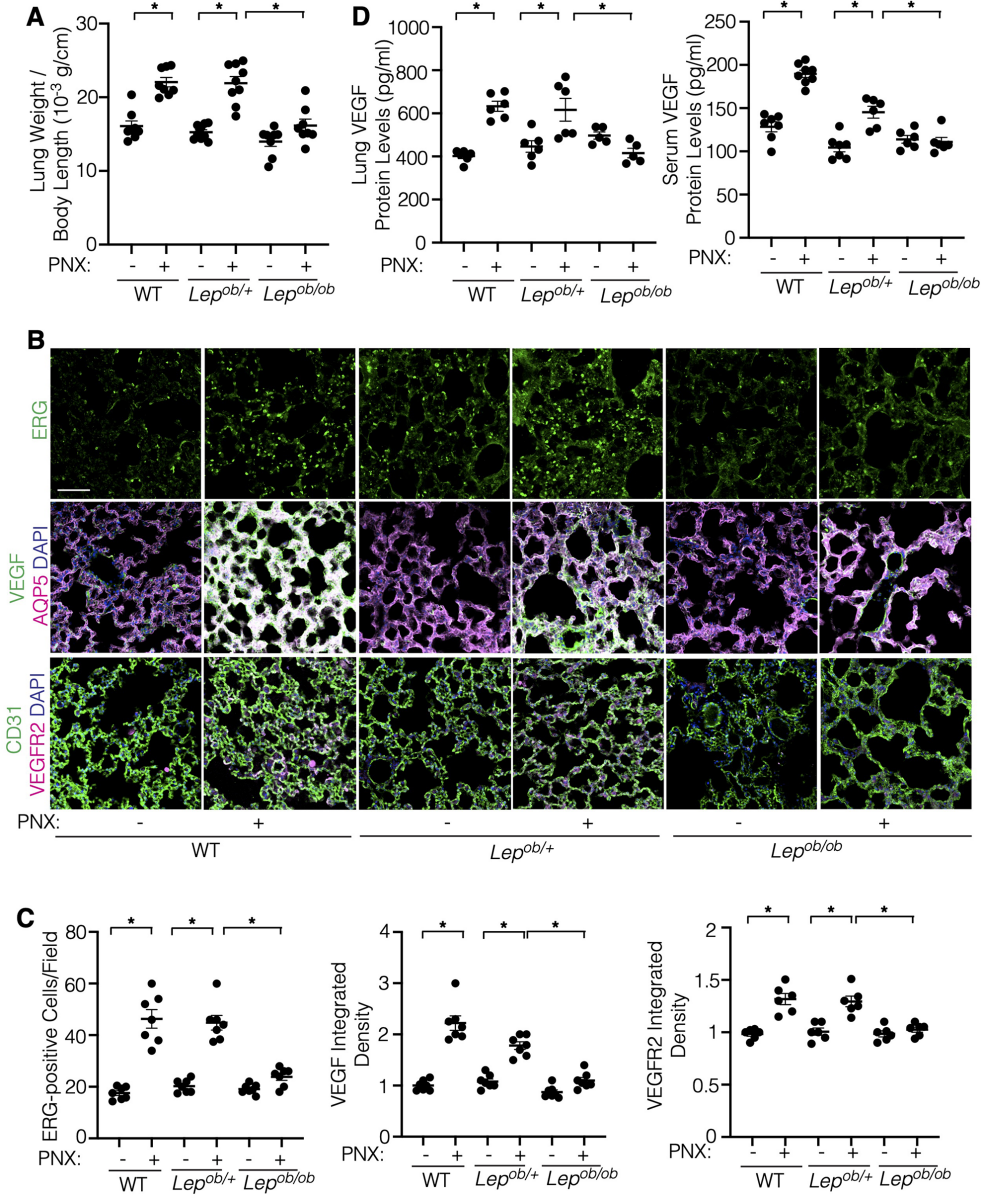
Post-PNX regenerative lung growth is inhibited in the *Lep^ob/ob^* mouse lungs. **(A)** Graph showing the ratio of the weight of right lung lobe to mouse body length in C57BL6 (WT), *Lep^ob/+^* or *Lep^ob/ob^* mice 7 days after PNX (*n* = 8–9, mean ± s.e.m., *p < 0.05). **(B)** Immunofluorescence (IF) micrographs showing ERG-positive ECs (*top*), VEGF expression, AQP5-positive alveolar epithelial cells and DAPI (*middle*) and CD31-positive blood vessels and VEGFR2 expression and DAPI (*bottom*) in the C57BL6 (WT), *Lep^ob/+^* or *Lep^ob/ob^* mouse lungs 7 days after PNX. Scale bar, 50 μm. **(C)** Graphs showing the quantification of ERG-positive ECs (*left*), VEGF (*middle*) and VEGFR2 (*right*) expression in the C57BL6 (WT), *Lep^ob/+^* or *Lep^ob/ob^* mouse lungs 7 days after PNX (*n* = 6–7, mean ± s.e.m., *p < 0.05). **(D)** Graphs showing the VEGF protein levels in the C57BL6 (WT), *Lep^ob/+^* or *Lep^ob/ob^* mouse lungs (*left*) and serum (*right*) 7 days after PNX (*n* = 5–8, mean ± s.e.m., *p < 0.05).

Angiogenic signaling is necessary for lung vascular and alveolar regeneration after unilateral PNX (13, 15, 17–19, 23). Angiogenesis is inhibited in obese adipose tissues (25). The effects of obesity on regenerative ability of the lungs remain unknown. When we performed unilateral PNX on C57BL6 mice under normal chaw (4.5% fat, 5LOD) or on *Lep^ob/+^* or *Lep^ob/ob^* mice fed with 10% fat diet (5K20), there was a significant increase in the ratio of the weight of right lung lobe to mouse body length 7 days after left unilateral PNX in C57BL6 or *Lep^ob/+^* mice as consistent with previous reports (13, 15, 17–19); the ratio of lung weight to mouse body length was 14.2 × 10^−3^ (g/cm) in the sham-operated control mice, while the ratio increased by 1.5-fold in the lungs 7 days after PNX (Figure 1A). Although the lung weight was not significantly different among C57BL6, *Lep^ob/^*^+^ and *Lep^ob/ob^* sham-operated mice, the increases in the lung weight after PNX were significantly reduced in *Lep^ob/ob^* mice 7 days after PNX compared to those in C57BL6 or *Lep^ob/+^* mice (Figure 1A). We also analyzed the effects of obesity on blood vessel density in the lung using EC marker, ETS-related gene (ERG) staining; ERG-positive EC density was 2.2- or 2.1- times higher in the C57BL6 or *Lep^ob/+^* mouse lungs after PNX compared to those in the sham-operated control mouse lungs, while these effects were suppressed in *Lep^ob/ob^* mice after PNX (Figures 1B,C). These findings suggest that post-PNX lung growth and vascular regeneration are inhibited in obese mice.

A major angiogenic factor, VEGF is necessary for post-PNX lung growth and vascular regeneration (13). The protein levels of VEGF in the mouse lungs and the serum in C57BL6 mice and *Lep^ob/+^* mice increased 7 days after PNX, while these effects were attenuated in *Lep^ob/ob^* mice after PNX (Figure 1D). Immunohistochemical (IHC) analysis confirmed that VEGF expression in AQP5-positive alveolar type1 (AT1) cells (37) increased in the post-PNX C57BL6 and *Lep^ob/+^* mouse lungs. In contrast, the post-PNX increases in the VEGF expression were suppressed in *Lep^ob/ob^* mice after PNX (Figures 1B,C). Similarly, post-PNX increases in the VEGFR2 expression were inhibited in *Lep^ob/ob^* mice (Figures 1B,C; Supplementary Figure S1B). Among three VEGF isoforms (VEGF120, 164, and 188), the mRNA levels of Vegf164 increased after PNX in control *Lep^ob/+^* mouse lungs, but not in the *Lep^ob/ob^* mouse lungs (Supplementary Figure S1C). The levels of Vegf120 and 188 did not significantly change in the post-PNX mouse lungs (not shown). We have reported that angiopoietin (Ang)-Tie2 signaling is stimulated after PNX to control regenerative lung growth (17, 19, 38). Thus, we also examined the expression of Ang-Tie2 in the post-PNX mouse lungs. Ang2 and Tie2 expression significantly increased in the control *Lep^ob/+^* mouse lungs after PNX; in contrast, the effects were inhibited in *Lep^ob/ob^* mouse lungs (Supplementary Figure S1D).

### Suppression of adiponectin mediates obesity-dependent decline in angiogenesis and post-PNX lung growth

**Figure 2:**
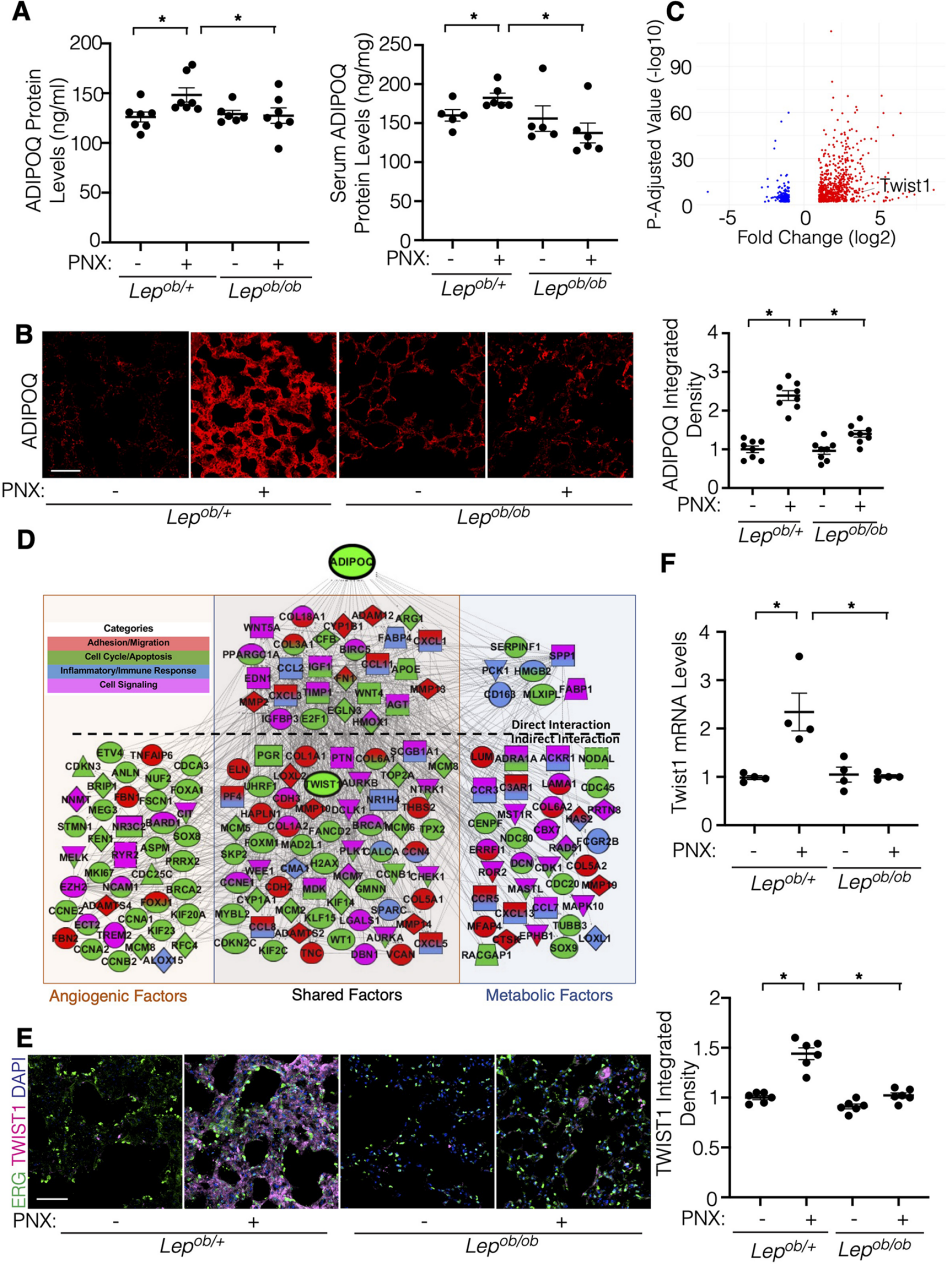
Post-PNX increases in the adiponectin (ADIPOQ) expression are suppressed in *Lep^ob/ob^* mice. **(A)** Graphs showing the quantification of ADIPOQ protein expression in the *Lep^ob/+^* or *Lep^ob/ob^* mouse lungs (*left*) and serum (*right*) 7 days after PNX (*n* = 5–7, mean ± s.e.m., *p < 0.05). **(B)** IF micrographs showing ADIPOQ expression in the *Lep^ob/+^*or *Lep^ob/ob^* mouse lungs 7 days after PNX. Scale bar, 50 μm. Graph showing the ADIPOQ expression (*n* = 8, mean ± s.e.m., *p < 0.05). **(C)** Volcano plots of 903 significantly differentially expressed genes in ECs isolated from C57BL6 mouse lungs 7 days after PNX compared to those in sham-operated mouse lungs (GSE179227). Twist1 is a significantly differentially expressed gene. **(D)** Gene expression profiles and networks in ECs isolated from post-PNX (day 7) vs. sham-operated mouse lungs. **(E)** IF micrographs showing ERG-positive ECs, TWIST1 expression and DAPI in the *Lep^ob/+^* or *Lep^ob/ob^* mouse lungs 7 days after PNX. Scale bar, 50 μm. Graph showing the TWIST1 expression in the *Lep^ob/+^* or *Lep^ob/ob^* mouse lungs 7 days after PNX (*n* = 6, mean ± s.e.m., *p < 0.05). **(F)** Graph showing the Twist1 mRNA levels in the *Lep^ob/+^* or *Lep^ob/ob^* mouse lung ECs 7 days after PNX (*n* = 4, mean ± s.e.m., *p < 0.05).

Adiponectin induces angiogenesis (26, 27) and stimulates tissue regeneration [muscle (28), liver (29)]. The levels of adiponectin decrease in obese animals (26). The protein levels of adiponectin in lungs and serum increased 7 days after PNX in *Lep^ob/+^* mice; in contrast, the effects were suppressed in post-PNX *Lep^ob/ob^* mice (Figure 2A). IHC analysis confirmed that increases in the levels of adiponectin in the post-PNX *Lep^ob/+^* mouse lungs were attenuated in *Lep^ob/ob^* mouse lungs (Figure 2B). Expression of the adiponectin receptor, Adipor1 and Adipor2 did not change after PNX as well as *Lep^ob/+^* vs. *Lep^ob/ob^* mouse lungs (Supplementary Figure S1E). These results suggest that obesity suppresses the levels of adiponectin in the post-PNX mouse lungs.

Transcription factor Twist1 controls expression of angiogenic factors to regulate angiogenesis (18, 32–34). We have reported that the levels of TWIST1 are lower in obese human subcutaneous adipose tissue ECs compared to that in lean adipose tissues, which inhibits angiogenesis (25). Knockdown of endothelial Twist1 inhibited post-PNX lung growth (18). In the bulk RNAseq analysis (GSE179227, Supplementary Table S1), the levels of Twist1 significantly increased in ECs isolated from the post-PNX mouse lungs (7 days, Figure 2C). IPA network analysis demonstrated that genes listed in the top 50 BP GO terms of each category (129 genes in angiogenic and 131 genes in metabolic processes) interacted with Twist1, and 88 genes were present in both categories relating to angiogenic and metabolic processes; 27 genes interacted directly (e.g., ADAM12, CXCL1, MMP2, MMP13, COL3A, WNT5A) and 61 genes interacted indirectly (e.g., LOXL2, COL6A, FOXM1, WT1, THBS2, CXCL5) with adiponectin (Figure 2D).

Consistent with the RNAseq data, TWIST1 expression increased in the post-PNX *Lep^ob/+^* mouse lungs, but not in the *Lep^ob/ob^* mouse lungs (Figure 2E). Twist1 mRNA expression was also 2.2-times higher in ECs isolated from *Lep^ob/+^* mouse lungs 7 days after PNX, while the levels of Twist1 did not significantly change in post-PNX *Lep^ob/ob^* mouse lung ECs (Figure 2F), suggesting that obesity inhibits post-PNX induction of Twist1 expression.

### Adiponectin is required for vascular and alveolar regeneration in the mouse lungs

**Figure 3:**
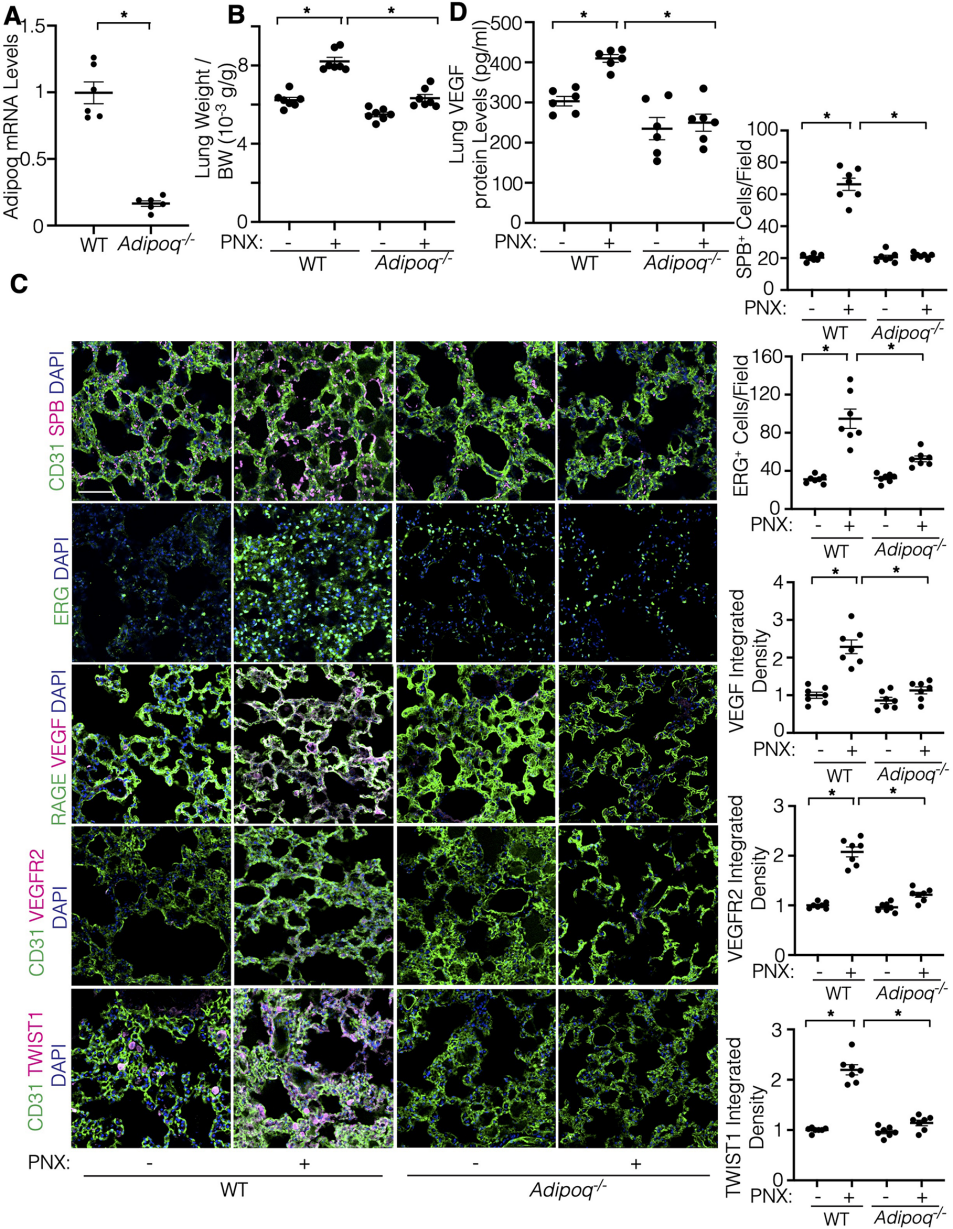
Post-PNX regenerative lung growth is inhibited in the *Adipoq^−/−^* mouse lungs. **(A)** Graph showing the adiponectin mRNA levels in the *Adipoq^−/−^* or control WT mouse lungs (*n* = 6, mean ± s.e.m., *p < 0.05). **(B)** Graph showing the ratio of the weight of right lung lobe to body weight (BW) in *Adipoq^−/−^* or control WT mice 7 days after PNX (*n* = 7, mean ± s.e.m., *p < 0.05). **(C)** IF micrographs showing CD31-positive blood vessels, SPB-positive AT2 cells and DAPI (*top*), ERG-positive ECs and DAPI (*2nd*), RAGE-positive alveolar epithelial cells, VEGF expression and DAPI (*3rd*), CD31-positive blood vessels, VEGFR2 expression and DAPI (*4th*), and CD31-positive blood vessels, TWIST1 expression and DAPI (*bottom*) in the *Adipoq^−/−^* or control WT mouse lungs 7 days after PNX. Scale bar, 50 μm. Graphs showing the quantification of SPB-positive AT2 cells (*top*), ERG-positive ECs (*2nd*), VEGF expression (*3rd*), VEGFR2 expression (*4th*), and TWIST1 expression (*bottom*) in the *Adipoq^−/−^* or WT mouse lungs 7 days after PNX (*n* = 7, mean ± s.e.m., *p < 0.05). **(D)** Graph showing the VEGF protein levels in the *Adipoq^−/−^* or control WT mouse lungs 7 days after PNX (*n* = 6, mean ± s.e.m., *p < 0.05).

To analyze the effects of adiponectin on regenerative lung growth, we performed unilateral PNX on *Adipoq^−/−^* mice, in which the mRNA levels of adiponectin in the mouse lungs were lower by 82% compared to those in the background matched wild type (WT) B6129SF2/J mouse lungs (Figure 3A). While the weight of the remaining right lung lobe increased by 1.3-times in control WT B6129SF2/J mice 7 days after PNX, the increase in the post-PNX lung weight was suppressed in the *Adipoq^−/−^* mouse lungs (Figure 3B). Post-PNX increases in the number of surfactant protein B (SPB)-positive alveolar type-II (AT2) cells and ERG-positive ECs in the WT mouse lungs were suppressed in the *Adipoq^−/−^* mouse lungs (Figure 3C). Increases in the levels of VEGF, VEGFR2, and TWIST1 after PNX were also attenuated in the *Adipoq^−/−^* mouse lungs (Figures 3C,D; Supplementary Figure S1F).

**Figure 4:**
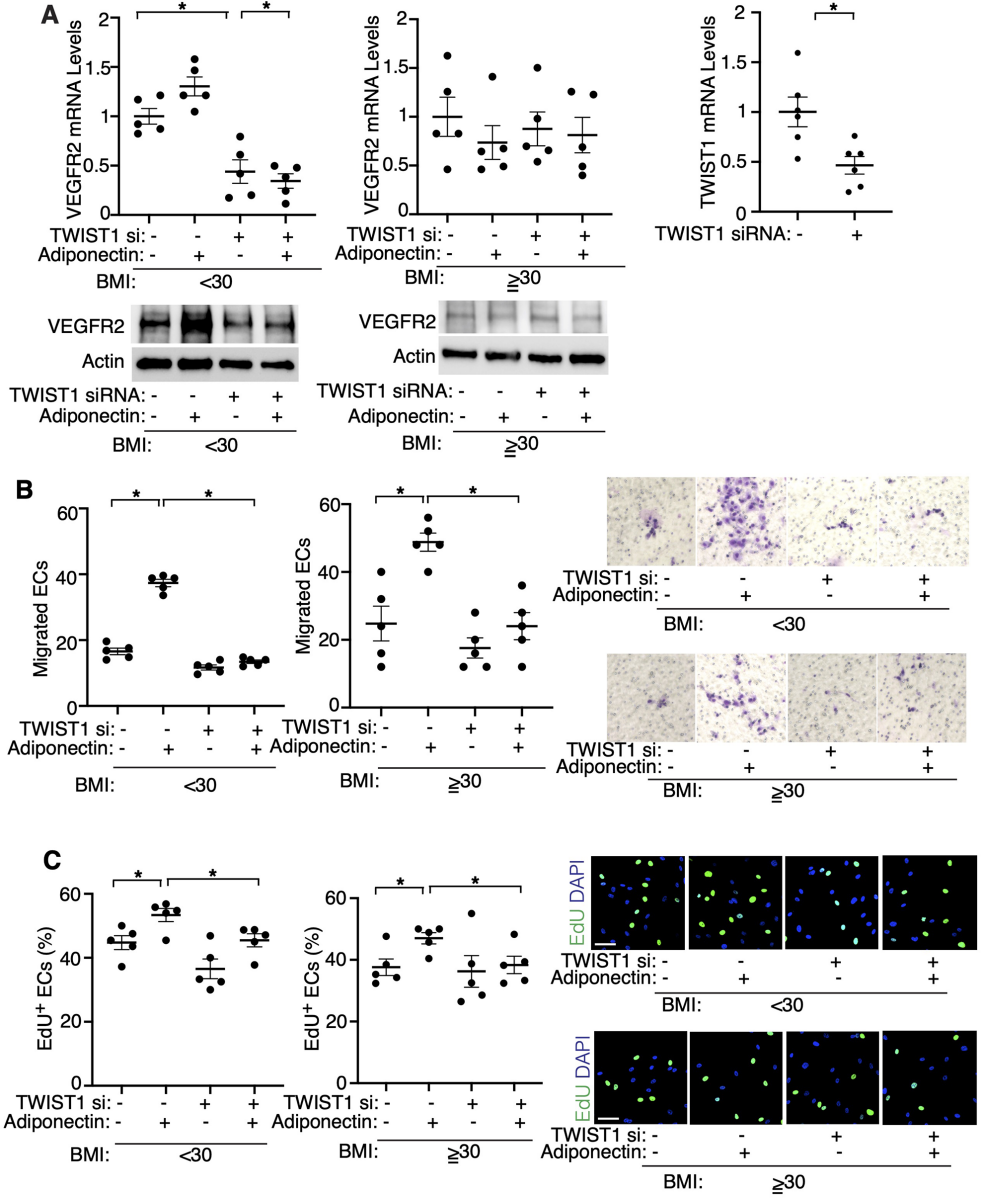
Adiponectin mediates angiogenic abilities in human lung ECs *in vitro*. **(A)** Graphs showing the mRNA levels of VEGFR2 in lean (BMI < 30, *left*) or obese (BMI ≥ 30, *middle*) human lung ECs treated with adiponectin or in combination with TWIST1 siRNA (*n* = 5, mean ± s.e.m., *p < 0.05). Graph showing the mRNA levels of TWIST1 in human lung ECs treated with TWIST1 siRNA (*right*, *n* = 6, mean ± s.e.m., *p < 0.05). IB showing the levels of VEGFR2 and Actin in lean (*left*) or obese (*right*) human lung ECs treated with adiponectin or in combination with TWIST1 siRNA. **(B)** Graphs showing migration of lean (*left*) or obese (*right*) human lung ECs treated with adiponectin or in combination with TWIST1 siRNA (*n* = 5, mean ± s.e.m., *p < 0.05). Representative images of migrated ECs. **(C)** Graphs showing DNA synthesis of lean (*left*) or obese (*right*) human lung ECs treated with adiponectin or in combination with TWIST1 siRNA (*n* = 5, mean ± s.e.m., *p < 0.05). Representative images of EdU^+^ ECs and DAPI.

To further examine the effects of adiponectin-TWIST1 signaling on EC behaviors, we isolated ECs from lean (BMI < 30) or obese (BMI > 30) human lungs as we reported (17, 19, 25, 36) (Table 1), treated ECs with adiponectin (300 ng/ml) or in combination with TWIST1 siRNA and investigated the effects. Adiponectin increased the mRNA and protein levels of VEGFR2, as well as stimulated DNA synthesis and migration in lean lung ECs; in contrast siRNA-based knockdown of TWIST1 attenuated the effects (Figures 4A–C). Adiponectin also stimulated DNA synthesis and migration in the obese lung ECs, which was inhibited by TWIST1 knockdown; however, adiponectin failed to increase VEGFR2 expression in the obese lung ECs (Figures 4A–C).

**Figure 5:**
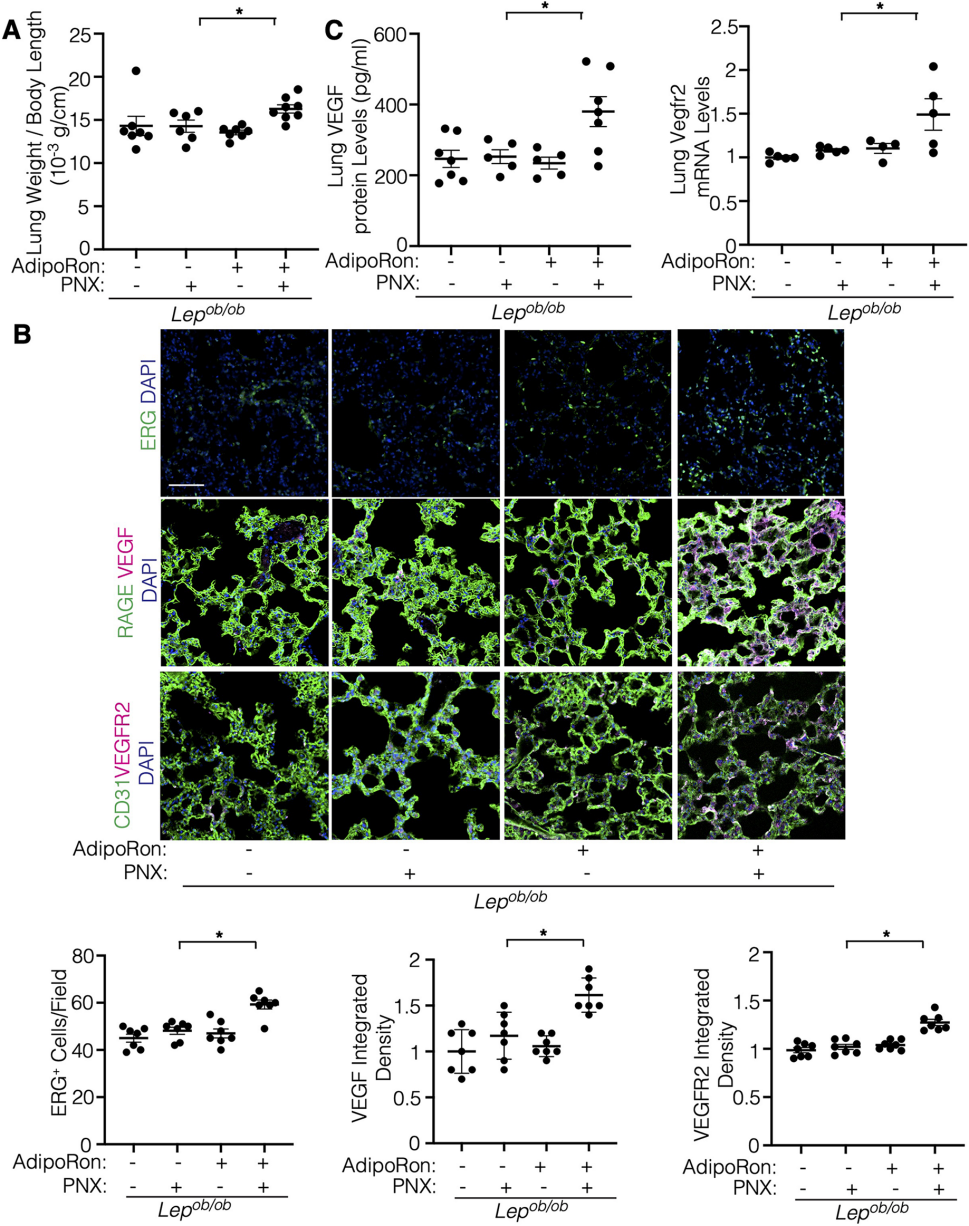
Adiporon restores post-PNX lung growth in the *Lep^ob/ob^* mouse lungs. **(A)** Graph showing the ratio of the weight of right lung lobe to mouse body length in *Lep^ob/ob^* mice 7 days after PNX or in combination with treatment with AdipoRon (*n* = 6–8, mean ± s.e.m., *p < 0.05). **(B)** IF micrographs showing ERG-positive ECs and DAPI (*top*), RAGE-positive alveolar epithelial cells, VEGF expression and DAPI (*2nd*), and CD31-positive blood vessels, VEGFR2 expression and DAPI (*bottom*) in the *Lep^ob/ob^* mice 7 days after PNX or in combination with treatment with AdipoRon. Scale bar, 50 μm. Graphs showing the quantification of ERG-positive ECs (*left*), VEGF expression (*middle*), and VEGFR2 expression (*right*) in the *Lep^ob/ob^* mouse lungs 7 days after PNX or in combination with treatment with AdipoRon (*n* = 7, mean ± s.e.m., *p < 0.05). **(C)** Graph showing the VEGF protein levels in the *Lep^ob/ob^* mouse lungs 7 days after PNX or in combination with treatment with AdipoRon (*left*, *n* = 5-7, mean ± s.e.m., *p < 0.05). Graph showing the Vegfr2 mRNA levels in the *Lep^ob/ob^* mouse lungs 7 days after PNX or in combination with treatment with AdipoRon (*right*, *n* = 4–5, mean ± s.e.m., *p < 0.05).

We then investigated whether stimulation of adiponectin signaling induces angiogenesis and regenerative lung growth in obese mice by treating mice with AdipoRon, an agonist of adiponectin receptors AdipoR1 and AdipoR2 (39). AdipoRon (oral gavage, 150 μg/mouse) stimulated post-PNX lung growth and increased ERG^+^ EC density and expression of VEGF and VEGFR2 in *Lep^ob/ob^* obese mouse lungs after PNX when analyzed by IHC (Figures 5A,B). AdipoRon also increased the levels of VEGF and VEGFR2 in the post-PNX *Lep^ob/ob^* mouse lungs compared to that without treatment (Figure 5C). These results suggest that adiponectin signaling increases VEGF-VEGFR2 expression and restores lung vascular and alveolar regeneration in obese mice.

## Discussion

**Figure 6:**
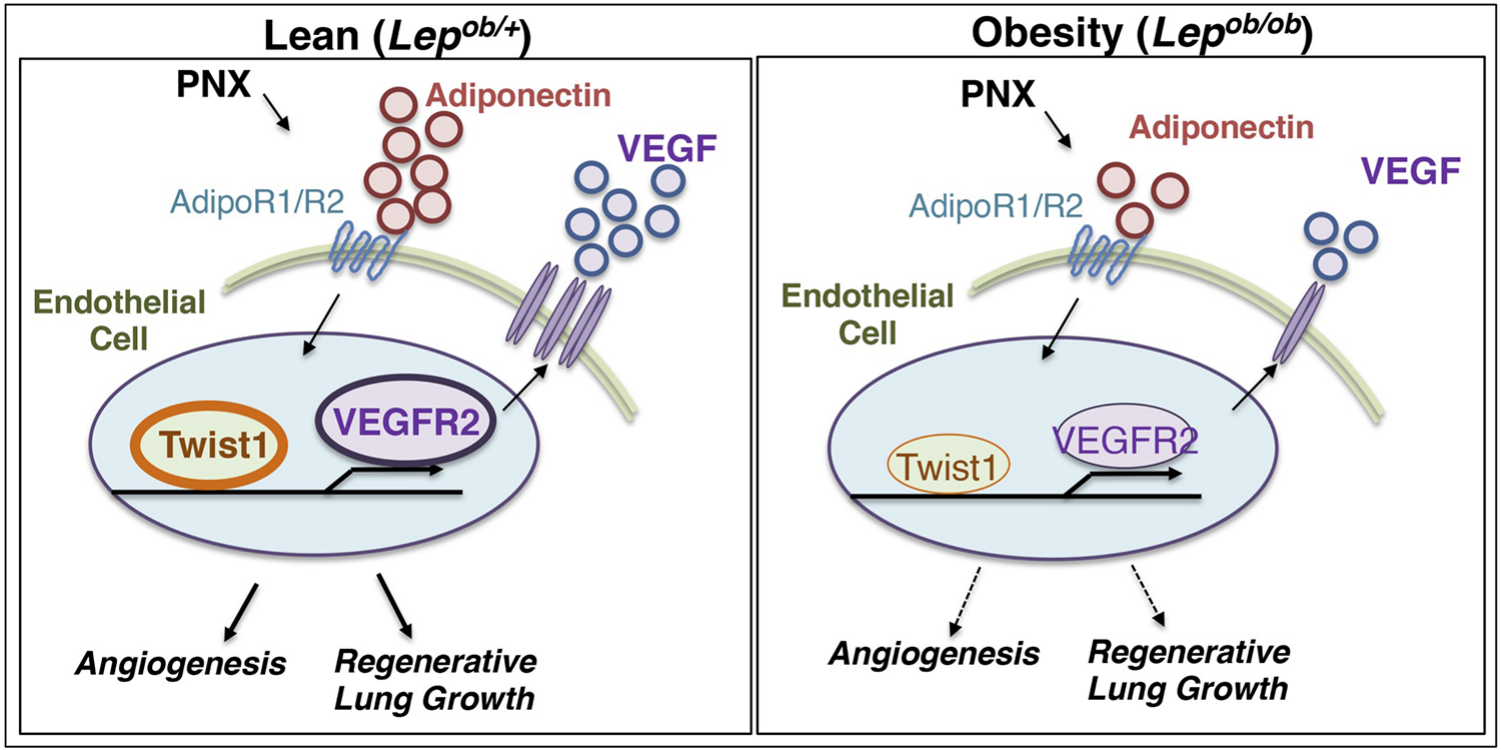
Schematic illustration of the angiogenic signaling pathway in lean vs. obese lungs. Schematic illustration showing that angiogenesis and regenerative lung growth after unilateral PNX mediated by adiponectin-TWIST1-VEGF/VEGFR2 signaling are inhibited in *Lep^ob/ob^* obese mice; post-PNX increases in the levels of adiponectin, TWIST1, VEGF, and VEGFR2 are suppressed in *Lep^ob/ob^* obese mice.

Here we have demonstrated that regenerative lung growth after unilateral PNX mediated by adiponectin-VEGF/VEGFR2 signaling is inhibited in *Lep^ob/ob^* obese mice (Figure 6). The levels of adiponectin, VEGF, and VEGFR2 increased after PNX were suppressed in *Lep^ob/ob^* obese mice, and post-PNX lung growth was inhibited in *Adipoq^−/−^* mice. Post-PNX increases in the levels of TWIST1 were also suppressed in *Lep^ob/ob^* and *Adipoq^−/−^* mouse lung ECs. Adiponectin stimulated angiogenic activities in lean and obese human lung ECs *in vitro*, while these effects were suppressed by TWIST1 knockdown. AdipoRon restored VEGF and VEGFR2 expression, vascular formation, and regenerative lung growth in post-PNX *Lep^ob/ob^* mice. These findings suggest that obesity impairs vascular and alveolar regeneration by suppressing adiponectin-VEGF/VEGFR2 signaling, and adiponectin may be one of the therapeutic targets to improve regenerative ability in obese lungs.

It has been reported that obesity correlates with poorer outcomes in respiratory diseases, including COVID-19 (40), acute respiratory distress syndrome (5), and idiopathic pulmonary fibrosis (IPF) (41), for which treatment options are limited. Reduced levels of circulating adiponectin and other adipokine are associated with COVID-19 severity (42). Adiponectin signaling suppresses profibrotic activation of fibroblast in IPF (43). Since impairment of angiogenesis and regenerative/repair ability contributes to lung disease pathology (24), modulating the adiponectin-VEGF/VEGFR2 signaling may also improve the outcomes of obesity-related lung diseases.

Interaction between epithelial cells and capillary ECs is necessary for organ regeneration and repair (24). Perturbation of the levels of angiogenic factors (3) and suppression of angiogenesis mediate impairment of wound healing and tissue repair in obese animals (20). VEGF signaling is necessary for regenerative lung growth after PNX (13). Since VEGF mainly expresses in AT1 cells (37), while VEGFR2 in ECs (35), crosstalk of AT1 cells and ECs controls vascular regeneration after PNX. Other angiogenic signaling may also be involved in the mechanism. We have reported that angiopoietin (Ang)-Tie2 signaling mediates post-PNX lung growth (17). Ang2 and Tie2 expression increased after PNX in *Lep^ob/+^* mice, which was inhibited in *Lep^ob/ob^* obese mouse lungs (Supplementary Figure S1D). Ang2 and Tie2 are involved in subcutaneous adipose tissue remodeling and subsequently contribute to systemic glucose tolerance and metabolic homeostasis (44). The plasma levels of Ang2 and soluble Tie2 are increased in pediatric obstructive sleep apnea and obesity (45). In addition, it is reported that angiopoietin-like proteins (ANGPTL-2, -3, -4, -6, -8), which potently control angiogenesis, regulate lipid, glucose and energy metabolism independent of angiogenic effects (46). For example, ANGPTL-2 mediates obesity and related metabolic diseases (47). Thus, in addition to VEGF signaling, other angiogenic and metabolic signaling may directly or indirectly contribute to the inhibition of lung regeneration in obese people. During lung development, VEGF is distributed uniformly throughout the airway epithelium in the early embryonic stages, while in the later stage the expression is restricted to the growing tips of airway branches in the distal lung where new blood vessels are recruited (48). Spatiotemporal changes in the distribution of angiogenic factors may also mediate inhibition of regenerative lung growth in the obese lungs.

Alternate splicing of the murine Vegf mRNA results in three isoforms (120, 164, and 188). Vegf120 isoform is diffusible, while Vegf164 and 188 isoforms bind to heparan sulfate on the cell surface or in the extracellular matrix (49). It is reported that lung vascular and alveolar structures are disrupted in mice only expressing Vegf120 compared with wild-type littermates expressing all three isoforms, suggesting the involvement of Vegf164 and 188 isoforms in lung microvascular and alveolar development (49, 50). In fact, the levels of Vegf164 significantly increased in the *Lep^ob/+^* lung after PNX; in contrast, it was inhibited in *Lep^ob/ob^* obese mice (Supplementary Figure S1C). In the adult, Vegf188 expression is the highest in lung and heart (49) and Vegf188 may also play active roles in lung regeneration; however, the levels of Vegf120 or 188 did not significantly change during lung growth after PNX (not shown). Although isoform-specific antibodies/probes are not available due to only 24 amino acids difference, further time course analysis of mRNA levels will elucidate the isoform-specific effects on obesity-dependent inhibition of lung regeneration.

Our results suggest that adiponectin is necessary for the expression of VEGF in AT1 cells and VEGFR2 in ECs, mediating vascular and alveolar regeneration after PNX (Figure 3). Adiponectin may induce angiogenesis through other signaling pathways as well (e.g., AMPK, eNOS) (3, 26). Suppression of adiponectin signaling also decreases PGC1α expression, which inhibits angiogenesis and mitochondrial biogenesis that suppresses tissue metabolism (18, 51). The levels of adiponectin increased in the post-PNX mouse lungs and interacted with angiogenesis- and metabolism-related genes (Figure 2). It remains unknown the mechanism by which adiponectin expression increases after PNX. Adiponectin is produced mainly by adipocytes (30). Post-PNX signaling in the lungs may control adiponectin expression in adipose tissue, which controls angiogenic signaling in the lung. Adiponectin is also produced by other cell types, such as skeletal- and cardio-myocytes and ECs (30), and exhibits energy metabolism, anti-diabetic, and anti-inflammatory effects (26, 27). It is reported that lipids are stored in lipid-laden adipocyte-like cells known as lipofibroblasts in the lung. Lipofibroblasts in the lung may also secrete adiponectin and regulate alveolar lipid homeostasis as well as pulmonary surfactant production (52) to stimulate lung development and regeneration, which may be impaired in obese animals.

Obesity contributes to various diseases such as hypertension, atherosclerosis and COPD, in which mechanical environment and EC signaling are involved in the disease progression (9, 53). TWIST1 is involved in the obesity- and angiogenesis-associated diseases such as pulmonary fibrosis (34), pulmonary hypertension (36), and atherosclerosis (53). TWIST1 regulates vascular development and regeneration (18, 25, 33, 34) by changing various angiogenic signaling (e.g., VEGF-VEGFR2, Ang-Tie2). We have reported that angiogenesis is impaired in obese human subcutaneous adipose tissue ECs through TWIST1-SLIT2 signaling (25). Increased TWIST1 expression in aged ECs decreases the expression of PGC1α to suppress mitochondrial biogenesis, leading to inhibition of regenerative lung growth and vascular formation in aged mouse lungs (18). TWIST1 is also involved in cellular senescence (36) as well as epithelial/endothelial to mesenchymal transition (EMT/EndMT), which is involved in atherosclerosis (53, 54). It has been reported that EMT/EndMT mediates lung development and regeneration (55). Thus, TWIST1 may mediate inhibition of post-PNX lung growth in obese mice by changing multiple signaling pathways.

In addition to chemical growth factors, the micromechanical environment controls vascular formation and function (24, 35). It has been demonstrated that mechanical forces change during post-PNX lung growth (15, 16, 19). We have reported that hydrostatic pressure increases after PNX, which stimulates post-PNX angiogenesis in the lung (19). In fact, TWIST1 and another mechanosensitive transcription co-activator YAP1 sense mechanical stimuli (*e.g.,* cell density, ECM stiffness, mechanical tension, flow) (56) and control vascular and alveolar regeneration after PNX (16–19, 53). In addition, a low serum adiponectin level is associated with arterial stiffness in hypertensive patients (57). Mechanical stretching also controls adiponectin protein expression in mesenchymal stem cells (58). Here we have demonstrated that **(1)** TWIST1 expression induced after PNX is inhibited in *Lep^ob/ob^* obese mouse or *Adipoq^−/−^* mouse lungs (Figures 2, 3) and **(2)** adiponectin increases VEGFR2 expression and migration/proliferation of human lung ECs, while the effects are inhibited by TWIST1 knockdown (Figure 4). Thus, post-PNX changes in the mechanical forces may stimulate adiponectin signaling and control vascular and alveolar regeneration in the lung through TWIST1-VEGFR2 signaling. Although AdipoRon restored VEGF and VEGFR2 expression and lung vascular and alveolar regeneration after PNX in obese mice (Figure 5), adiponectin did not induce VEGFR2 expression in obese human lung ECs in culture even it restores EC migration and proliferation (Figure 4). This may be because of the differences in the experimental condition (human and mouse lungs, *in vitro* and *in vivo*), in which mechanical and chemical environment are altered. Alternatively, different angiogenic signaling may be involved in the mechanism.

In this study, we used *Lep^ob/ob^* obese mice, in which leptin gene is mutated. Leptin is a neuroendocrine hormone that is secreted mainly from adipose tissue and acts primarily on brain, regulating metabolism and fat accumulation (52). Further investigation using a more physiological high fat diet-induced obese mouse model will uncover the direct metabolic effects on regenerative lung growth.

In summary, we have demonstrated that lung vascular and alveolar regeneration after unilateral PNX is inhibited in *Lep^ob/ob^* obese mice through suppression of adiponectin-VEGF/VEGFR2 signaling. Post-PNX regenerative lung growth is inhibited in *Lep^ob/ob^* obese mice or *Adipoq^−/−^* mice, while AdipoRon restores the effects. Modulation of adiponectin-VEGF/VEGFR2 signaling may improve the way for lung regeneration and potentially lead to the development of new therapeutic strategies for chronic lung diseases in obese patients.

## Data Availability

The datasets presented in this study can be found in online repositories. The names of the repository/repositories and accession number(s) can be found in the article/Supplementary Material.

## Supplementary material

Supplementary Figure S1 Vegf/Vegfr2 and Adipor expression in post-PNX mouse lungs. **(A)** Representative FACS plots showing CD31^+^ cells (fraction P2) in the mouse lungs. **(B)** Graph showing the Vegfr2 mRNA levels in the *Lep^ob/+^* or *Lep^ob/ob^* mouse lungs 7 days after PNX (*n* = 5–6, mean ± s.e.m., **p* < 0.05). **(C)** Graph showing the Vegf164 mRNA levels in the *Lep^ob/+^* or *Lep^ob/ob^* mouse lungs 7 days after PNX (*n* = 5, mean ± s.e.m., **p* < 0.05). **(D)** Graph showing the Ang2 and Tie2 mRNA levels in the *Lep^ob/+^* or *Lep^ob/ob^* mouse lungs 7 days after PNX (*n* = 5–6, mean ± s.e.m.). **(E)** Graph showing the Adipor1 and 2 mRNA levels in the *Lep^ob/+^* or *Lep^ob/ob^* mouse lungs 7 days after PNX (*n* = 5–7, mean ± s.e.m.). **(F)** Graph showing the Vegfr2 mRNA levels in the *Adipoq^−/−^* mouse lung ECs 7 days after PNX (*n* = 5–6, mean ± s.e.m.).

Supplementary Figure S2 Original gel images of Figure 4A.
