# Supplementary figures and images for: Inhibition of angiogenesis and regenerative lung growth in Lepob/ob mice through adiponectin-VEGF/VEGFR2 signaling
Source: Front Cardiovasc Med. 2024 Oct 16;11:1491971. doi: 10.3389/fcvm.2024.1491971 (PMC11521822; doi:10.3389/fcvm.2024.1491971)

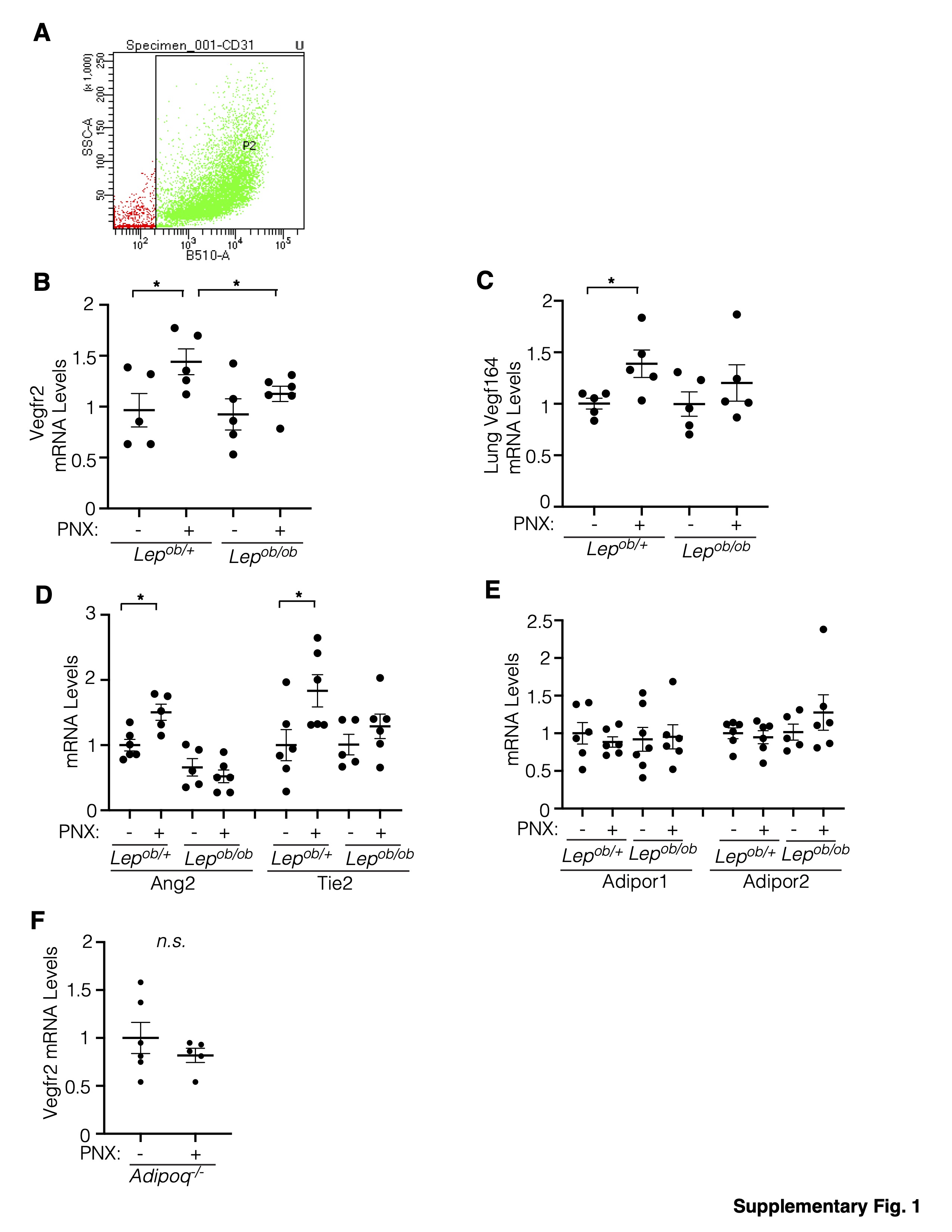

Supplement: Supplementary Figure S1 — Vegf/Vegfr2 and Adipor expression in post-PNX mouse lungs. (A) Representative FACS plots showing CD31+ cells (fraction P2) in the mouse lungs. (B) Graph showing the Vegfr2 mRNA levels in the Lepob/+ or Lepob/ob mouse lungs 7 days after PNX (n = 5–6, mean ± s.e.m., *p < 0.05). (C) Graph showing the Vegf164 mRNA levels in the Lepob/+ or Lepob/ob mouse lungs 7 days after PNX (n = 5, mean ± s.e.m., *p < 0.05). (D) Graph showing the Ang2 and Tie2 mRNA levels in the Lepob/+ or Lepob/ob mouse lungs 7 days after PNX (n = 5–6, mean ± s.e.m.). (E) Graph showing the Adipor1 and 2 mRNA levels in the Lepob/+ or Lepob/ob mouse lungs 7 days after PNX (n = 5–7, mean ± s.e.m.). (F) Graph showing the Vegfr2 mRNA levels in the Adipoq−/− mouse lung ECs 7 days after PNX (n = 5–6, mean ± s.e.m.). [file Image1.jpeg]

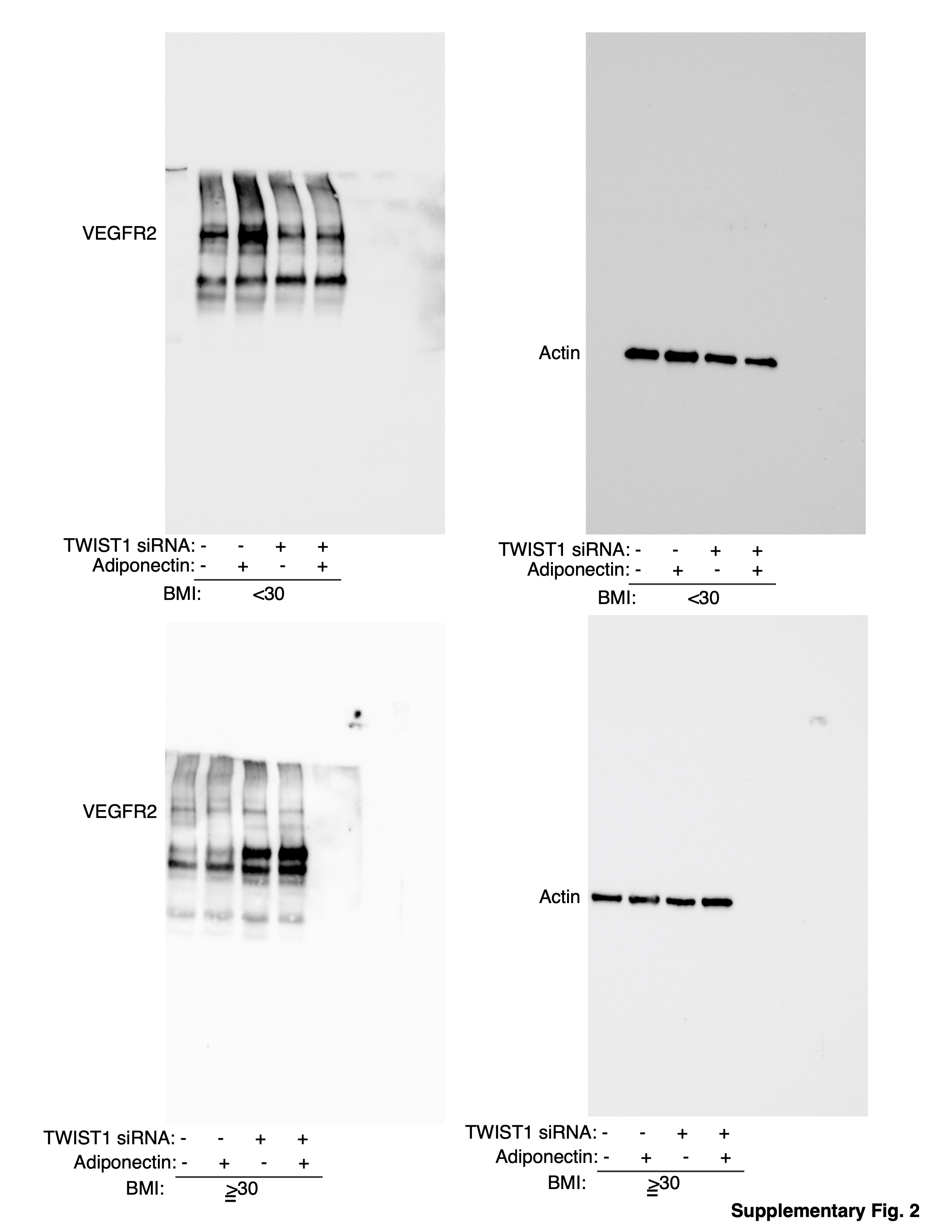

Supplement: Supplementary Figure S2 — Original gel images of Figure 4A. [file Image2.jpeg]
