# Supplementary material for: Inhibition of angiogenesis and regenerative lung growth in Lepob/ob mice through adiponectin-VEGF/VEGFR2 signaling
Source: Front Cardiovasc Med. 2024 Oct 16;11:1491971. doi: 10.3389/fcvm.2024.1491971 (PMC11521822; doi:10.3389/fcvm.2024.1491971)
Supplement: Supplementary file 3 [file Datasheet1.pdf]

name	log2FoldChange	pvalue	padj
Adamts16	8.646050855	9.726469971	1.88E-10
Car6	7.364049017	6.663745785	2.17E-07
Chga	7.022039711	8.347740974	4.49E-09
Msx3	6.857460522	5.168794672	6.78E-06
Adipoq	6.598163104	7.288435136	5.15E-08
Saa3	6.439098783	59.52957256	2.95E-60
H2-M11	6.300930509	4.474994082	3.35E-05
Col24a1	5.947883254	45.22230798	5.99E-46
Col11a1	5.894696647	3.513973055	0.000306
Gm14461	5.724080892	2.897637962	0.001266
Arg1	5.61029182	20.78667311	1.63E-21
Cfd	5.593932012	8.382180534	4.15E-09
Bcat1	5.541331274	4.594264324	2.55E-05
Tpsb2	5.49600252	13.91427747	1.22E-14
Ecel1	5.466564403	4.988337244	1.03E-05
Gabrb3	5.454211215	2.793568001	0.001609
Pnoc	5.451712664	4.907172489	1.24E-05
Clca2	5.41605499	18.10801803	7.80E-19
Epyc	5.325028884	2.196240518	0.006364
Mrgprb1	5.320283779	2.829611416	0.00148
Vsig4	5.29822979	59.65450113	2.22E-60
Cd5l	5.176515595	70.85982146	1.38E-71
Pck1	4.967648553	8.145707509	7.15E-09
Car3	4.945613544	17.79534676	1.60E-18
Chl1	4.91903194	50.44699891	3.57E-51
Ccl8	4.918820504	14.49625751	3.19E-15
Dlk1	4.877144148	7.016917714	9.62E-08
Prss35	4.80340054	41.46260277	3.45E-42
Pdzrn4	4.685352213	2.226090334	0.005942
H2-M9	4.626969454	7.080714923	8.30E-08
Ccl24	4.57283086	14.11466503	7.68E-15
Ucp1	4.572209088	7.418975926	3.81E-08
Sez6l	4.514815208	18.47406703	3.36E-19
Fam64a	4.483982047	42.93826803	1.15E-43
Tph1	4.299232607	5.950915216	1.12E-06
Lum	4.29299146	33.60643886	2.47E-34
Cxcl5	4.213163944	14.18076816	6.60E-15
Kcnt1	4.105679478	13.48397115	3.28E-14
Tpsab1	4.06137062	2.720184821	0.001905
Nxpe5	4.040341571	42.70454738	1.97E-43
Ckap2	4.018107792	21.67708112	2.10E-22
Mmp10	3.949517667	4.798779002	1.59E-05
Col6a5	3.937512953	23.32489219	4.73E-24
Pif1	3.932141854	32.2128056	6.13E-33
Fabp7	3.906551561	10.40757346	3.91E-11
Twist1	3.898350299	8.608259165	2.46E-09
Angptl7	3.892811339	24.89026956	1.29E-25

Troap	3.819718584	29.99476368	1.01E-30
Ankle1	3.809777921	17.98938502	1.02E-18
Fcna	3.801314547	41.58105747	2.62E-42
Iqgap3	3.779937408	16.30271552	4.98E-17
Pbk	3.760074477	29.60376694	2.49E-30
Cenpf	3.75676649	26.80382634	1.57E-27
Lgals7	3.725863826	20.5159649	3.05E-21
Fgf23	3.704737828	5.357729533	4.39E-06
Adamts19	3.685480504	9.563537389	2.73E-10
Etv4	3.683291722	10.43201601	3.70E-11
Rph3a	3.627519417	2.891331445	0.001284
Lrrc15	3.621956623	7.628099034	2.35E-08
Krt14	3.604720425	35.66591903	2.16E-36
Col6a6	3.592624805	8.144468119	7.17E-09
Cma1	3.591970601	27.26910438	5.38E-28
Srrm4	3.578842495	8.073292528	8.45E-09
Thbs2	3.550906115	15.67702621	2.10E-16
Gjb4	3.542531802	7.810366752	1.55E-08
Ube2c	3.538845007	61.607501	2.47E-62
Cdkn3	3.537958999	39.71925605	1.91E-40
Mirg	3.513024306	5.531734568	2.94E-06
Timp1	3.501027915	47.90437513	1.25E-48
Cemip	3.499228047	7.978317712	1.05E-08
Alppl2	3.494148615	2.202729282	0.00627
Kif18b	3.493246626	15.700063	1.99E-16
Ttk	3.479222828	63.61759983	2.41E-64
Gjb5	3.470214037	9.231774812	5.86E-10
Sapcd2	3.444397598	18.58021856	2.63E-19
Prc1	3.420562129	23.22267811	5.99E-24
Cep55	3.40664297	59.95055604	1.12E-60
BC030867	3.393874796	8.061596158	8.68E-09
Has2	3.363764912	9.035882767	9.21E-10
Lin7a	3.33598005	4.111033791	7.74E-05
Fhl5	3.305854799	3.079476906	0.000833
Gprin1	3.300992523	2.150432429	0.007072
Egr4	3.300920961	4.427107769	3.74E-05
Vat1l	3.294648596	4.228376481	5.91E-05
Nfasc	3.290873055	3.750091254	0.000178
Cdk1	3.276843995	37.22024024	6.02E-38
Wt1os	3.27120258	3.31953579	0.000479
Hmmr	3.261357844	28.7593201	1.74E-29
Cdc25c	3.257010959	10.46895745	3.40E-11
Vit	3.240627754	6.721030028	1.90E-07
Nuf2	3.237737173	17.41949827	3.81E-18
Spag5	3.217905169	16.73636303	1.84E-17
Sntg1	3.216315	2.194388703	0.006392
Ccnb1	3.206194651	28.42603708	3.75E-29
Clgn	3.205482878	3.762512414	0.000173

Depdc1a	3.192401441	49.20272968	6.27E-50
Tpx2	3.175035334	28.91295091	1.22E-29
Lrrn1	3.173281242	3.922028767	0.00012
Ska1	3.171597508	14.93472985	1.16E-15
Cxcl13	3.16206413	10.63344724	2.33E-11
4930558J18Rik	3.158816262	3.926686128	0.000118
Kifc1	3.147626537	32.15432028	7.01E-33
Kntc1	3.134762328	19.68343473	2.07E-20
Has2os	3.119598046	3.303912914	0.000497
Kif22	3.090172862	17.33579035	4.62E-18
Ckap2l	3.064202192	24.80192371	1.58E-25
Melk	3.057951466	17.25933659	5.50E-18
Plk1	3.05504288	18.39448387	4.03E-19
Ccnb2	3.052538279	24.68607557	2.06E-25
Fam83d	3.051289055	48.49571934	3.19E-49
Cenpm	3.050714061	11.39477831	4.03E-12
Cdc20	3.039133314	36.5673555	2.71E-37
Calca	3.031558378	8.580406622	2.63E-09
Aspm	2.997879568	15.83088989	1.48E-16
Birc5	2.981846304	13.38558124	4.12E-14
Kif2c	2.973458092	19.66481201	2.16E-20
Zbtbd6	2.951486781	9.668146962	2.15E-10
Anln	2.941373636	28.70433671	1.98E-29
Top2a	2.932903037	17.29516115	5.07E-18
Slfn9	2.932894081	54.03228157	9.28E-55
Prr11	2.932300513	37.83541957	1.46E-38
Cdca8	2.931739632	16.81837991	1.52E-17
Knstrn	2.922675632	43.052358	8.86E-44
Sgol2	2.921986345	70.64698509	2.25E-71
Sgol1	2.915687156	43.65823807	2.20E-44
Neil3	2.905755365	14.47834145	3.32E-15
Mastl	2.896768127	30.48340154	3.29E-31
Ska3	2.896040032	28.74730296	1.79E-29
Stil	2.886650637	11.06629229	8.58E-12
Cdca2	2.88552974	29.29683137	5.05E-30
Calcb	2.88485176	14.92161318	1.20E-15
Folr2	2.881605731	8.727338764	1.87E-09
Aurkb	2.878187692	14.51068575	3.09E-15
Col5a3	2.8769208	22.74165891	1.81E-23
Xkr5	2.872358868	6.53338117	2.93E-07
Bai2	2.864062352	9.378773535	4.18E-10
Pmch	2.861179062	9.231774812	5.86E-10
Esco2	2.859112634	33.94258198	1.14E-34
Shcbp1	2.848789469	13.27419814	5.32E-14
AF357359	2.841293009	3.074630735	0.000842
Mtfr2	2.835141388	10.61083925	2.45E-11
Parpbp	2.821017071	19.80908067	1.55E-20
Tnc	2.82096034	39.67892455	2.09E-40

Mki67	2.815910772	12.88989063	1.29E-13
Arhgef39	2.814920473	19.24755614	5.66E-20
4933413G19Rik	2.814347304	7.807715965	1.56E-08
Rpl39l	2.812887499	2.394090372	0.004036
Cdhr1	2.80822425	2.555916179	0.00278
Kif11	2.807454731	21.43070691	3.71E-22
Meg3	2.806330471	22.732793	1.85E-23
Cdca3	2.80581623	11.51147214	3.08E-12
Cenpi	2.79925489	37.45167756	3.53E-38
Scara3	2.787998807	7.062944367	8.65E-08
Pappa2	2.784789008	3.87011793	0.000135
Nusap1	2.784260544	24.41268194	3.87E-25
Vmn2r26	2.784183119	2.714524621	0.00193
Serpinb2	2.783908374	5.058990447	8.73E-06
Cyp26a1	2.782335256	4.641967554	2.28E-05
Dtl	2.77697278	41.56537293	2.72E-42
Cyp1b1	2.776417204	27.57756336	2.65E-28
Adamts4	2.773500136	13.20630862	6.22E-14
Rbm44	2.770973346	4.594944674	2.54E-05
Psrc1	2.769901954	8.386934941	4.10E-09
Foxm1	2.769713333	13.21511626	6.09E-14
Ccl7	2.765744486	28.74843632	1.78E-29
Rad51ap1	2.764917818	36.00135414	9.97E-37
Casc5	2.755084985	29.70281326	1.98E-30
Lhx2	2.753367755	2.510911602	0.003084
Gjb3	2.746501874	5.425839684	3.75E-06
Olfr558	2.732739623	3.860652843	0.000138
Ocstamp	2.727734864	3.476548069	0.000334
Rian	2.727434158	45.64560331	2.26E-46
Cenpe	2.718338288	33.76117579	1.73E-34
Hist1h1b	2.716666615	3.989076818	0.000103
Cdca5	2.715456692	19.94243205	1.14E-20
Mir147	2.702508185	16.40071052	3.97E-17
Ect2	2.702263875	46.96836278	1.08E-47
Clspn	2.698988415	7.66818247	2.15E-08
Mir1906-2	2.690984574	11.46296322	3.44E-12
Mir1906-1	2.690984574	11.46296322	3.44E-12
Kif14	2.680148758	8.711620402	1.94E-09
Clhc1	2.678279267	12.2162575	6.08E-13
C1qc	2.677221092	20.83344401	1.47E-21
Mis18bp1	2.669821039	20.08610622	8.20E-21
Cilp2	2.663623399	2.323797485	0.004745
Diap3	2.655112519	12.62411125	2.38E-13
C1qb	2.652807893	18.01332841	9.70E-19
Camk1g	2.650928065	2.740081765	0.001819
Marco	2.644509623	13.53988719	2.88E-14
Dlgap5	2.640898974	16.85605736	1.39E-17
Spc24	2.633764727	15.18587633	6.52E-16

Phf19	2.620638341	18.6951021	2.02E-19
Mcpt4	2.618751893	4.119189209	7.6E-05
E2f7	2.618709814	22.5571336	2.77E-23
Cdh3	2.607361104	5.86392651	1.37E-06
Cenph	2.60536509	5.74813222	1.79E-06
Ebf2	2.600630872	4.530351405	2.95E-05
Cxcl1	2.59768598	9.867020598	1.36E-10
Gtse1	2.588869276	19.36914491	4.27E-20
Bub1	2.586481286	26.71150705	1.94E-27
Aurka	2.581661201	38.25811019	5.52E-39
Ccnf	2.58006084	23.06220133	8.67E-24
Dnph1	2.564632242	12.69144122	2.03E-13
Oip5	2.563611254	31.57419036	2.67E-32
Wt1	2.562292346	6.535596191	2.91E-07
Lrr1	2.561842556	5.431845706	3.7E-06
Ccna2	2.55645763	59.47711995	3.33E-60
Gpr133	2.552731056	4.363797512	4.33E-05
Rad51	2.54956794	29.79800705	1.59E-30
Uhrf1	2.549334607	10.43522828	3.67E-11
Ndc80	2.546943875	29.76299038	1.73E-30
Cacna1g	2.538084466	5.824447066	1.5E-06
Mir5134	2.537584687	3.668412815	0.000215
Prrx2	2.535511256	10.91748162	1.21E-11
Exo1	2.535424138	11.21831381	6.05E-12
Cdc6	2.535392951	17.11619509	7.65E-18
Nr1h4	2.534397249	3.658240806	0.00022
C1qa	2.527782315	16.60002969	2.51E-17
AA467197	2.523798956	11.02239387	9.50E-12
Serpinf1	2.520435714	8.72302958	1.89E-09
Racgap1	2.519964978	10.99409469	1.01E-11
C330027C09Rik	2.513043996	46.09330778	8.07E-47
Dscc1	2.508573434	9.556669027	2.78E-10
Vcan	2.504046761	18.221563	6.00E-19
Ncapg	2.497541814	40.48781681	3.25E-41
A2m	2.490615004	6.188195544	6.48E-07
4930427A07Rik	2.489858317	13.94086428	1.15E-14
Ccdc18	2.48662938	22.58589498	2.59E-23
Cd109	2.482863637	14.51001955	3.09E-15
Fignl1	2.481361309	45.90700381	1.24E-46
Ccl12	2.474783472	12.39306882	4.05E-13
C3ar1	2.462879805	13.72766295	1.87E-14
H1fx	2.461081825	28.31461017	4.85E-29
Gpr88	2.458510362	4.999402062	1E-05
Msr1	2.451525909	8.318893465	4.80E-09
1190002F15Rik	2.450931717	15.83953407	1.45E-16
Cd163	2.440998259	8.739021042	1.82E-09
Kifc5b	2.436385152	20.16612173	6.82E-21
Capn6	2.43226166	11.99698212	1.01E-12

Rrm2	2.424345592	12.49164171	3.22E-13
Depdc1b	2.423595103	16.61140812	2.45E-17
Rmi2	2.422333708	13.27949902	5.25E-14
Espl1	2.409186362	9.102799995	7.89E-10
Kif15	2.400986183	14.38513574	4.12E-15
Col1a1	2.400950374	16.27777693	5.28E-17
2810417H13Rik	2.399397755	28.3570864	4.39E-29
2810408I11Rik	2.397042536	2.555916179	0.00278
Mrap	2.388381085	3.555936046	0.000278
Plekhg4	2.386387831	13.17544534	6.68E-14
Ms4a7	2.375838213	10.68930567	2.05E-11
Prnd	2.369501865	6.697090504	2.01E-07
Cst9	2.36900237	4.627730396	2.36E-05
Zwilch	2.367268685	15.41564996	3.84E-16
Ccne1	2.36155468	9.657674102	2.20E-10
Lctl	2.359304304	9.413810164	3.86E-10
Spdl1	2.358286977	24.39980245	3.98E-25
Rad54b	2.357719661	18.999494	1.00E-19
Col3a1	2.354803874	30.71356691	1.93E-31
Brca1	2.350172566	11.50671667	3.11E-12
Sdsl	2.336031412	3.404001232	0.000394
2700099C18Rik	2.335919856	31.39743126	4.00E-32
Pole	2.333163256	8.861617999	1.38E-09
Gdf6	2.323025334	4.766294401	1.71E-05
Ntrk1	2.316430883	4.385299022	4.12E-05
Tacc3	2.315670531	8.135519141	7.32E-09
Wfdc17	2.313006308	5.074847979	8.42E-06
Steap4	2.304010779	6.83749097	1.45E-07
P4ha3	2.303075178	14.39546343	4.02E-15
Mcm10	2.302617346	9.554673183	2.79E-10
Dock3	2.301098632	2.367279882	0.004293
Fsbp	2.297168326	10.27515071	5.31E-11
Trip13	2.296204708	20.75115423	1.77E-21
Wisp1	2.292622848	17.4303651	3.71E-18
Tk1	2.288819822	7.845402683	1.43E-08
Adamts12	2.281112031	26.95523732	1.11E-27
Fbn2	2.279938373	8.07472542	8.42E-09
Ptx3	2.277357043	12.43719999	3.65E-13
Cfb	2.271717007	2.871482308	0.001344
Chek1	2.271155462	24.12974234	7.42E-25
Cenpn	2.265190344	11.16156992	6.89E-12
Lypd1	2.252576038	7.471632262	3.38E-08
Slurp1	2.250553226	3.457929551	0.000348
Spc25	2.245059168	55.14840694	7.11E-56
Ogdhl	2.242392684	2.775584839	0.001677
Nsl1	2.242041235	12.18575144	6.52E-13
Folr1	2.238769091	2.62176196	0.002389
Ube2t	2.236756182	18.10248911	7.90E-19

E2f8	2.236640875	4.229334696	5.9E-05
Has1	2.235286217	11.29828968	5.03E-12
Cebpe	2.227094989	2.366149584	0.004304
Vsig8	2.225120022	2.735592409	0.001838
Gpr39	2.2241686	7.446910456	3.57E-08
Ebf3	2.220661202	8.482416282	3.29E-09
Ankrd55	2.216913176	5.824566538	1.5E-06
Ildr2	2.216055504	4.715003878	1.93E-05
Arsi	2.215391462	8.164014484	6.85E-09
Fcrlb	2.207781362	6.018649399	9.58E-07
Nek2	2.201923285	11.35546612	4.41E-12
Mapk10	2.200532938	2.066804349	0.008574
Scml2	2.197188587	12.17927692	6.62E-13
Ccdc80	2.196656373	9.95706212	1.10E-10
Mybl2	2.195095696	7.426754309	3.74E-08
Kif20b	2.193626831	41.31586334	4.83E-42
Dcn	2.178360057	4.400813273	3.97E-05
Slitrk4	2.175233865	2.525188749	0.002984
Smpd3	2.174174536	7.37913338	4.18E-08
Plin1	2.173657949	3.666500164	0.000216
Tnni1	2.157913864	2.133724651	0.00735
Gpr84	2.156439228	2.783037889	0.001648
Spsb4	2.14950078	5.92124509	1.2E-06
Tcf19	2.14596835	12.25913937	5.51E-13
Ncapg2	2.144857934	18.6511222	2.23E-19
Aldh1a3	2.144045239	21.92957998	1.18E-22
Pask	2.135836976	13.51194644	3.08E-14
Kif23	2.132795791	10.99099049	1.02E-11
Prtn3	2.131827537	11.71105055	1.95E-12
Gatm	2.128864145	16.66257483	2.17E-17
Rpp25	2.127762914	2.035587416	0.009213
Mir6978	2.119801986	3.668490545	0.000215
Orc1	2.11276046	6.669014349	2.14E-07
Col1a2	2.107911545	25.43493349	3.67E-26
Gfpt2	2.101047098	11.5054952	3.12E-12
Cks1b	2.084923483	22.8116203	1.54E-23
Cenpw	2.082074524	7.145376863	7.16E-08
Apol7a	2.081254112	3.050814845	0.00089
Adam12	2.080611757	25.67741702	2.10E-26
Kif4	2.07945851	8.306216929	4.94E-09
Fancd2	2.076274598	5.425839684	3.75E-06
Apitd1	2.062260793	15.24110911	5.74E-16
Kif20a	2.050497233	48.59740042	2.53E-49
Col15a1	2.045147893	7.909251343	1.23E-08
Ercc6l	2.040633215	9.776794365	1.67E-10
Cenpp	2.037592362	17.21208589	6.14E-18
Ptger3	2.033777462	13.85072051	1.41E-14
Cdc45	2.029376767	12.65817146	2.20E-13

Asf1b	2.017644252	7.765987017	1.71E-08
Ncapd2	2.014741273	7.985382931	1.03E-08
Chil4	2.013515315	4.268089111	5.39E-05
Hells	2.012054637	37.62855462	2.35E-38
Ccr5	2.010297227	29.73098887	1.86E-30
Gen1	2.009327558	13.64866741	2.25E-14
Lrrn2	2.002448563	32.06672363	8.58E-33
Trem2	1.999542923	4.687991197	2.05E-05
Insc	1.996060199	3.164219594	0.000685
Gas2l3	1.99460208	6.991402249	1.02E-07
C1qtnf6	1.989830443	43.58563861	2.60E-44
Gal3st2	1.981065198	2.175309619	0.006679
Sorcs1	1.96896601	3.120315805	0.000758
Igdcc4	1.967443465	4.989504551	1.02E-05
Prg4	1.967302575	12.19207435	6.43E-13
Ticrr	1.965441557	5.56820749	2.7E-06
Hapln1	1.962909031	2.867136864	0.001358
Igf1	1.962683671	32.03111801	9.31E-33
Lgi2	1.961289791	26.53092237	2.94E-27
Fn1	1.957574309	8.157181346	6.96E-09
Brinp1	1.953299048	5.192899176	6.41E-06
Rgs5	1.951696658	38.9854848	1.03E-39
Ptn	1.951094383	4.193734851	6.4E-05
Gpr176	1.950310989	7.625692044	2.37E-08
Trim59	1.947404184	69.61275603	2.44E-70
H19	1.947115105	11.21349027	6.12E-12
Chaf1b	1.938054473	6.551826252	2.81E-07
Egln3	1.93056356	3.929840674	0.000118
Ulbp1	1.929931419	9.970639658	1.07E-10
Fcrls	1.926061095	7.154707224	7.00E-08
Dctd	1.91375599	14.00293039	9.93E-15
Cd276	1.912263676	6.09618066	8.01E-07
Mir675	1.901134524	4.02498476	9.44E-05
Olfml2b	1.900123782	8.123881461	7.52E-09
Bub1b	1.894156677	7.727824447	1.87E-08
Fndc1	1.883061461	3.706265453	0.000197
Gins2	1.876858057	9.600386167	2.51E-10
Eln	1.874463617	79.99888178	1.00E-80
Eno2	1.868311318	4.106242356	7.83E-05
Pld5	1.867966195	6.814453978	1.53E-07
Ccna1	1.865246815	3.020223604	0.000955
Naip1	1.864989531	7.776263064	1.67E-08
Gm5577	1.864071822	4.754070397	1.76E-05
Col5a2	1.859346286	61.607501	2.47E-62
Ak5	1.854141401	3.928575503	0.000118
Cd248	1.852028951	4.992670349	1.02E-05
Flnc	1.848242031	6.284137751	5.20E-07
Agt	1.84633996	2.541227383	0.002876

Cpxm1	1.829151763	3.83147933	0.000147
Tubb3	1.825398813	4.088260216	8.16E-05
Gria2	1.82390365	7.513051247	3.07E-08
Retnla	1.820754198	8.670497016	2.14E-09
2010107G12Rik	1.81923116	2.38464236	0.004124
Crmp1	1.817301321	4.756025535	1.75E-05
Npl	1.810656651	10.11589877	7.66E-11
Psat1	1.80895795	13.78031813	1.66E-14
Cdkn2c	1.808736956	112.7656616	1.72E-113
Ephb2	1.798450235	4.105261436	7.85E-05
Enpp3	1.797542698	19.56819097	2.70E-20
Chtf18	1.795091061	7.174044599	6.70E-08
Col5a1	1.793810973	20.47411855	3.36E-21
1500015O10Rik	1.791759616	2.389789688	0.004076
Mcm5	1.791437647	12.79023884	1.62E-13
Mmp23	1.786124891	10.72185203	1.90E-11
Wasf1	1.784664688	7.996469095	1.01E-08
Tenm3	1.769241826	2.547613853	0.002834
Cenpa	1.76539153	17.1617624	6.89E-18
Raet1d	1.761483121	4.376261555	4.2E-05
Brip1	1.759271056	14.71734087	1.92E-15
Polq	1.746107518	5.266234967	5.42E-06
F2rl3	1.744330932	6.94195119	1.14E-07
Ccl2	1.743754048	28.28343063	5.21E-29
Rhou	1.722859777	14.83058657	1.48E-15
G530011O06Rik	1.719126311	9.35521081	4.41E-10
Ddias	1.715465522	13.28340979	5.21E-14
Chaf1a	1.714779013	8.513167081	3.07E-09
Smc2	1.714107208	26.02062987	9.54E-27
Vash2	1.713553041	7.113376299	7.70E-08
Traip	1.706438732	3.69065897	0.000204
Inhba	1.704757479	4.902650694	1.25E-05
Prr15	1.70455212	3.776381533	0.000167
Mest	1.703947571	7.339772995	4.57E-08
Padi4	1.70166603	3.481011963	0.00033
Tbx18	1.695197365	2.446261873	0.003579
Mmp14	1.685018468	31.40779043	3.91E-32
Mcm2	1.678427539	17.24114343	5.74E-18
Gmnn	1.670937049	39.05116114	8.89E-40
Ccl9	1.668695307	4.201114882	6.29E-05
Sox8	1.663915396	2.249951935	0.005624
Fam132b	1.655213252	3.702976489	0.000198
Bean1	1.646423998	3.402122265	0.000396
Fbln2	1.645100602	8.940078965	1.15E-09
Ccl11	1.642443438	2.360523713	0.00436
Rarres1	1.639510755	5.446331301	3.58E-06
Tyms	1.638683451	16.26727431	5.40E-17
Bard1	1.635504307	7.886036414	1.30E-08

Nfe2l3	1.623636079	3.446760363	0.000357
Pycr1	1.6218101	4.87008221	1.35E-05
Dmkn	1.618895692	4.117946423	7.62E-05
Aspn	1.617468604	5.454316193	3.51E-06
Mad2l1	1.616560818	32.91947538	1.20E-33
Shc4	1.615723593	3.927972684	0.000118
Cdc7	1.615653845	20.23171236	5.87E-21
Mmp13	1.615249905	13.72949152	1.86E-14
Adamts2	1.61444027	20.05805999	8.75E-21
Dpf1	1.613229429	2.358343445	0.004382
Fam20c	1.606257394	11.3258573	4.72E-12
Ephb1	1.601451246	3.24850248	0.000564
Cenpq	1.600037785	22.54522235	2.85E-23
Lmnb1	1.598559409	6.877624691	1.33E-07
Lama1	1.595653297	7.436949462	3.66E-08
Nodal	1.585294545	2.083132466	0.008258
Mms22l	1.580009358	12.63463256	2.32E-13
Incenp	1.579796759	9.448097095	3.56E-10
Mybl1	1.578063375	8.07472542	8.42E-09
Gins1	1.565861731	7.263897322	5.45E-08
Fscn1	1.563887372	22.10331458	7.88E-23
Ndrg4	1.555218771	6.920393899	1.20E-07
Lmnb2	1.551167051	25.2780722	5.27E-26
4930579G24Rik	1.54934547	19.50841692	3.10E-20
Clec11a	1.549032978	7.659523983	2.19E-08
Myrf	1.549005284	3.513960785	0.000306
Tmem108	1.545038106	2.563216411	0.002734
Mlxipl	1.536101181	7.075217857	8.41E-08
Cfi	1.536096972	3.035431566	0.000922
Rgs16	1.533796765	8.329220447	4.69E-09
Spon2	1.528927405	2.747186169	0.00179
Gxylt2	1.520089979	5.937048437	1.16E-06
Plk4	1.519754662	22.34924069	4.47E-23
Greb1l	1.511113964	2.093229962	0.008068
Tnfaip6	1.507640366	7.750328153	1.78E-08
Arhgap11a	1.505429949	6.057354296	8.76E-07
Pappa	1.498321049	5.698158796	2E-06
Mmp2	1.49689546	16.50128536	3.15E-17
Cit	1.494370743	5.858390749	1.39E-06
Alox15	1.493798765	3.709802188	0.000195
Mdk	1.491054512	21.8272829	1.49E-22
Lgals1	1.489581019	10.56326869	2.73E-11
Ctsk	1.481212797	2.068680226	0.008537
Loxl2	1.480385128	26.21352011	6.12E-27
Col6a4	1.474572613	4.382858736	4.14E-05
Hmgb2	1.469032714	11.18749747	6.49E-12
Tubb6	1.468868431	4.633562658	2.33E-05
Vwa1	1.46885984	20.23171236	5.87E-21

Ccsap	1.467229618	17.94422777	1.14E-18
Cks2	1.467161165	3.306952588	0.000493
Aldh1l2	1.464780407	8.416350635	3.83E-09
Cenpk	1.464716106	12.14515996	7.16E-13
Pla1a	1.464068982	6.352720784	4.44E-07
Rad54l	1.461386347	7.481590821	3.30E-08
Fstl1	1.460534015	45.78867874	1.63E-46
Pidd1	1.459718366	7.048204905	8.95E-08
Mxd3	1.459499046	9.661020498	2.18E-10
Fam167b	1.458199685	4.851233515	1.41E-05
Zbtb7c	1.458039332	4.070744627	8.5E-05
Efna5	1.457624939	9.811788919	1.54E-10
Nrcam	1.456392268	6.590559772	2.57E-07
Kcp	1.456160748	3.43650043	0.000366
Car13	1.4545875	4.12079518	7.57E-05
Gsg2	1.45268426	7.581870475	2.62E-08
Kcnd1	1.452613228	2.831889527	0.001473
Kpna2	1.452087055	25.44054366	3.63E-26
Rad51c	1.451674878	9.662521377	2.18E-10
Ror2	1.446072934	9.329942298	4.68E-10
E2f1	1.440200564	7.388561473	4.09E-08
Col6a2	1.431572843	17.88169872	1.31E-18
Spp1	1.429008823	10.37063183	4.26E-11
Fbxo5	1.427908224	35.47340547	3.36E-36
Mfap5	1.426336907	16.57838669	2.64E-17
Ccr3	1.426241293	2.388987896	0.004083
Ung	1.42093937	3.4112345	0.000388
D430020J02Rik	1.419951175	6.272679988	5.34E-07
Bora	1.415419965	13.00784801	9.82E-14
Dhfr	1.414211785	17.48039781	3.31E-18
Mir6950	1.413712656	6.866883635	1.36E-07
Ackr1	1.407052904	5.126917894	7.47E-06
Hcn1	1.40410112	2.333002564	0.004645
H2afx	1.403200278	33.38862092	4.09E-34
2610524H06Rik	1.399022653	5.94665221	1.13E-06
Gpr64	1.39762737	17.63570414	2.31E-18
Adamts7	1.393107196	13.05866475	8.74E-14
Efcab11	1.389173657	4.203212301	6.26E-05
Cadm4	1.385162614	10.80070417	1.58E-11
Dbf4	1.381611664	12.33969063	4.57E-13
Shisa4	1.379991426	13.07967417	8.32E-14
Cercam	1.373441581	2.522128903	0.003005
Dsn1	1.372993832	20.23171236	5.87E-21
Nme4	1.370033194	4.149888146	7.08E-05
Slc9a5	1.36479441	10.15997273	6.92E-11
Ear6	1.364552652	2.844855195	0.001429
Tesc	1.359908147	2.823945027	0.0015
Ms4a6d	1.355418182	5.31684014	4.82E-06

Clip3	1.352257466	3.463226552	0.000344
B4galt6	1.350328491	15.99252179	1.02E-16
Plek2	1.343769158	3.43650043	0.000366
Lig1	1.341584603	6.421315946	3.79E-07
Tuba1c	1.336516099	2.740081765	0.001819
Dbn1	1.334871238	48.50694714	3.11E-49
Cxcl3	1.334676709	5.062803437	8.65E-06
Bok	1.333723006	32.90267313	1.25E-33
Adra2a	1.332069795	2.265360291	0.005428
Gm13293	1.32685684	2.128876859	0.007432
Wnt4	1.31984905	31.67065707	2.13E-32
Hpgds	1.316946853	2.101046508	0.007924
Add2	1.313145245	2.022914978	0.009486
Rnf128	1.312658794	5.399515792	3.99E-06
Steap1	1.311618062	2.365453609	0.004311
Hmox1	1.304868492	5.482805438	3.29E-06
Skp2	1.30410056	9.476823116	3.34E-10
Abcb1b	1.301818238	4.678062579	2.1E-05
Mybpc2	1.301240646	6.179668145	6.61E-07
Kif18a	1.300785957	11.44234548	3.61E-12
Wdhd1	1.30010724	4.766912295	1.71E-05
Cenpl	1.298341175	12.32888691	4.69E-13
Atad2	1.297466725	9.819665986	1.51E-10
Ncaph	1.295370736	11.43424674	3.68E-12
Col18a1	1.294016053	11.37792085	4.19E-12
Flrt2	1.287576026	4.612585288	2.44E-05
2810408A11Rik	1.282566902	4.605432977	2.48E-05
Cdt1	1.282529198	2.61762972	0.002412
Chek2	1.281648384	11.38060976	4.16E-12
Ear7	1.280463555	2.324459456	0.004737
Mmp19	1.279193298	12.5515093	2.81E-13
Gas1	1.277411358	4.077971775	8.36E-05
Rcc1	1.276717277	10.20883256	6.18E-11
Mex3a	1.276419045	6.992038472	1.02E-07
Zgrf1	1.274421884	3.959862565	0.00011
Fen1	1.270249924	14.38513574	4.12E-15
Fbn1	1.267362849	5.298892999	5.02E-06
Shc2	1.265724326	5.382988878	4.14E-06
5730559C18Rik	1.265256528	2.731350514	0.001856
Wfdc21	1.264489179	3.70839639	0.000196
Osr1	1.262476351	14.64951423	2.24E-15
Efna4	1.260811567	3.282462962	0.000522
Nnmt	1.257564539	3.654018708	0.000222
Wnt5a	1.247929889	7.519235619	3.03E-08
Tube1	1.247360494	3.793358229	0.000161
Ezh2	1.246683482	19.33011499	4.68E-20
Ccdc34	1.2398245	16.89114403	1.28E-17
Tonsl	1.238537102	9.634922759	2.32E-10

Sparc	1.234017183	7.564800967	2.72E-08
Mfap4	1.231208638	3.932081469	0.000117
Rrm1	1.230204511	9.262199194	5.47E-10
Fam110c	1.227705033	2.753616876	0.001764
2700094K13Rik	1.226585464	19.61185999	2.44E-20
Nhs	1.22239965	5.321492502	4.77E-06
Scamp5	1.221580532	3.331442017	0.000466
Kdelr3	1.218545028	8.716247275	1.92E-09
Cfp	1.215213274	5.253640011	5.58E-06
Apoe	1.211784988	10.99282134	1.02E-11
Arhgap19	1.211431164	6.964440738	1.09E-07
Pmf1	1.207359752	6.689554334	2.04E-07
Tubb2b	1.20568321	21.30531	4.95E-22
Nid2	1.20500549	5.582212258	2.62E-06
Col16a1	1.201726826	35.56815913	2.70E-36
Ccne2	1.199478428	19.45864028	3.48E-20
Mpp6	1.197461774	3.279690937	0.000525
Clca5	1.192674185	2.563265823	0.002734
Sdk1	1.186514161	11.11996466	7.59E-12
Mcm8	1.185272383	8.612669606	2.44E-09
C1s1	1.181704936	2.891661036	0.001283
Col6a1	1.181391377	12.39279495	4.05E-13
Spag4	1.180433724	2.114720945	0.007679
Tnfaip8l1	1.170059581	13.7919319	1.61E-14
Mcm3	1.168182903	11.98819222	1.03E-12
Dut	1.16667213	24.23739098	5.79E-25
Abat	1.165996917	2.021891961	0.009508
Cdca7	1.163739363	10.37720716	4.20E-11
Pf4	1.160461121	9.508179307	3.10E-10
Dclk1	1.15950876	12.69946493	2.00E-13
Adamts3	1.158199217	4.893500232	1.28E-05
Hmgb3	1.156852788	14.26038354	5.49E-15
Blm	1.155751759	3.091798271	0.000809
Reep2	1.155604838	4.256770263	5.54E-05
Lhfpl2	1.147927462	6.128403913	7.44E-07
Lrrn4cl	1.147013991	4.184221201	6.54E-05
Prim1	1.145700976	15.25706651	5.53E-16
Itih5	1.144281163	5.077309159	8.37E-06
Brca2	1.141715722	3.259718269	0.00055
Wee1	1.140199561	16.70997661	1.95E-17
Basp1	1.138719089	7.424967951	3.76E-08
Cntrob	1.137852864	11.79510569	1.60E-12
Cthrc1	1.134193399	4.65055163	2.24E-05
Mcm6	1.132204031	19.6183966	2.41E-20
Mrgprf	1.129060383	2.365453609	0.004311
Tro	1.12742728	4.850161475	1.41E-05
Uchl1	1.125791643	10.14060764	7.23E-11
Adamtsl2	1.125471346	2.650563678	0.002236

Ncam1	1.118457147	7.062944367	8.65E-08
Rfc4	1.117949527	17.83219313	1.47E-18
Cdh2	1.117217952	14.82187641	1.51E-15
Pde10a	1.116272906	4.732891018	1.85E-05
Gm1976	1.115092721	2.982472827	0.001041
Ska2	1.10931416	12.26360684	5.45E-13
Cdh24	1.109185957	4.09305865	8.07E-05
Tmc7	1.108747108	6.424341766	3.76E-07
Ranbp17	1.107276528	2.013221692	0.0097
Zfpm2	1.10489161	8.164014484	6.85E-09
Fkbp10	1.104027823	27.44115448	3.62E-28
Serpina3i	1.103880413	2.178152272	0.006635
Pole2	1.102586908	14.75193109	1.77E-15
Mcm7	1.09815637	9.101912901	7.91E-10
Spsb1	1.097594704	14.51068575	3.09E-15
Loxl1	1.095888736	23.95211417	1.12E-24
Fabp4	1.095676157	14.92161318	1.20E-15
Raet1b	1.08826977	3.145145394	0.000716
Tgm1	1.085386571	6.027932217	9.38E-07
Zranb3	1.081027905	4.848812387	1.42E-05
Slc26a4	1.074005438	2.611531883	0.002446
Sox9	1.073550396	3.660026994	0.000219
Tpbg	1.07315415	5.136518881	7.3E-06
Pdlim4	1.070421499	2.830281846	0.001478
Pola1	1.067293351	6.654475563	2.22E-07
Prn	1.064089979	6.428627917	3.73E-07
Pkmyt1	1.059970679	5.671474258	2.13E-06
Tubb5	1.05755595	11.19325542	6.41E-12
Lingo1	1.054615754	3.218567149	0.000605
Map3k7cl	1.05289253	2.968701279	0.001075
Zfp367	1.050960318	4.837975003	1.45E-05
Hn1l	1.049547678	10.81337587	1.54E-11
Tmem26	1.034088867	2.268502551	0.005389
AI506816	1.033161497	2.537058472	0.002904
Atad5	1.030647044	5.485456752	3.27E-06
Foxd2os	1.028483307	5.126564255	7.47E-06
Bdkrb1	1.027165979	2.00083002	0.009981
Slc29a4	1.025295182	5.74813222	1.79E-06
Dok2	1.02386598	5.151613497	7.05E-06
Chil3	1.022066059	2.385933309	0.004112
Col6a3	1.021327358	5.156020251	6.98E-06
Adamts17	1.020449066	2.703326654	0.00198
Pyroxd2	1.015491029	2.507135318	0.003111
Aoc3	1.013220245	3.157364467	0.000696
Mst1r	1.011781444	2.252178507	0.005595
Cchcr1	1.010874208	6.329078989	4.69E-07
Procr	1.007286084	8.348864439	4.48E-09
Sertad4	1.007136657	3.764100528	0.000172

Rab3il1	1.004612895	28.83690771	1.46E-29
Ndc1	1.004556256	6.23932829	5.76E-07
Stmn1	1.004149061	12.98089091	1.04E-13
Ckap4	1.00148935	29.85988104	1.38E-30
Ube2s	1.001211669	9.669497399	2.14E-10
Fcgr2b	1.000251169	3.872178775	0.000134
Fabp1	-1.002648926	11.10866768	7.79E-12
Ryr2	-1.004859525	2.188314048	0.006482
Cxcr5	-1.005853332	5.108716007	7.79E-06
H2-Eb2	-1.011719912	3.165243519	0.000684
Myo5c	-1.011916021	4.913764293	1.22E-05
Pgr	-1.01330534	6.397234087	4.01E-07
Ttc25	-1.015527322	2.205887816	0.006225
Liph	-1.017550731	2.778781038	0.001664
Dlec1	-1.01968142	2.201538044	0.006287
Fam154b	-1.021048451	6.127823152	7.45E-07
Fam71f2	-1.023041019	8.739021042	1.82E-09
Ccdc37	-1.024128117	3.534007839	0.000292
Cbx7	-1.026900396	9.00218403	9.95E-10
Slc44a4	-1.02746174	2.672290512	0.002127
Rsph1	-1.027488855	3.186356331	0.000651
Klhl38	-1.027805401	2.073886854	0.008436
Lrrc48	-1.029225432	3.133313959	0.000736
Errfi1	-1.030384934	59.85818359	1.39E-60
Kcnj13	-1.031005542	3.250189155	0.000562
Nr3c2	-1.036720944	14.55040173	2.82E-15
Trp73	-1.040549482	2.784145217	0.001644
Fam92b	-1.043839364	2.096879471	0.008001
Ccdc121	-1.045270787	2.823940594	0.0015
Fank1	-1.045344174	2.355627758	0.004409
Acaa1b	-1.045533818	10.94433697	1.14E-11
Sntb1	-1.04708144	14.86810735	1.35E-15
Enkur	-1.047983614	2.945838201	0.001133
Dnali1	-1.054901945	2.97828024	0.001051
2410004P03Rik	-1.055290981	2.718711458	0.001911
Scn4a	-1.057355954	3.097170832	0.0008
Kcnk2	-1.057651834	4.675492975	2.11E-05
Cdkl4	-1.060573825	4.132171106	7.38E-05
Aox3	-1.060677256	4.126538839	7.47E-05
Armc3	-1.062604277	2.62439069	0.002375
Gm8369	-1.062942863	2.216648474	0.006072
Foxj1	-1.065611362	10.5577639	2.77E-11
Bspry	-1.068805031	5.056739559	8.78E-06
Zmynd10	-1.075397246	3.558313164	0.000276
Wdr16	-1.076343104	2.363796969	0.004327
Ccdc30	-1.079837656	4.335226332	4.62E-05
Nyx	-1.081204949	3.030063401	0.000933
Gbp10	-1.081946118	3.862456165	0.000137

Ccdc170	-1.087352683	4.560336628	2.75E-05
Wfdc8	-1.088633512	2.429122975	0.003723
Osbpl6	-1.089679961	3.842838431	0.000144
BC051019	-1.091970237	2.13866756	0.007267
Maats1	-1.09470455	5.901499603	1.25E-06
Dixdc1	-1.094744722	11.45338802	3.52E-12
Tcp11	-1.096706268	4.672952515	2.12E-05
Wdr63	-1.098881843	3.067189464	0.000857
Hc	-1.102371222	6.117328781	7.63E-07
Tspan1	-1.104667569	4.649213921	2.24E-05
Mycbpap	-1.107007925	4.533533879	2.93E-05
Tsnaxip1	-1.108532026	3.764489185	0.000172
Ankrd45	-1.108532456	2.675288301	0.002112
Srcin1	-1.115516859	3.360866607	0.000436
4930562C15Rik	-1.121389718	4.933446226	1.17E-05
Erich2	-1.121549736	4.220995052	6.01E-05
Armc2	-1.122179233	2.409370842	0.003896
Ppil6	-1.123839669	2.821264433	0.001509
Spag6	-1.127043904	6.685332799	2.06E-07
Dnah10	-1.127208556	2.656372881	0.002206
Iqub	-1.127455988	2.816380876	0.001526
Cacna1i	-1.129503863	5.523018889	3E-06
Gabrp	-1.131142717	4.006361779	9.85E-05
Scgb3a2	-1.131189154	4.414136376	3.85E-05
Nme5	-1.132203965	3.50349623	0.000314
Dynlrb2	-1.135555709	6.375497387	4.21E-07
Cyp4b1	-1.13618939	6.352458444	4.44E-07
Fmo1	-1.136226105	8.964465056	1.09E-09
8430408G22Rik	-1.137478465	19.49623228	3.19E-20
Atp6v1c2	-1.138469046	3.648351309	0.000225
4-Mar	-1.144590674	2.810240369	0.001548
Gfra1	-1.146263102	3.04444346	0.000903
Vpreb3	-1.147917957	6.881041837	1.32E-07
Ccr9	-1.148564373	2.633415273	0.002326
Ccdc108	-1.14936831	3.11976066	0.000759
Gpx2	-1.151591153	2.411659874	0.003876
Tekt1	-1.152118137	2.835737848	0.00146
1700007K13Rik	-1.152292543	9.055017358	8.81E-10
1700024G13Rik	-1.158842476	2.51894861	0.003027
Syt5	-1.160047557	2.378308783	0.004185
Wdr93	-1.165964005	2.738983703	0.001824
Pifo	-1.167017066	2.60985878	0.002456
Ubxn10	-1.170939662	3.586397213	0.000259
1700009P17Rik	-1.174472804	2.159320975	0.006929
1700028P14Rik	-1.181388434	2.096473538	0.008008
Acer2	-1.182746481	2.913029533	0.001222
Sec14l4	-1.183614614	2.845869413	0.001426
A830018L16Rik	-1.185596625	2.968263659	0.001076

Ccdc146	-1.185855001	3.465695279	0.000342
Ppargc1a	-1.187127581	4.067972556	8.55E-05
Ttc21a	-1.18824059	3.626306586	0.000236
5330417C22Rik	-1.19418296	3.498657083	0.000317
Plekhb1	-1.194442239	18.84797267	1.42E-19
Map3k19	-1.196816997	4.413731703	3.86E-05
Atp7b	-1.197092044	7.164798194	6.84E-08
Gm11346	-1.19806084	3.271471332	0.000535
Foxa1	-1.214710092	3.761220725	0.000173
Lypd2	-1.21576181	2.03931095	0.009135
Klhdc7a	-1.219638269	6.40627241	3.92E-07
Ccdc11	-1.220610534	6.317575287	4.81E-07
Gm867	-1.222207083	2.735788454	0.001837
Mapk15	-1.225657723	6.016397128	9.63E-07
Fhad1	-1.225711908	6.574385692	2.66E-07
Ces1e	-1.228013106	3.180544443	0.00066
Hmgcs2	-1.22802358	2.850771694	0.00141
Drc1	-1.23179292	5.883795847	1.31E-06
Lrguk	-1.232336144	3.216077768	0.000608
Cbr2	-1.232795429	10.02068009	9.53E-11
1110017D15Rik	-1.234005499	5.113067073	7.71E-06
4833427G06Rik	-1.2374444	3.228014569	0.000592
Ces2b	-1.245334719	7.398233804	4.00E-08
Kctd8	-1.246835966	2.077279983	0.00837
Tekt4	-1.24861356	2.290534164	0.005122
Fam179a	-1.251401858	8.739021042	1.82E-09
Ttll6	-1.256561438	3.778467906	0.000167
Muc5b	-1.256620231	2.949051503	0.001124
Adam28	-1.259103655	3.535716838	0.000291
Nek5	-1.261042666	2.494080469	0.003206
Nrn1	-1.262955973	3.998798168	0.0001
Adra1a	-1.263667359	7.738649229	1.83E-08
Daw1	-1.266861441	2.909594974	0.001231
Clic6	-1.267126216	6.434635385	3.68E-07
Rgs22	-1.267146325	4.454655392	3.51E-05
Fam183b	-1.271150963	5.26865059	5.39E-06
Tcte1	-1.273902569	2.633376904	0.002326
Ccdc153	-1.273928858	5.115903408	7.66E-06
Ccdc40	-1.275542439	2.912122705	0.001224
Faim2	-1.276225159	12.46074389	3.46E-13
Zfp474	-1.2791258	2.358374047	0.004382
Ak7	-1.28386717	4.383025115	4.14E-05
Ccdc113	-1.286608033	4.037775054	9.17E-05
Mdh1b	-1.288106315	2.8542115	0.001399
Gas2l2	-1.288616607	2.650563678	0.002236
Dnah6	-1.289478421	5.60355875	2.49E-06
Ccdc81	-1.290161168	2.314616675	0.004846
Cacna2d4	-1.294002217	2.276124513	0.005295

Kif27	-1.294175013	12.23927245	5.76E-13
Elmod1	-1.294409999	3.182972862	0.000656
4930451C15Rik	-1.298285508	2.037126255	0.009181
Iyd	-1.301789875	2.790945934	0.001618
Stk33	-1.304723588	5.706304183	1.97E-06
Acoxl	-1.306662081	5.67615638	2.11E-06
Cfap44	-1.313038853	3.799498132	0.000159
Ttc18	-1.314960182	3.189965076	0.000646
Snhg11	-1.31505066	9.315813359	4.83E-10
Fam216b	-1.315961248	6.811546156	1.54E-07
Cdhr3	-1.320456067	3.892557522	0.000128
Glb1l3	-1.328802245	13.93798814	1.15E-14
Cldn8	-1.328803535	2.100885054	0.007927
Dnah9	-1.335830984	2.668768058	0.002144
1700001C02Rik	-1.338267379	2.88541292	0.001302
Lrrc43	-1.343729691	2.992293412	0.001018
Lrrc6	-1.345126593	4.994180738	1.01E-05
Nhlrc4	-1.345810984	2.168862963	0.006779
Edn1	-1.348762621	2.244417053	0.005696
1700003M02Rik	-1.360176433	4.694393991	2.02E-05
Rsph4a	-1.368473402	5.834250757	1.46E-06
Kcnmb2	-1.369343319	5.266234967	5.42E-06
Spata18	-1.36938689	3.159017026	0.000693
1810010H24Rik	-1.378443249	5.37190118	4.25E-06
Reg3g	-1.378597671	2.103024438	0.007888
Aldh3a1	-1.379422782	3.401013597	0.000397
Dnah5	-1.383561211	7.630180062	2.34E-08
Agr3	-1.403911633	3.916235439	0.000121
Ccdc147	-1.410256935	2.324055686	0.004742
Scgb1a1	-1.414733893	5.602032517	2.5E-06
Atp2c2	-1.417587849	3.638479119	0.00023
Fsip1	-1.424272012	2.919889219	0.001203
Tmem212	-1.439509449	3.191375622	0.000644
1600029I14Rik	-1.441309964	3.195692266	0.000637
Fam47e	-1.445700556	3.415903472	0.000384
Iqca	-1.449341828	3.222769853	0.000599
1700026D08Rik	-1.452851263	5.265459709	5.43E-06
Ttc29	-1.46079333	6.316838646	4.82E-07
Klhl14	-1.462942871	2.50844366	0.003101
Spag16	-1.464551441	7.091214154	8.11E-08
Nek11	-1.465272152	3.866850655	0.000136
Pla2g1b	-1.465576718	4.283117481	5.21E-05
Cyp4a32	-1.467743895	10.91278231	1.22E-11
BC048546	-1.468885741	4.111033791	7.74E-05
Klf15	-1.469441403	2.457985348	0.003483
Efcab6	-1.474092303	3.225329613	0.000595
Itpka	-1.479035716	4.608130743	2.47E-05
Ccdc33	-1.484876785	2.144908701	0.007163

Lrrc26	-1.486746306	4.55658357	2.78E-05
C330013F16Rik	-1.503145458	2.30368213	0.00497
Colq	-1.511408864	54.09128211	8.10E-55
Igfbp3	-1.531685449	19.19363055	6.40E-20
Ldlrad1	-1.538661613	4.697496388	2.01E-05
Cyp2f2	-1.539234069	7.944945923	1.14E-08
Scgb3a1	-1.540804393	3.090306049	0.000812
Eno4	-1.549349574	6.535239777	2.92E-07
Sntn	-1.550820641	5.355023722	4.42E-06
Arhgef38	-1.566576877	5.232103872	5.86E-06
Lrriq1	-1.567394045	2.121876216	0.007553
D430036J16Rik	-1.578765311	4.394823237	4.03E-05
Kndc1	-1.595446323	4.700142601	1.99E-05
Slc10a5	-1.615084954	2.506480953	0.003115
Fgfbp1	-1.619967561	11.80567094	1.56E-12
Ptpru	-1.620879628	8.515351688	3.05E-09
Bpifb1	-1.626323777	2.566316617	0.002714
Krt15	-1.659132237	4.751844803	1.77E-05
Fmo3	-1.667349208	6.188360609	6.48E-07
Pon1	-1.692996768	11.24937882	5.63E-12
Slc16a5	-1.695760173	5.140976835	7.23E-06
Aqp4	-1.729897321	7.702227298	1.99E-08
Dcdc2a	-1.74892799	3.729215095	0.000187
Cyp2a5	-1.769331116	15.81024252	1.55E-16
Ttc34	-1.785839167	5.140976835	7.23E-06
Fabp12	-1.818058432	4.474994082	3.35E-05
Gm12695	-1.894375107	2.915523023	0.001215
Cyp1a1	-1.912316596	41.6129434	2.44E-42
Sult1d1	-1.936276022	5.121754858	7.56E-06
Esm1	-1.983727039	36.60265857	2.50E-37
Hes2	-2.073310536	4.782409677	1.65E-05
D130043K22Rik	-2.165228004	4.517029735	3.04E-05
Il22ra2	-2.360486815	2.424443103	0.003763
Krt5	-2.376069607	2.691658478	0.002034
9530026P05Rik	-2.411992464	11.73140027	1.86E-12
Bpifa1	-2.427392823	16.84955128	1.41E-17
Rag1	-2.481411597	5.163105486	6.87E-06
Gm609	-2.601512933	2.05530625	0.008804
Lrat	-2.810287146	11.32893946	4.69E-12
Cyp26b1	-6.424675798	8.453826389	3.52E-09
