# Supplementary material for: Inhibition of angiogenesis and regenerative lung growth in Lepob/ob mice through adiponectin-VEGF/VEGFR2 signaling
Source: Front Cardiovasc Med. 2024 Oct 16;11:1491971. doi: 10.3389/fcvm.2024.1491971 (PMC11521822; doi:10.3389/fcvm.2024.1491971)
Supplement: Supplementary file 5 [file Datasheet3.pdf]

Cell Cycle	2.58685E-52	4.98131113	7.59757E-49	ERCC6L, ZWILCH, DSCC1, GMNN, CCNF, DIXDC1, BUB1B, MKI67, SMC2, CDC20, CHEK2, CHEK1, NUSAP1, RCC1, NEK2, OIP5, KNTC1, FBXO5, BORA, LIG1, TRP73, ESCO2, CDC25C, KNSTRN, MELK, CCNE2, CCNE1, FANCD2, FAM64A, KIF20B, CDCA2, CDCA3, CDCA5, CDCA8, PKMYT1, SKA3, PMF1, NCAPH, SKA1, SKA2, CCNB2, DSN1, CCNB1, BRINP1, RACGAP1, CLSPN, ECT2, FAM83D, CDT1, UBE2C, DDIAS, PLK1, CDC7, CDC6, NDC80, ANLN, TPX2, KIF18B, UBE2S, CDK1, CNTROB, MCM7, MCM8, NCAPG2, BRCA1, KIF11, CHTF18, FOXM1, BRCA2, CKS1B, SMPD3, CHAF1B, CHAF1A, SGOL1, MIS18BP1, GSG2, NUF2, MYBL2, SPDL1, DLGAP5, CEP55, HELLS, H2AFX, CKAP2, KIF23, MASTL, CCSAP, CIT, CCNA2, ASPM, CCNA1, DBF4, PSRC1, INCENP, MCM3, CKS2, BIRC5, GAS1, MCM5, KIF2C, MCM6, MCM2, UHRF1, NSL1, AURKB, AURKA, CDC45, E2F1, BUB1, E2F7, E2F8, CENPW, CDKN2C, SPAG5, TICRR, CENPE, WEE1, APITD1, PRC1, NCAPD2, SPC24, MAD2L1, SPC25, NEK11, CDKN3
DNA Replication	2.07287E-20	7.161424469	1.522E-17	PIF1, FEN1, BLM, 2810417H13RIK, MCM7, MCM8, PRIM1, DSCC1, MCM10, **BRCA1**, CHTF18, CHAF1B, CDC45, CHAF1A, ORC1, POLE, GINS1, POLQ, GINS2, CDT1, RMI2, RRM1, RRM2, RFC4, LIG1, CDC6, ADRA2A, TICRR, POLA1, DBF4, POLE2, MCM3, MCM5, MCM6, DTL, MCM2
Chromosome Segregation	7.07731E-18	7.972784358	4.15721E-15	TOP2A, CDCA2, **BRCA1**, KIF11, SKA3, PMF1, NSL1, SKA1, SKA2, DSN1, SGOL1, RCC1, NEK2, OIP5, BUB1, CENPW, SPAG5, ESCO2, KNSTRN, NDC80, CENPE, CENPF, ESPL1, CENPH, INCENP, BIRC5, KIF2C, CENPN, SPC25
Cellular Response to DNA Damage Stimulus	1.16369E-10	2.854623365	4.8825E-08	TOP2A, FEN1, MCM7, MCM8, MCM10, BRCA1, FOXM1, BRCA2, PIDD1, CHAF1B, CHAF1A, EXO1, CHEK2, CHEK1, POLE, GEN1, LIG1, H2AFX, TRP73, MASTL, MMS22L, NEIL3, RAD51C, FANCD2, SPATA18, DTL, PIF1, 2810417H13RIK, BLM, PARPBP, UHRF1, UNG, RAD54B, RAD51AP1, BRIP1, RAD54L, CLSPN, E2F7, ZRANB3, BARD1, POLQ, EGLN3, ATAD5, DDIAS, TICRR, RAD51, APITD1, UBE2T, CDKN3
DNA Repair	3.26326E-08	2.846928962	9.58418E-06	PIF1, FEN1, BLM, 2810417H13RIK, ANKLE1, PARPBP, MCM8, UHRF1, BRCA1, FOXM1, BRCA2, UNG, RAD51AP1, BRIP1, CHAF1B, CHAF1A, EXO1, CHEK2, CHEK1, RAD54L, CLSPN, POLE, ZRANB3, GEN1, BARD1, POLQ, LIG1, H2AFX, KIF22, TICRR, MMS22L, NEIL3, RAD51C, RAD51, APITD1, FANCD2, UBE2T
Chemokine-Mediated Signaling Pathway	2.33183E-07	6.22826916	5.26815E-05	CCL24, CCL12, CCL11, CXCL1, CXCL3, CXCL13, CXCL5, CCL9, CCL8, CCL7, CCR9, **CCL2**, ACKR1, **PF4**
Cellular Response to Tumor Necrosis Factor	4.7248E-07	4.226325501	9.91196E-05	CCL24, CCL12, EDN1, CALCA, CCL11, **BRCA1**, ADAMTS12, COL1A1, CCL9, CCL8, FABP4, CCL7, OCSTAMP, **CCL2**, HAS2, PCK1, PPARGC1A, DBN1, ADAMTS7
Cellular Response to Interleukin-1	3.8958E-06	4.587787551	0.000673057	CCL24, CCL12, EDN1, CCL11, SAA3, FN1, ADAMTS12, CCL9, CCL8, CCL7, **CCL2**, HAS2, **SOX9**, PCK1, ADAMTS7
Double-Strand Break Repair via Homologous Recombination	1.74004E-05	4.677744169	0.002839158	GEN1, BLM, MCM8, H2AFX, TONSL, **BRCA1**, **BRCA2**, RAD54B, RAD51AP1, MMS22L, RAD51, RAD51C, RAD54L
Chemotaxis	2.41355E-05	3.5250797	0.003544302	CCL24, CCL12, CCL11, HMGB2, CXCR5, CXCL3, CXCL13, CXCL5, CCL9, CCL8, CCL7, CCR9, C3AR1, **CCL2**, CCR5, CCR3, **PF4**
Collagen Catabolic Process	3.4529E-05	8.156066757	0.004829131	ADAMTS2, MMP14, MMP13, CTSK, **MMP2**, MMP19, PRTN3, MMP10
Aging	8.62644E-05	2.828693673	0.009155845	NTRK1, CALCA, ARG1, SERPINF1, ABAT, TYMS, ADRA1A, AURKB, AGT, DCN, ALDH3A1, COL3A1, KRT14, CYP1A1, **CCL2**, NCAM1, TIMP1, **APOE**, PCK1, PPARGC1A
Cytokinesis	9.99357E-05	5.95172439	0.010027859	PRC1, INCENP, PLK1, BIRC5, KIF23, KIF20A, ECT2, **BRCA2**, CIT
Cell Activation	0.000164448	10.48637154	0.015093221	LYPD1, FN1, LYPD2, **IGF1**, TIMP1, SLURP1
Inflammatory Response	0.000190111	2.133854675	0.016919895	CCL12, TPSB2, CALCA, CCL11, CD5L, HMGB2, CXCL1, REG3G, CXCL3, CXCL13, CXCL5, CCL9, CYP26B1, CCL8, CCL7, SPP1, C3AR1, **CCL2**, BDKRB1, CCR5, CCR3, CCL24, CD163, NR1H4, TRP73, CHIL3, HC, ACKR1, CHIL4, **PF4**
Cellular Response to Retinoic Acid	0.000282918	4.140772353	0.023740834	COL1A1, CYP26A1, CYP26B1, BRINP1, TESC, SERPINF1, OSR1, TNC, **CCL2**, **SOX9**, PCK1
Response to Drug	0.000344462	2.093149876	0.025940634	COL18A1, NNMT, MCM7, HMGB2, ABAT, PTN, TYMS, ADRA1A, RAD54B, CCNB1, CDH3, LGALS1, MDK, RAD54L, NCAM1, PPARGC1A, ABCB1B, NTRK1, CBX7, ARG1, **MMP2**, TRP73, INHBA, COL1A1, ALDH3A1, RAD51, SCGB1A1, CYP1A1, CDK1
Cellular Response to Interferon-Gamma	0.000411696	3.95809122	0.029491463	CCL24, CCL9, CCL12, EDN1, CCL8, CCL11, GBP10, CCL7, WNT5A, **CCL2**, AQP4
Eosinophil Chemotaxis	0.000474593	12.23410014	0.031679074	CCL24, CCL11, CCL7, **CCL2**, CCR3
Endodermal Cell Differentiation	0.000642531	6.343607478	0.03948219	MMP14, COL11A1, **MMP2**, COL6A1, FN1, INHBA, NODAL
Male Gonad Development	0.000645266	3.058525034	0.03948219	RRM1, TESC, WNT5A, HMGB2, LRRC6, INHBA, ASPM, MMP14, BRIP1, **WT1**, SOX8, **SOX9**, WNT4, BOK
DNA Recombination	0.000661701	3.454334156	0.039502813	RAD51AP1, PIF1, BLM, RAD51, RAD51C, LIG1, EXO1, H2AFX, HMGB2, **BRCA1**, **BRCA2**, RAG1
Spermatogenesis	0.000672503	1.923789702	0.039502813	LRGUK, HMGB2, **BRCA2**, CHTF18, AK7, C330027C09RIK, CLGN, BRIP1, ADAMTS2, CCNB1, CYP26B1, RACGAP1, **E2F1**, 1110017D15RIK, SOX8, **SOX9**, TSNAXIP1, CCDC33, TCP11, RPL39L, NDC1, H2AFX, NME5, CDC25C, CIT, CCNA1, ASPM, RAD51C, **WT1**, MYCBPAP, TRIP13, SPATA18
Cell Proliferation	0.000712836	2.335600935	0.041050973	MCM7, UHRF1, MCM10, **IGF1**, MKI67, **BRCA2**, AURKB, CKS1B, POLA1, UCHL1, MELK, CKS2, CYP1A1, CDK1, TACC3, FAM83D, BUB1, TSPAN1, CFB, E2F8, BOK
Extracellular Matrix Organization	0.000764012	3.0048667	0.042591403	VIT, COL18A1, OLFML2B, LAMA1, ELN, FN1, NID2, AGT, CCDC80, COL5A3, ADAMTSL2, COL6A4, VWA1, **SOX9**
Protein Phosphorylation	0.000768588	1.741660089	0.042591403	LAMA1, BUB1B, TTK, MST1R, PKMYT1, AURKB, AURKA, CCNB1, STK33, CHEK2, GSG2, CHEK1, PBK, NEK2, EPHB2, BUB1, EPHB1, PLK4, NTRK1, CDKL4, NEK5, IGFBP3, PLK1, WNT5A, CDC7, PASK, MASTL, MAPK15, DCLK1, CIT, MAPK10, WEE1, MELK, CCNE1, MAP3K19, CDK1, BIRC5, FAM20C, ROR2, CAMK1G, NEK11
Positive Regulation of Inflammatory Response	0.000982989	3.883841313	0.053463662	CCL24, CCL9, CCL12, CCL8, CCL11, FABP4, CCL7, WNT5A, **CCL2**, CCR5
Monocyte Chemotaxis	0.001053875	4.893640054	0.055271988	CCL24, CCL9, CCL12, CALCA, CCL8, CCL11, CCL7, **CCL2**
G2 DNA Damage Checkpoint	0.001317871	6.990914363	0.065603192	PLK1, CHEK1, CLSPN, **BRCA1**, DTL, NEK11
Neutrophil Chemotaxis	0.001895059	3.546115981	0.091242432	CCL24, CCL9, CCL12, CCL8, CCL11, CCL7, SPP1, **CCL2**, CXCL1, CXCL3
Lymphocyte Chemotaxis	0.001952452	5.1902243	0.092489549	CCL24, CCL9, CCL12, CCL8, CCL11, CCL7, **CCL2**
Cellular Response to Platelet-Derived Growth Factor Stimulus	0.002036809	6.383008766	0.093470446	CCNA2, ERRFI1, HAS1, **CCL2**, HAS2, PTN
Positive Regulation of Transcription, DNA-Templated	0.002549274	1.65670106	0.111749538	MEG3, BLM, MYRF, HMGB2, **BRCA1**, FOXM1, **BRCA2**, CKS1B, MDK, CHEK2, **E2F1**, SOX8, **SOX9**, PPARGC1A, **FGF23**, WNT4, TESC, ATAD2, WNT5A, TRP73, EBF2, EBF3, **IGF1**, INHBA, GDF6, KLF15, ETV4, AGT, CCNA2, COL1A1, MLXIPL, PSRC1, **WT1**, LHX2, CKS2, NFE2L3, PGR, ROR2, ZFPM2
Response to Lipopolysaccharide	0.003005409	2.235673121	0.127925871	SPON2, EDN1, WFDC21, SPARC, PTGER3, HMGB2, FMO1, CXCL1, CXCL3, CXCL13, LOXL1, CXCL5, DCN, SCGB1A1, CYP1A1, BDKRB1, PCK1, **PF4**
Cellular Response to Hypoxia	0.003196758	2.850664109	0.132597406	FABP1, CCNA2, CCNB1, EDN1, FNDC1, **E2F1**, TWIST1, HMOX1, PTN, PCK1, PPARGC1A, KCNK2
Maternal Process Involved In Parturition	0.0032506	12.23410014	0.132597406	EDN1, CYP1A1, **CCL2**, NODAL
Response to Activity	0.003537167	3.994808207	0.142310414	NTRK1, EDN1, ADIPOQ, CDK1, **CCL2**, PTN, PCK1, PPARGC1A
ERK1 and ERK2 Cascade	0.003598882	5.646507755	0.142836703	CCL11, **IGF1**, **SOX9**, IQGAP3, CCR3, AGT
Response to Mechanical Stimulus	0.00395037	3.495457182	0.152590037	COL3A1, MMP14, CCNB1, **MMP2**, TNC, **CCL2**, BDKRB1, KCNK2, DCN
Double-Strand Break Repair	0.00395037	3.495457182	0.152590037	POLQ, LIG1, CHEK2, CDCA5, RAD54L, TRIP13, ESCO2, **BRCA1**, **BRCA2**
Immune Response	0.004000488	1.97904561	0.152590037	H2-EB2, CCL24, CCL12, CCL11, CMA1, MCPT4, CXCR5, CXCL1, PRG4, CXCL3, CXCL13, CXCL5, VPREB3, CCL9, CCL8, CCL7, CCR9, **CCL2**, ENPP3, CCR5, FCGR2B, **PF4**
Positive Regulation of Cell Migration	0.004084037	2.169594113	0.153779715	COL18A1, LRRC15, CCL24, EDN1, CEMIP, CCL11, TNFAIP6, **MMP2**, ARHGEF39, FN1, **IGF1**, ADRA2A, COL1A1, MMP14, FAM110C, C3AR1, HAS2, ROR2
Cell Chemotaxis	0.004410917	3.136948753	0.163985627	CCL9, CCL12, CCL8, SAA3, HMGB2, **CCL2**, CXCL1, CXCL13, EPHB1, CXCL5
Protein K6-Linked Ubiquitination	0.004728777	10.87475568	0.16581837	BARD1, UBE2S, UBE2T, **BRCA1**
Retinal Rod Cell Differentiation	0.004855424	24.46820027	0.16581837	SOX8, **SOX9**, PTN
Renal Vesicle Induction	0.004855424	24.46820027	0.16581837	SOX8, **SOX9**, WNT4
Negative Regulation of Neuron Apoptotic Process	0.005968007	2.293893775	0.196944243	GABRB3, NTRK1, CCL12, KIF14, TRP73, FAIM2, AGT, CHL1, MDK, HMOX1, **CCL2**, BIRC5, **APOE**, PPARGC1A, BOK
Regulation of Cell Cycle	0.006057104	2.621592886	0.197663479	FOXA1, 2810417H13RIK, CCNE2, CCNE1, FIGNL1, CCNF, **E2F1**, TACC3, MASTL, SKP2, DTL, PRR11
Complement Activation, Alternative Pathway	0.006551998	9.787280108	0.202560201	CFD, HC, **CFP**, CFB
Positive Regulation of Gtpase Activity	0.006846855	2.346265779	0.207311482	NTRK1, CCL24, CCL12, CCL11, RGS16, WNT5A, ELMOD1, CCL9, CCL8, CCL7, **CCL2**, ECT2, EZH2, WNT4
Negative Regulation of Neuron Death	0.00747482	3.495457182	0.222867399	GABRB3, NTRK1, CHGA, MDK, SERPINF1, **IGF1**, **APOE**, PPARGC1A
Positive Regulation of Cell-Cell Adhesion	0.007909363	6.117050068	0.232297989	WNT5A, **CCL2**, CCR5, NODAL, TBX18
Response to Ionizing Radiation	0.008229123	3.434133371	0.239296365	RRM1, RAD51, RAD51C, KRT14, RAD54L, **BRCA1**, TICRR, RAD54B
Negative Regulation of Angiogenesis	0.00887432	3.058525034	0.253047345	MEG3, SPARC, SERPINF1, **CCL2**, PTN, THBS2, AGT, DCN, **PF4**
Cellular Response to Fatty Acid	0.009460302	5.825761969	0.262121766	CCNB1, EDN1, **E2F1**, UCP1, NR1H4
Positive Regulation of Angiogenesis	0.010625841	2.426598374	0.291664452	CCL24, CCL11, CMA1, WNT5A, VASH2, C3AR1, CYP1B1, HMOX1, HC, **BRCA1**, CCR3, NODAL
Prostate Gland Epithelium Morphogenesis	0.01130184	8.156066757	0.301759139	FOXA1, **MMP2**, TNC, **IGF1**
DNA Damage Response, Signal Transduction By P53 Class Mediator Resulting In Transcription of P21 Class Mediator	0.01130184	8.156066757	0.301759139	CHEK2, TRP73, FOXM1, **BRCA2**
Response to Gamma Radiation	0.011646232	4.317917695	0.307346211	CCL7, FANCD2, CHEK2, TRP73, **CCL2**, **BRCA2**
Cellular Response to Transforming Growth Factor Beta Stimulus	0.011813636	3.208944298	0.307346211	COL1A1, EDN1, ARG1, WNT5A, **SOX9**, PPARGC1A, DBN1, WNT4
Cell Migration	0.011825033	2.049692169	0.307346211	**MMP2**, WNT5A, GFRA1, ADAMTS12, MMP14, DEPDC1B, CDH2, COL5A1, MDK, FSCN1, BDKRB1, CD248, FAM83D, TSPAN1, NODAL, CTHRC1
Skeletal System Development	0.012080827	2.515422458	0.31124026	COL1A1, COL3A1, VCAN, EDN1, COL1A2, COL5A2, FAM20C, ROR2, **SOX9**, HAPLN1, FBN1
Cell Differentiation	0.012575311	1.442996426	0.32116252	MEG3, PTPRU, LRGUK, TENM3, MYRF, ILDR2, TWIST1, EFNA5, DMKN, CLGN, CDC20, RACGAP1, MDK, CHL1, OCSTAMP, INSC, STMN1, NHS, AGR3, 1110017D15RIK, MYBL2, SOX9, TSNAXIP1, CCHCR1, ECT2, MYBL1, FGF23, TCP11, WNT4, NTRK1, SRRM4, TESC, OSR1, WNT5A, NME5, DLK1, TBX18, DCLK1, CIT, CCNA1, MMP19, MYCBPAP, ROR2, TRIP13, SPATA18, DBN1
Positive Regulation of Vascular Endothelial Growth Factor Production	0.015251893	5.097541723	0.36887344	C3AR1, CYP1B1, HC, **BRCA1**, NODAL
Positive Regulation of Fibroblast Proliferation	0.016408321	3.011470803	0.391085139	CCNA2, CCNB1, WNT5A, **E2F1**, FN1, **IGF1**, CDC6, AGT
Chromosome Organization	0.01839287	3.863400043	0.425353221	BLM, CENPW, CDCA8, RAD54L, **BRCA2**, SMC2
Cartilage Development	0.018582161	2.685534176	0.426373481	EDN1, MMP13, LUM, COL11A1, WNT5A, PRRX2, TIMP1, **SOX9**, TYMS
Positive Regulation of Endothelial Cell Proliferation	0.019125342	2.921576152	0.435435104	CCL24, CCL11, ARG1, WNT5A, VASH2, HMGB2, **CCL2**, CCR3
Intramembranous Ossification	0.022366674	12.23410014	0.48302149	COL1A1, CTSK, **MMP2**
Regulation of Cell Proliferation	0.024179707	1.832420285	0.510892778	EGLN3, SPARC, TNC, CDCA7, PRG4, **IGF1**, **BRCA1**, CXCL3, CXCL13, FOXM1, **BRCA2**, CXCL5, NR3C2, CHEK1, **SOX9**, **PF4**, EZH2
Inner Cell Mass Cell Proliferation	0.025488237	6.117050068	0.510892778	GINS1, CHEK1, NCAPG2, **BRCA2**
Negative Regulation of Smooth Muscle Cell Migration	0.025488237	6.117050068	0.510892778	NDRG4, **IGFBP3**, ADIPOQ, PPARGC1A
Sertoli Cell Development	0.025488237	6.117050068	0.510892778	NTRK1, **WT1**, SOX8, **SOX9**
Axon Regeneration	0.025488237	6.117050068	0.510892778	DHFR, CHL1, **APOE**, FOLR1
Negative Regulation of Inflammatory Response	0.025570731	2.531193131	0.510892778	MIR147, TNFAIP6, SERPINF1, ADIPOQ, PBK, NR1H4, **APOE**, CD276, IL22RA2
Organ Regeneration	0.026050043	3.058525034	0.516952546	CCNA2, PTPRU, NNMT, CDK1, **CCL2**, MKI67, CXCL5
Positive Regulation of Male Gonad Development	0.030475355	10.48637154	0.567542262	**WT1**, **SOX9**, ZFPM2
Glomerular Visceral Epithelial Cell Differentiation	0.030475355	10.48637154	0.567542262	BASP1, **WT1**, KLF15
Lung Development	0.030531725	2.170566153	0.567542262	FOXA1, ADAMTS2, MMP14, SPARC, ARG1, WNT5A, **IGF1**, PTN, ZFPM2, NODAL, CCDC40
DNA Damage Checkpoint	0.032480729	4.078033378	0.596224379	CHEK2, H2AFX, CHEK1, **E2F1**, CLSPN
Positive Regulation of Epithelial Cell Proliferation	0.035259736	2.575600028	0.635324195	OSR1, WNT5A, TWIST1, GAS1, **IGF1**, **SOX9**, NODAL, TBX18
Proteolysis	0.036506684	1.429413762	0.642036711	CFD, TPSB2, MYRF, MCPT4, CFI, ADAMTS12, MMP23, ADAMTS4, ADAMTS16, UCHL1, ADAM28, ADAMTS2, C1S1, CAPN6, ADAMTS19, CTSK, CLCA2, CPXM1, TPSAB1, ADAMTS7, CMA1, **MMP2**, ECEL1, PRSS35, MMP10, MMP14, MMP13, ESPL1, PAPPA, ADAM12, MMP19, PRTN3, CFB, PAPPA2
Positive Regulation of Cell Proliferation	0.037356911	1.44461699	0.653078864	SHC4, COL18A1, TNC, KIF14, PTN, FOXM1, CDC20, ESM1, HAS2, **SOX9**, TIMP1, FGFBP1, EDN1, WNT5A, CLEC11A, FN1, CDC7, IGF1, AGT, FABP1, ACER2, ALDH3A1, MLXIPL, CENPF, FABP4, PRC1, GAS1, PGR, CD248, KIF20B, FOLR2, NODAL
Astrocyte Cell Migration	0.039550273	9.175575101	0.668796759	CCL12, MMP14, **CCL2**
Heart Development	0.039676611	1.687462088	0.668796759	EDN1, SPARC, DNAH5, **MMP2**, OSR1, DAW1, FOXJ1, PTN, CENPF, VCAN, COL3A1, MMP13, **WT1**, **SOX9**, ZFPM2, DRC1, NODAL, FBN1
Positive Regulation of Mitotic Cell Cycle	0.040002162	3.823156292	0.668796759	FOXA1, CCNB1, CDK1, BIRC5, **BRCA2**
Oocyte Maturation	0.040305423	5.151200057	0.668796759	CCNB1, FBXO5, TRIP13, **BRCA2**
Osteoblast Differentiation	0.041634336	2.165327458	0.686966549	COL1A1, VCAN, FIGNL1, **IGFBP3**, COL6A1, TNC, SPP1, TWIST1, SOX8, **IGF1**
Ossification	0.042407478	2.293893775	0.695814314	MMP14, SPARC, COL11A1, COL5A2, ALOX15, SPP1, TWIST1, **SOX9**, PTN
Positive Regulation of Smooth Muscle Cell Proliferation	0.044674869	2.446820027	0.723604252	EDN1, ORC1, C3AR1, HMOX1, **IGF1**, SKP2, PPARGC1A, WISP1
Positive Regulation of ERK1 and ERK2 Cascade	0.044840304	1.82210002	0.723604252	NTRK1, CCL24, NDRG4, CCL12, CCL11, ALOX15, TREM2, ADRA1A, CCL9, CCL8, CCL7, **CCL2**, **FGF23**, NODAL
Oligodendrocyte Differentiation	0.048430684	3.598264746	0.757184176	CDKN2C, MYRF, SOX8, **SOX9**, BOK
Bone Trabecula Formation	0.049499272	8.156066757	0.757184176	FBN2, COL1A1, **MMP2**
Negative Regulation of Histone Acetylation	0.049499272	8.156066757	0.757184176	MSX3, TWIST1, **BRCA1**
Protein Complex Assembly	0.052066615	2.59511215	0.779943508	CCNB1, GMNN, CDK1, **SOX9**, **PF4**, CLGN, ADD2
Response to X-Ray	0.052116053	4.660609575	0.779943508	BLM, RAD51, TRP73, **BRCA2**
Positive Regulation of Leukocyte Migration	0.058577083	4.448763686	0.860204467	AOC3, CCL12, **CCL2**, BDKRB1
Cellular Response to Vitamin D	0.06023609	7.340460081	0.867222537	TNC, PTN, **FGF23**
Nucleosome Assembly	0.062454544	2.117440408	0.886207519	CHAF1B, CHAF1A, H1FX, PADI4, **SOX9**, CENPA, HIST1H1B, MCM2, ASF1B
Positive Regulation of Mesenchymal Cell Proliferation	0.062761717	3.30651355	0.886207519	WNT5A, PRRX2, GAS1, **SOX9**, TBX18
Cochlea Morphogenesis	0.065394973	4.255339178	0.914595401	WNT5A, **SOX9**, TBX18, CTHRC1
Negative Regulation of Smooth Muscle Cell Proliferation	0.067981582	3.219500036	0.941801442	NDRG4, **IGFBP3**, ADIPOQ, HMOX1, PPARGC1A
Protein Kinase B Signaling	0.067981582	3.219500036	0.941801442	LINGO1, CCL12, **CCL2**, **IGF1**, **SOX9**
Intrinsic Apoptotic Signaling Pathway In Response to DNA Damage	0.068556158	2.718688919	0.945302518	CHEK2, **E2F1**, HMOX1, **BRCA1**, **BRCA2**, BOK
Cellular Response to Glucose Stimulus	0.069459154	2.412357773	0.949910256	MLXIPL, LGALS1, CMA1, SERPINF1, AQP4, **IGF1**, PPARGC1A
Positive Regulation of Insulin-Like Growth Factor Receptor Signaling Pathway	0.071680036	6.673145528	0.949910256	CDH3, **IGFBP3**, **IGF1**
Telomere Maintenance via Recombination	0.071680036	6.673145528	0.949910256	RAD51, RAD51C, **BRCA2**
Cell Fate Commitment	0.073295304	2.378852804	0.949910256	WNT5A, GAS1, SOX8, ROR2, **SOX9**, NODAL, WNT4
Ureter Urothelium Development	0.079964515	24.46820027	0.979653102	OSR1, **SOX9**
Mitotic Recombination-Dependent Replication Fork Processing	0.079964515	24.46820027	0.979653102	RAD51, **BRCA2**
Posterior Mesonephric Tubule Development	0.079964515	24.46820027	0.979653102	**WT1**, OSR1
Mesonephros Development	0.083755675	6.117050068	0.979653102	**WT1**, OSR1, WNT4
Positive Regulation of Cell Growth Involved In Cardiac Muscle Cell Development	0.083755675	6.117050068	0.979653102	EDN1, **IGF1**, WISP1
Lung Epithelial Cell Differentiation	0.083755675	6.117050068	0.979653102	FOXA1, INSC, **SOX9**
Nervous System Development	0.093627566	1.427852536	0.979653102	NTRK1, SRRM4, NRN1, D130043K22RIK, CRMP1, GFRA1, **IGF1**, EFNA5, DCLK1, CIT, CDC20, DCDC2A, LHX2, CHL1, DPF1, INSC, STMN1, ECT2, EPHB2, EPHB1, DBN1, NODAL
Response to Hypoxia	0.094858812	1.65670106	0.979653102	RYR2, EDN1, EGLN3, **MMP2**, ADIPOQ, ABAT, LOXL2, ALDH3A1, MMP14, CYP1A1, TACC3, HMOX1, **CCL2**
Fatty Acid Homeostasis	0.096392539	5.646507755	0.979653102	MLXIPL, NR1H4, **APOE**
