# Supplementary material for: Inhibition of angiogenesis and regenerative lung growth in Lepob/ob mice through adiponectin-VEGF/VEGFR2 signaling
Source: Front Cardiovasc Med. 2024 Oct 16;11:1491971. doi: 10.3389/fcvm.2024.1491971 (PMC11521822; doi:10.3389/fcvm.2024.1491971)
Supplement: Supplementary file 8 [file Datasheet6.pdf]

Cell Cycle	2.58685E-52	4.98131113	ERCC6L, ZWILCH, DSCC1, GMNN, CCNF, DIXDC1, BUB1B, MKI67, SMC2, CDC20, CHEK2, CHEK1, NUSAP1, RCC1, NEK2, OIP5, KNTC1, FBXO5, BORA, LIG1, TRP73, ESCO2, CDC25C, KNSTRN, MELK, CCNE2, CCNE1, FANCD2, FAM64A, KIF20B, CDCA2, CDCA3, CDCA5, CDCA8, PKMYT1, SKA3, PMF1, NCAPH, SKA1, SKA2, CCNB2, DSN1, CCNB1, BRINP1, RACGAP1, CLSPN, ECT2, FAM83D, CDT1, UBE2C, DDIAS, PLK1, CDC7, CDC6, NDC80, ANLN, TPX2, KIF18B, UBE2S, CDK1, CNTROB, MCM7, MCM8, NCAPG2, BRCA1, KIF11, CHTF18, FOXM1, BRCA2, CKS1B, SMPD3, CHAF1B, CHAF1A, SGOL1, MIS18BP1, GSG2, NUF2, MYBL2, SPDL1, DLGAP5, CEP55, HELLS, H2AFX, CKAP2, KIF23, MASTL, CCSAP, CIT, CCNA2, ASPM, CCNA1, DBF4, PSRC1, INCENP, MCM3, CKS2, BIRC5, GAS1, MCM5, KIF2C, MCM6, MCM2, UHRF1, NSL1, AURKB, AURKA, CDC45, E2F1, BUB1, E2F7, E2F8, CENPW, CDKN2C, SPAG5, TICRR, CENPE, WEE1, APITD1, PRC1, NCAPD2, SPC24, MAD2L1, SPC25, NEK11, CDKN3
Cell Division	9.92865E-41	5.691800598	ERCC6L, ZWILCH, NCAPG2, CCNF, KIF14, BUB1B, KIF11, SMC2, CKS1B, CDC20, SGOL1, MIS18BP1, CHEK2, NUF2, NUSAP1, RCC1, NEK2, OIP5, KNTC1, FBXO5, SPDL1, CEP55, HELLS, BORA, LIG1, KIFC5B, KIF23, MASTL, CDC25C, CCSAP, KNSTRN, CIT, CCNA2, CCNA1, ASPM, PSRC1, CCNE2, KIFC1, CCNE1, INCENP, CKS2, BIRC5, FAM64A, MCM5, KIF2C, KIF20B, CDCA2, CDCA3, CDCA5, CDCA8, SKA3, PMF1, NSL1, AURKB, SKA1, NCAPH, AURKA, SKA2, CCNB2, CCNB1, DSN1, CDC45, RACGAP1, ECT2, FAM83D, BUB1, CENPW, SPAG5, UBE2C, PLK1, CDC7, CDC6, NDC80, ANLN, TPX2, CENPE, WEE1, APITD1, KIF18B, PRC1, UBE2S, CDK1, NCAPD2, CNTROB, SPC24, SPC25, MAD2L1
Mitotic Nuclear Division	6.98062E-40	6.624963972	ERCC6L, ZWILCH, NCAPG2, CCNF, BUB1B, KIF11, SMC2, CDC20, SGOL1, TUBB3, MIS18BP1, CHEK2, NUF2, NUSAP1, RCC1, NEK2, OIP5, KNTC1, FBXO5, SPDL1, CEP55, HELLS, BORA, KIF23, MASTL, CDC25C, CCSAP, KNSTRN, CIT, CCNA2, CCNA1, ASPM, PSRC1, ESPL1, INCENP, BIRC5, FAM64A, KIF2C, KIF20B, CDCA2, CDCA3, CDCA5, CDCA8, SKA3, PMF1, NSL1, AURKB, SKA1, NCAPH, AURKA, SKA2, CCNB2, CCNB1, DSN1, FAM83D, BUB1, CENPW, SPAG5, UBE2C, PLK1, CDC6, NDC80, ANLN, TPX2, CENPE, WEE1, APITD1, KIF18B, CENPH, CDK1, NCAPD2, CENPN, SPC24, SPC25, MAD2L1
DNA Replication	2.07287E-20	7.161424469	PIF1, FEN1, BLM, 2810417H13RIK, MCM7, MCM8, PRIM1, DSCC1, MCM10, BRCA1, CHTF18, CHAF1B, CDC45, CHAF1A, ORC1, POLE, GINS1, POLQ, GINS2, CDT1, RMI2, RRM1, RRM2, **RFC4**, LIG1, CDC6, ADRA2A, TICRR, POLA1, DBF4, POLE2, MCM3, MCM5, MCM6, DTL, MCM2
Chromosome Segregation	7.07731E-18	7.972784358	TOP2A, CDCA2, BRCA1, KIF11, SKA3, PMF1, NSL1, SKA1, SKA2, DSN1, SGOL1, RCC1, NEK2, OIP5, BUB1, CENPW, SPAG5, ESCO2, KNSTRN, NDC80, CENPE, CENPF, ESPL1, CENPH, INCENP, **BIRC5**, KIF2C, CENPN, SPC25
Cellular Response to DNA Damage Stimulus	1.16369E-10	2.854623365	TOP2A, FEN1, MCM7, MCM8, MCM10, BRCA1, FOXM1, BRCA2, PIDD1, CHAF1B, CHAF1A, EXO1, CHEK2, CHEK1, POLE, GEN1, LIG1, H2AFX, TRP73, MASTL, MMS22L, NEIL3, RAD51C, FANCD2, SPATA18, DTL, PIF1, 2810417H13RIK, BLM, PARPBP, UHRF1, UNG, RAD54B, RAD51AP1, BRIP1, RAD54L, CLSPN, E2F7, ZRANB3, BARD1, POLQ, EGLN3, ATAD5, DDIAS, TICRR, RAD51, APITD1, UBE2T, CDKN3
DNA Replication Initiation	7.46665E-10	12.23410014	POLA1, CDC45, **CCNE2**, MCM7, CCNE1, MCM3, CDC7, MCM5, MCM10, MCM6, CDC6, MCM2
DNA Repair	3.26326E-08	2.846928962	PIF1, FEN1, BLM, 2810417H13RIK, ANKLE1, PARPBP, MCM8, UHRF1, BRCA1, FOXM1, BRCA2, UNG, RAD51AP1, BRIP1, CHAF1B, CHAF1A, EXO1, CHEK2, CHEK1, RAD54L, CLSPN, POLE, ZRANB3, GEN1, BARD1, POLQ, LIG1, H2AFX, KIF22, TICRR, MMS22L, NEIL3, RAD51C, RAD51, APITD1, FANCD2, UBE2T
Chemokine-Mediated Signaling Pathway	2.33183E-07	6.22826916	CCL24, CCL12, **CCL11**, **CXCL1**, **CXCL3**, CXCL13, CXCL5, CCL9, CCL8, CCL7, CCR9, **CCL2**, ACKR1, PF4
Cellular Response to Tumor Necrosis Factor	4.7248E-07	4.226325501	CCL24, CCL12, **EDN1**, CALCA, **CCL11**, BRCA1, ADAMTS12, COL1A1, CCL9, CCL8, **FABP4**, CCL7, OCSTAMP, **CCL2**, HAS2, **PCK1**, **PPARGC1A**, DBN1, ADAMTS7
Cell Adhesion	6.56795E-07	2.270245386	PTPRU, SPON2, COL18A1, COL15A1, TENM3, COL16A1, TNFAIP6, LAMA1, TROAP, TNC, THBS2, NID2, WISP1, HAPLN1, CDH3, FLRT2, CDH2, CHL1, HAS1, SPP1, CLCA2, HAS2, CYP1B1, CDH24, NRCAM, NCAM1, EPHB1, AOC3, CADM4, MYBPC2, FN1, MFAP4, SDK1, VCAN, NFASC, COL5A1, COL6A2, CDHR1, COL6A1, ADAM12, COL6A4, CERCAM, CDHR3, COL6A6, COL6A5
Cellular Response to Interleukin-1	3.8958E-06	4.587787551	CCL24, CCL12, **EDN1**, **CCL11**, SAA3, FN1, ADAMTS12, CCL9, CCL8, CCL7, **CCL2**, HAS2, SOX9, **PCK1**, ADAMTS7
Collagen Fibril Organization	2.10752E-05	6.273897505	COL1A1, ADAMTS2, **COL3A1**, COL1A2, COL5A1, LUM, COL11A1, COL5A2, CYP1B1, LOXL2
Chemotaxis	2.41355E-05	3.5250797	CCL24, CCL12, **CCL11**, HMGB2, CXCR5, **CXCL3**, CXCL13, CXCL5, CCL9, CCL8, CCL7, CCR9, C3AR1, **CCL2**, **CCR5**, CCR3, PF4
Collagen Catabolic Process	3.4529E-05	8.156066757	ADAMTS2, MMP14, **MMP13**, CTSK, **MMP2**, MMP19, PRTN3, MMP10
G1/S Transition of Mitotic Cell Cycle	3.68705E-05	4.735780698	TCF19, CDKN2C, **CCNE2**, CCNE1, CDCA5, RCC1, RHOU, **INHBA**, SKP2, IQGAP3, POLE, **CDKN3**
G2/M Transition of Mitotic Cell Cycle	4.15239E-05	6.673145528	CHEK2, PLK1, CHEK1, **BIRC5**, MASTL, CDC25C, SKP2, FOXM1, CIT
Mitotic Spindle Assembly	6.54823E-05	6.291822927	CDC20, TPX2, KIFC1, CHEK2, KIFC5B, **BIRC5**, NEK2, MYBL2, KIF11
Protein Heterotrimerization	7.78534E-05	9.0146001	COL1A1, COL1A2, COL6A2, C1QTNF6, COL6A1, **ADIPOQ**, COL6A4
Aging	8.62644E-05	2.828693673	NTRK1, CALCA, **ARG1**, **SERPINF1**, ABAT, TYMS, ADRA1A, AURKB, **AGT**, **DCN**, ALDH3A1, **COL3A1**, KRT14, CYP1A1, **CCL2**, NCAM1, **TIMP1**, **APOE**, **PCK1**, **PPARGC1A**
Cytokinesis	9.99357E-05	5.95172439	PRC1, INCENP, PLK1, **BIRC5**, KIF23, KIF20A, ECT2, BRCA2, CIT
Cell Activation	0.000164448	10.48637154	LYPD1, **FN1**, LYPD2, **IGF1**, **TIMP1**, SLURP1
Inflammatory Response	0.000190111	2.133854675	CCL12, TPSB2, CALCA, **CCL11**, CD5L, HMGB2, **CXCL1**, REG3G, **CXCL3**, CXCL13, CXCL5, CCL9, CYP26B1, CCL8, CCL7, **SPP1**, C3AR1, **CCL2**, BDKRB1, **CCR5**, CCR3, CCL24, **CD163**, NR1H4, TRP73, CHIL3, HC, ACKR1, CHIL4, PF4
Positive Regulation of Protein Kinase C Activity	0.000262631	24.46820027	CEMIP, **WNT5A**, ROR2, **AGT**
Cellular Response to Retinoic Acid	0.000282918	4.140772353	COL1A1, CYP26A1, CYP26B1, BRINP1, TESC, **SERPINF1**, OSR1, TNC, **CCL2**, SOX9, **PCK1**
Response to Drug	0.000344462	2.093149876	COL18A1, NNMT, MCM7, HMGB2, ABAT, PTN, TYMS, ADRA1A, RAD54B, CCNB1, CDH3, LGALS1, MDK, RAD54L, NCAM1, **PPARGC1A**, ABCB1B, NTRK1, CBX7, **ARG1**, **MMP2**, TRP73, **INHBA**, COL1A1, ALDH3A1, RAD51, SCGB1A1, CYP1A1, CDK1
Cellular Response to Interferon-Gamma	0.000411696	3.95809122	CCL24, CCL9, CCL12, **EDN1**, CCL8, **CCL11**, GBP10, CCL7, **WNT5A**, **CCL2**, AQP4
Eosinophil Chemotaxis	0.000474593	12.23410014	CCL24, **CCL11**, CCL7, **CCL2**, CCR3
Endodermal Cell Differentiation	0.000642531	6.343607478	MMP14, COL11A1, **MMP2**, COL6A1, **FN1**, **INHBA**, NODAL
Male Gonad Development	0.000645266	3.058525034	RRM1, TESC, **WNT5A**, HMGB2, LRRC6, **INHBA**, ASPM, MMP14, BRIP1, WT1, SOX8, SOX9, WNT4, BOK
Spermatogenesis	0.000672503	1.923789702	LRGUK, HMGB2, BRCA2, CHTF18, AK7, C330027C09RIK, CLGN, BRIP1, ADAMTS2, CCNB1, CYP26B1, RACGAP1, **E2F1**, 1110017D15RIK, SOX8, SOX9, TSNAXIP1, CCDC33, TCP11, RPL39L, NDC1, H2AFX, NME5, CDC25C, CIT, CCNA1, ASPM, RAD51C, WT1, MYCBPAP, TRIP13, SPATA18
Cell Proliferation	0.000712836	2.335600935	MCM7, **UHRF1**, MCM10, **IGF1**, MKI67, BRCA2, AURKB, CKS1B, POLA1, UCHL1, MELK, CKS2, CYP1A1, CDK1, TACC3, FAM83D, BUB1, TSPAN1, **CFB**, E2F8, BOK
Extracellular Matrix Organization	0.000764012	3.0048667	VIT, COL18A1, OLFML2B, LAMA1, ELN, **FN1**, NID2, **AGT**, CCDC80, COL5A3, ADAMTSL2, COL6A4, VWA1, SOX9
Protein Phosphorylation	0.000768588	1.741660089	LAMA1, BUB1B, TTK, MST1R, PKMYT1, AURKB, AURKA, CCNB1, STK33, CHEK2, GSG2, CHEK1, PBK, NEK2, EPHB2, BUB1, EPHB1, PLK4, NTRK1, CDKL4, NEK5, IGFBP3, PLK1, WNT5A, CDC7, PASK, MASTL, MAPK15, DCLK1, CIT, MAPK10, WEE1, MELK, CCNE1, MAP3K19, CDK1, BIRC5, FAM20C, ROR2, CAMK1G, NEK11
Positive Regulation of Inflammatory Response	0.000982989	3.883841313	CCL24, CCL9, CCL12, CCL8, **CCL11**, **FABP4**, CCL7, **WNT5A**, **CCL2**, **CCR5**
Monocyte Chemotaxis	0.001053875	4.893640054	CCL24, CCL9, CCL12, CALCA, CCL8, **CCL11**, CCL7, **CCL2**
Collagen Biosynthetic Process	0.001234537	16.31213351	COL1A1, ADAMTS3, COL5A1, **ARG1**
Neutrophil Chemotaxis	0.001895059	3.546115981	CCL24, CCL9, CCL12, CCL8, **CCL11**, CCL7, **SPP1**, **CCL2**, **CXCL1**, **CXCL3**
Lymphocyte Chemotaxis	0.001952452	5.1902243	CCL24, CCL9, CCL12, CCL8, **CCL11**, CCL7, **CCL2**
Cellular Response to Platelet-Derived Growth Factor Stimulus	0.002036809	6.383008766	CCNA2, ERRFI1, HAS1, **CCL2**, HAS2, PTN
Positive Regulation of Transcription, DNA-Templated	0.002549274	1.65670106	MEG3, BLM, MYRF, HMGB2, BRCA1, FOXM1, BRCA2, CKS1B, MDK, CHEK2, **E2F1**, SOX8, SOX9, **PPARGC1A**, **FGF23**, WNT4, TESC, ATAD2, **WNT5A**, TRP73, EBF2, EBF3, **IGF1**, **INHBA**, GDF6, KLF15, ETV4, **AGT**, CCNA2, COL1A1, MLXIPL, PSRC1, WT1, LHX2, CKS2, NFE2L3, PGR, ROR2, ZFPM2
Cilium Movement	0.002670239	4.893640054	RSPH4A, **DNAH10**, DNAH5, DNAH6, LRRC6, DNALI1, CCDC40
Response to Lipopolysaccharide	0.003005409	2.235673121	SPON2, **EDN1**, WFDC21, SPARC, PTGER3, HMGB2, FMO1, **CXCL1**, **CXCL3**, CXCL13, LOXL1, CXCL5, **DCN**, SCGB1A1, CYP1A1, BDKRB1, **PCK1**, PF4
Cellular Response to Hypoxia	0.003196758	2.850664109	FABP1, CCNA2, CCNB1, **EDN1**, FNDC1, **E2F1**, TWIST1, **HMOX1**, PTN, **PCK1**, **PPARGC1A**, KCNK2
Maternal Process Involved In Parturition	0.0032506	12.23410014	**EDN1**, CYP1A1, **CCL2**, NODAL
Response to Activity	0.003537167	3.994808207	NTRK1, **EDN1**, **ADIPOQ**, CDK1, **CCL2**, PTN, **PCK1**, **PPARGC1A**
ERK1 and ERK2 Cascade	0.003598882	5.646507755	**CCL11**, **IGF1**, SOX9, IQGAP3, CCR3, **AGT**
Response to Mechanical Stimulus	0.00395037	3.495457182	**COL3A1**, MMP14, CCNB1, **MMP2**, TNC, **CCL2**, BDKRB1, KCNK2, **DCN**
Immune Response	0.004000488	1.97904561	H2-EB2, CCL24, CCL12, **CCL11**, CMA1, MCPT4, CXCR5, **CXCL1**, PRG4, **CXCL3**, CXCL13, CXCL5, VPREB3, CCL9, CCL8, CCL7, CCR9, **CCL2**, **ENPP3**, **CCR5**, FCGR2B, PF4
Positive Regulation of Cell Migration	0.004084037	2.169594113	COL18A1, LRRC15, CCL24, **EDN1**, CEMIP, **CCL11**, TNFAIP6, **MMP2**, ARHGEF39, **FN1**, **IGF1**, ADRA2A, COL1A1, MMP14, FAM110C, C3AR1, HAS2, ROR2

Categories

Adhesion/Migration

Cell Cycle/Apoptosis

Inflammatory/Immune Response

Cell Signaling
