# Supplementary material for: Inhibition of angiogenesis and regenerative lung growth in Lepob/ob mice through adiponectin-VEGF/VEGFR2 signaling
Source: Front Cardiovasc Med. 2024 Oct 16;11:1491971. doi: 10.3389/fcvm.2024.1491971 (PMC11521822; doi:10.3389/fcvm.2024.1491971)
Supplement: Supplementary file 7 [file Datasheet5.pdf]

Biological Processes	PValue	Fold Enrichment	Genes
Cell Cycle	2.58685E-52	4.98131113	ERCC6L, ZWILCH, DSCC1, GMNN, CCNF, DIXDC1, BUB1B, MKI67, SMC2, CDC20, CHEK2, CHEK1, NUSAP1, RCC1, NEK2, OIP5, **KNTC1**, FBXO5, BORA, LIG1, TRP73, ESCO2, CDC25C, KNSTRN, MELK, **CCNE2**, CCNE1, FANCD2, FAM64A, KIF20B, CDCA2, CDCA3, CDCA5, CDCA8, PKMYT1, SKA3, PMF1, NCAPH, SKA1, SKA2, CCNB2, DSN1, CCNB1, BRINP1, RACGAP1, CLSPN, ECT2, FAM83D, CDT1, UBE2C, DDIAS, PLK1, CDC7, CDC6, NDC80, ANLN, TPX2, KIF18B, UBE2S, CDK1, CNTROB, MCM7, MCM8, NCAPG2, BRCA1, KIF11, CHTF18, FOXM1, BRCA2, CKS1B, SMPD3, CHAF1B, CHAF1A, SGOL1, MIS18BP1, GSG2, NUF2, MYBL2, SPDL1, DLGAP5, CEP55, HELLS, H2AFX, CKAP2, KIF23, MASTL, CCSAP, CIT, CCNA2, ASPM, CCNA1, DBF4, PSRC1, INCENP, MCM3, CKS2, **BIRC5**, GAS1, MCM5, KIF2C, MCM6, MCM2, **UHRF1**, NSL1, AURKB, AURKA, CDC45, **E2F1**, BUB1, E2F7, E2F8, CENPW, CDKN2C, SPAG5, TICRR, CENPE, WEE1, APITD1, PRC1, NCAPD2, SPC24, MAD2L1, SPC25, NEK11, **CDKN3**
Cell Division	9.92865E-41	5.691800598	ERCC6L, ZWILCH, NCAPG2, CCNF, KIF14, BUB1B, KIF11, SMC2, CKS1B, CDC20, SGOL1, MIS18BP1, CHEK2, NUF2, NUSAP1, RCC1, NEK2, OIP5, **KNTC1**, FBXO5, SPDL1, CEP55, HELLS, BORA, LIG1, KIFC5B, KIF23, MASTL, CDC25C, CCSAP, KNSTRN, CIT, CCNA2, CCNA1, ASPM, PSRC1, **CCNE2**, KIFC1, CCNE1, INCENP, CKS2, **BIRC5**, FAM64A, MCM5, KIF2C, KIF20B, CDCA2, CDCA3, CDCA5, CDCA8, SKA3, PMF1, NSL1, AURKB, SKA1, NCAPH, AURKA, SKA2, CCNB2, CCNB1, DSN1, CDC45, RACGAP1, ECT2, FAM83D, BUB1, CENPW, SPAG5, UBE2C, PLK1, CDC7, CDC6, NDC80, ANLN, TPX2, CENPE, WEE1, APITD1, KIF18B, PRC1, UBE2S, CDK1, NCAPD2, CNTROB, SPC24, SPC25, MAD2L1
Mitotic Nuclear Division	6.98062E-40	6.624963972	ERCC6L, ZWILCH, NCAPG2, CCNF, BUB1B, KIF11, SMC2, CDC20, SGOL1, TUBB3, MIS18BP1, CHEK2, NUF2, NUSAP1, RCC1, NEK2, OIP5, **KNTC1**, FBXO5, SPDL1, CEP55, HELLS, BORA, KIF23, MASTL, CDC25C, CCSAP, KNSTRN, CIT, CCNA2, CCNA1, ASPM, PSRC1, ESPL1, INCENP, **BIRC5**, FAM64A, KIF2C, KIF20B, CDCA2, CDCA3, CDCA5, CDCA8, SKA3, PMF1, NSL1, AURKB, SKA1, NCAPH, AURKA, SKA2, CCNB2, CCNB1, DSN1, FAM83D, BUB1, CENPW, SPAG5, UBE2C, PLK1, CDC6, NDC80, ANLN, TPX2, CENPE, WEE1, APITD1, KIF18B, CENPH, CDK1, NCAPD2, CENPN, SPC24, SPC25, MAD2L1
DNA Replication	2.07287E-20	7.161424469	PIF1, FEN1, BLM, 2810417H13RIK, MCM7, MCM8, PRIM1, DSCC1, MCM10, BRCA1, CHTF18, CHAF1B, CDC45, CHAF1A, ORC1, POLE, GINS1, POLQ, GINS2, CDT1, RMI2, RRM1, RRM2, **RFC4**, LIG1, CDC6, ADRA2A, TICRR, POLA1, DBF4, POLE2, MCM3, MCM5, MCM6, DTL, MCM2
Chromosome Segregation	7.07731E-18	7.972784358	TOP2A, CDCA2, BRCA1, KIF11, SKA3, PMF1, NSL1, SKA1, SKA2, DSN1, SGOL1, RCC1, NEK2, OIP5, BUB1, CENPW, SPAG5, ESCO2, KNSTRN, NDC80, CENPE, CENPF, ESPL1, CENPH, INCENP, **BIRC5**, KIF2C, CENPN, SPC25
Cellular Response to DNA Damage Stimulus	1.16369E-10	2.854623365	TOP2A, FEN1, MCM7, MCM8, MCM10, BRCA1, FOXM1, BRCA2, PIDD1, CHAF1B, CHAF1A, EXO1, CHEK2, CHEK1, POLE, GEN1, LIG1, H2AFX, TRP73, MASTL, MMS22L, NEIL3, RAD51C, FANCD2, SPATA18, DTL, PIF1, 2810417H13RIK, BLM, PARPBP, **UHRF1**, UNG, RAD54B, RAD51AP1, BRIP1, RAD54L, CLSPN, E2F7, ZRANB3, BARD1, POLQ, EGLN3, ATAD5, DDIAS, TICRR, RAD51, APITD1, UBE2T, **CDKN3**
DNA Replication Initiation	7.46665E-10	12.23410014	POLA1, CDC45, **CCNE2**, MCM7, CCNE1, MCM3, CDC7, MCM5, MCM10, MCM6, CDC6, MCM2
DNA Repair	3.26326E-08	2.846928962	PIF1, FEN1, BLM, 2810417H13RIK, ANKLE1, PARPBP, MCM8, **UHRF1**, BRCA1, FOXM1, BRCA2, UNG, RAD51AP1, BRIP1, CHAF1B, CHAF1A, EXO1, CHEK2, CHEK1, RAD54L, CLSPN, POLE, ZRANB3, GEN1, BARD1, POLQ, LIG1, H2AFX, KIF22, TICRR, MMS22L, NEIL3, RAD51C, RAD51, APITD1, FANCD2, UBE2T
Chemokine-Mediated Signaling Pathway	2.33183E-07	6.22826916	CCL24, CCL12, **CCL11**, **CXCL1**, **CXCL3**, CXCL13, CXCL5, CCL9, CCL8, CCL7, CCR9, **CCL2**, ACKR1, PF4
Cellular Response to Tumor Necrosis Factor	4.7248E-07	4.226325501	CCL24, CCL12, **EDN1**, CALCA, **CCL11**, BRCA1, ADAMTS12, COL1A1, CCL9, CCL8, **FABP4**, CCL7, OCSTAMP, **CCL2**, HAS2, **PCK1**, **PPARGC1A**, DBN1, ADAMTS7
Cell Adhesion	6.56795E-07	2.270245386	PTPRU, SPON2, COL18A1, COL15A1, TENM3, COL16A1, TNFAIP6, LAMA1, TROAP, TNC, THBS2, NID2, WISP1, HAPLN1, CDH3, FLRT2, CDH2, CHL1, HAS1, **SPP1**, CLCA2, HAS2, CYP1B1, CDH24, NRCAM, NCAM1, EPHB1, AOC3, CADM4, MYBPC2, **FN1**, MFAP4, SDK1, VCAN, NFASC, COL5A1, COL6A2, CDHR1, COL6A1, **ADAM12**, COL6A4, CERCAM, CDHR3, COL6A6, COL6A5
Cellular Response to Interleukin-1	3.8958E-06	4.587787551	CCL24, CCL12, **EDN1**, **CCL11**, SAA3, FN1, ADAMTS12, CCL9, CCL8, CCL7, **CCL2**, HAS2, SOX9, **PCK1**, ADAMTS7
Collagen Fibril Organization	2.10752E-05	6.273897505	COL1A1, ADAMTS2, **COL3A1**, COL1A2, COL5A1, LUM, COL11A1, COL5A2, CYP1B1, LOXL2
Chemotaxis	2.41355E-05	3.5250797	CCL24, CCL12, **CCL11**, HMGB2, CXCR5, **CXCL3**, CXCL13, CXCL5, CCL9, CCL8, CCL7, CCR9, C3AR1, **CCL2**, **CCR5**, CCR3, PF4
Collagen Catabolic Process	3.4529E-05	8.156066757	ADAMTS2, MMP14, **MMP13**, CTSK, **MMP2**, MMP19, PRTN3, MMP10
G1/S Transition of Mitotic Cell Cycle	3.68705E-05	4.735780698	TCF19, CDKN2C, **CCNE2**, CCNE1, CDCA5, RCC1, RHOU, **INHBA**, SKP2, IQGAP3, POLE, **CDKN3**
G2/M Transition of Mitotic Cell Cycle	4.15239E-05	6.673145528	CHEK2, PLK1, CHEK1, **BIRC5**, MASTL, CDC25C, SKP2, FOXM1, CIT
Mitotic Spindle Assembly	6.54823E-05	6.291822927	CDC20, TPX2, KIFC1, CHEK2, KIFC5B, **BIRC5**, NEK2, MYBL2, KIF11
Protein Heterotrimerization	7.78534E-05	9.0146001	COL1A1, COL1A2, COL6A2, C1QTNF6, COL6A1, **ADIPOQ**, COL6A4
Aging	8.62644E-05	2.828693673	NTRK1, CALCA, **ARG1**, **SERPINF1**, ABAT, TYMS, ADRA1A, AURKB, **AGT**, **DCN**, ALDH3A1, **COL3A1**, KRT14, CYP1A1, **CCL2**, NCAM1, **TIMP1**, **APOE**, **PCK1**, **PPARGC1A**
Cytokinesis	9.99357E-05	5.95172439	PRC1, INCENP, PLK1, **BIRC5**, KIF23, KIF20A, ECT2, BRCA2, CIT
Cell Activation	0.000164448	10.48637154	LYPD1, **FN1**, LYPD2, **IGF1**, **TIMP1**, SLURP1
Inflammatory Response	0.000190111	2.133854675	CCL12, TPSB2, CALCA, **CCL11**, CD5L, HMGB2, **CXCL1**, REG3G, **CXCL3**, CXCL13, CXCL5, CCL9, CYP26B1, CCL8, CCL7, **SPP1**, C3AR1, **CCL2**, BDKRB1, **CCR5**, CCR3, CCL24, **CD163**, NR1H4, TRP73, CHIL3, HC, ACKR1, CHIL4, PF4
Positive Regulation of Protein Kinase C Activity	0.000262631	24.46820027	CEMIP, **WNT5A**, ROR2, **AGT**
Cellular Response to Retinoic Acid	0.000282918	4.140772353	COL1A1, CYP26A1, CYP26B1, BRINP1, TESC, **SERPINF1**, OSR1, TNC, **CCL2**, SOX9, **PCK1**
Response to Drug	0.000344462	2.093149876	COL18A1, NNMT, MCM7, HMGB2, ABAT, PTN, TYMS, ADRA1A, RAD54B, CCNB1, CDH3, LGALS1, MDK, RAD54L, NCAM1, **PPARGC1A**, ABCB1B, NTRK1, CBX7, **ARG1**, **MMP2**, TRP73, **INHBA**, COL1A1, ALDH3A1, RAD51, SCGB1A1, CYP1A1, CDK1
Cellular Response to Interferon-Gamma	0.000411696	3.95809122	CCL24, CCL9, CCL12, **EDN1**, CCL8, **CCL11**, GBP10, CCL7, **WNT5A**, **CCL2**, AQP4
Eosinophil Chemotaxis	0.000474593	12.23410014	CCL24, **CCL11**, CCL7, **CCL2**, CCR3
Endodermal Cell Differentiation	0.000642531	6.343607478	MMP14, COL11A1, **MMP2**, COL6A1, **FN1**, **INHBA**, NODAL
Male Gonad Development	0.000645266	3.058525034	RRM1, TESC, **WNT5A**, HMGB2, LRRC6, **INHBA**, ASPM, MMP14, BRIP1, WT1, SOX8, SOX9, WNT4, BOK
Spermatogenesis	0.000672503	1.923789702	LRGUK, HMGB2, BRCA2, CHTF18, AK7, C330027C09RIK, CLGN, BRIP1, ADAMTS2, CCNB1, CYP26B1, RACGAP1, **E2F1**, 1110017D15RIK, SOX8, SOX9, TSNAXIP1, CCDC33, TCP11, RPL39L, NDC1, H2AFX, NME5, CDC25C, CIT, CCNA1, ASPM, RAD51C, WT1, MYCBPAP, TRIP13, SPATA18

Cell Proliferation	0.000712836	2.335600935	MCM7, **UHRF1**, MCM10, **IGF1**, MKI67, BRCA2, AURKB, CKS1B, POLA1, UCHL1, MELK, CKS2, CYP1A1, CDK1, TACC3, FAM83D, BUB1, TSPAN1, **CFB**, E2F8, BOK
Extracellular Matrix Organization	0.000764012	3.0048667	VIT, COL18A1, OLFML2B, LAMA1, ELN, **FN1**, NID2, **AGT**, CCDC80, COL5A3, ADAMTSL2, COL6A4, VWA1, SOX9
Protein Phosphorylation	0.000768588	1.741660089	LAMA1, BUB1B, TTK, MST1R, PKMYT1, AURKB, AURKA, CCNB1, STK33, CHEK2, GSG2, CHEK1, PBK, NEK2, EPHB2, BUB1, EPHB1, PLK4, NTRK1, CDKL4, NEK5, **IGFBP3**, PLK1, **WNT5A**, CDC7, PASK, MASTL, MAPK15, DCLK1, CIT, MAPK10, WEE1, MELK, CCNE1, MAP3K19, CDK1, **BIRC5**, FAM20C, ROR2, CAMK1G, NEK11
Positive Regulation of Inflammatory Response	0.000982989	3.883841313	CCL24, CCL9, CCL12, CCL8, **CCL11**, **FABP4**, CCL7, **WNT5A**, **CCL2**, **CCR5**
Monocyte Chemotaxis	0.001053875	4.893640054	CCL24, CCL9, CCL12, CALCA, CCL8, **CCL11**, CCL7, **CCL2**
Collagen Biosynthetic Process	0.001234537	16.31213351	COL1A1, ADAMTS3, COL5A1, **ARG1**
Neutrophil Chemotaxis	0.001895059	3.546115981	CCL24, CCL9, CCL12, CCL8, **CCL11**, CCL7, **SPP1**, **CCL2**, **CXCL1**, **CXCL3**
Lymphocyte Chemotaxis	0.001952452	5.1902243	CCL24, CCL9, CCL12, CCL8, **CCL11**, CCL7, **CCL2**
Cellular Response to Platelet-Derived Growth Factor Stimulus	0.002036809	6.383008766	CCNA2, ERRFI1, HAS1, **CCL2**, HAS2, PTN
Positive Regulation of Transcription, DNA-Templated	0.002549274	1.65670106	MEG3, BLM, MYRF, HMGB2, BRCA1, FOXM1, BRCA2, CKS1B, MDK, CHEK2, **E2F1**, SOX8, SOX9, **PPARGC1A**, **FGF23**, WNT4, TESC, ATAD2, **WNT5A**, TRP73, EBF2, EBF3, **IGF1**, **INHBA**, GDF6, KLF15, ETV4, **AGT**, CCNA2, COL1A1, MLXIPL, PSRC1, WT1, LHX2, CKS2, NFE2L3, PGR, ROR2, ZFPM2
Cilium Movement	0.002670239	4.893640054	RSPH4A, **DNAH10**, DNAH5, DNAH6, LRRC6, DNALI1, CCDC40
Response to Lipopolysaccharide	0.003005409	2.235673121	SPON2, **EDN1**, WFDC21, SPARC, PTGER3, HMGB2, FMO1, **CXCL1**, **CXCL3**, CXCL13, LOXL1, CXCL5, **DCN**, SCGB1A1, CYP1A1, BDKRB1, **PCK1**, PF4
Cellular Response to Hypoxia	0.003196758	2.850664109	FABP1, CCNA2, CCNB1, **EDN1**, FNDC1, **E2F1**, TWIST1, **HMOX1**, PTN, **PCK1**, **PPARGC1A**, KCNK2
Maternal Process Involved In Parturition	0.0032506	12.23410014	**EDN1**, CYP1A1, **CCL2**, NODAL
Response to Activity	0.003537167	3.994808207	NTRK1, **EDN1**, **ADIPOQ**, CDK1, **CCL2**, PTN, **PCK1**, **PPARGC1A**
ERK1 and ERK2 Cascade	0.003598882	5.646507755	**CCL11**, **IGF1**, SOX9, IQGAP3, CCR3, **AGT**
Response to Mechanical Stimulus	0.00395037	3.495457182	**COL3A1**, MMP14, CCNB1, **MMP2**, TNC, **CCL2**, BDKRB1, KCNK2, **DCN**
Immune Response	0.004000488	1.97904561	H2-EB2, CCL24, CCL12, **CCL11**, CMA1, MCPT4, CXCR5, **CXCL1**, PRG4, **CXCL3**, CXCL13, CXCL5, VPREB3, CCL9, CCL8, CCL7, CCR9, **CCL2**, **ENPP3**, **CCR5**, FCGR2B, PF4
Positive Regulation of Cell Migration	0.004084037	2.169594113	COL18A1, LRRC15, CCL24, **EDN1**, CEMIP, **CCL11**, TNFAIP6, **MMP2**, ARHGEF39, **FN1**, **IGF1**, ADRA2A, COL1A1, MMP14, FAM110C, C3AR1, HAS2, ROR2
Cell Chemotaxis	0.004410917	3.136948753	CCL9, CCL12, CCL8, SAA3, HMGB2, **CCL2**, **CXCL1**, CXCL13, EPHB1, CXCL5
Spindle Checkpoint	0.004855424	24.46820027	**BIRC5**, SPDL1, AURKB
Wound Healing	0.004968517	2.863300032	**COL1A1**, COL3A1, CDH3, SPARC, SERPINB2, **WNT5A**, ALOX15, TNC, **FN1**, **TIMP1**, **DCN**
Negative Regulation of Neuron Apoptotic Process	0.005968007	2.293893775	GABRB3, NTRK1, CCL12, KIF14, TRP73, FAIM2, **AGT**, CHL1, MDK, **HMOX1**, **CCL2**, **BIRC5**, **APOE**, **PPARGC1A**, BOK
Regulation of Cell Cycle	0.006057104	2.621592886	FOXA1, 2810417H13RIK, **CCNE2**, CCNE1, FIGNL1, CCNF, **E2F1**, TACC3, MASTL, SKP2, DTL, PRR11
Complement Activation, Alternative Pathway	0.006551998	9.787280108	**CFD**, HC, CFP, **CFB**
Artery Smooth Muscle Contraction	0.006551998	9.787280108	SMPD3, **EDN1**, **AGT**, CACNA1G
Positive Regulation of Cell-Substrate Adhesion	0.006781905	4.078033378	VIT, CCDC80, ALOX15, **SPP1**, PTN, FBLN2, DBN1
Positive Regulation of Gtpase Activity	0.006846855	2.346265779	NTRK1, CCL24, CCL12, **CCL11**, RGS16, **WNT5A**, ELMOD1, CCL9, CCL8, CCL7, **CCL2**, ECT2, EZH2, WNT4
Negative Regulation of Neuron Death	0.00747482	3.495457182	GABRB3, NTRK1, CHGA, MDK, **SERPINF1**, **IGF1**, **APOE**, **PPARGC1A**
Positive Regulation of Cell-Cell Adhesion	0.007909363	6.117050068	**WNT5A**, **CCL2**, **CCR5**, NODAL, TBX18
Extracellular Fibril Organization	0.008738336	8.897527371	MFAP5, MFAP4, **COL3A1**, COL5A1
Negative Regulation of Angiogenesis	0.00887432	3.058525034	MEG3, SPARC, **SERPINF1**, **CCL2**, PTN, THBS2, **AGT**, **DCN**, PF4
Response to Axon Injury	0.009010065	4.587787551	NTRK1, LGALS1, **ARG1**, CDK1, FOLR1, KCNK2
Cellular Response to Fatty Acid	0.009460302	5.825761969	CCNB1, **EDN1**, **E2F1**, **UCP1**, NR1H4
Positive Regulation of Angiogenesis	0.010625841	2.426598374	CCL24, **CCL11**, CMA1, **WNT5A**, VASH2, C3AR1, CYP1B1, **HMOX1**, HC, BRCA1, CCR3, NODAL
Prostate Gland Epithelium Morphogenesis	0.01130184	8.156066757	FOXA1, **MMP2**, TNC, **IGF1**
Response to Gamma Radiation	0.011646232	4.317917695	CCL7, FANCD2, CHEK2, TRP73, **CCL2**, BRCA2
Cellular Response to Transforming Growth Factor Beta Stimulus	0.011813636	3.208944298	COL1A1, **EDN1**, **ARG1**, **WNT5A**, SOX9, **PPARGC1A**, DBN1, WNT4
Cell Migration	0.011825033	2.049692169	**MMP2**, **WNT5A**, GFRA1, ADAMTS12, MMP14, DEPDC1B, CDH2, COL5A1, MDK, FSCN1, BDKRB1, CD248, FAM83D, TSPAN1, NODAL, CTHRC1
Skeletal System Development	0.012080827	2.515422458	COL1A1, **COL3A1**, VCAN, **EDN1**, COL1A2, COL5A2, FAM20C, ROR2, SOX9, HAPLN1, FBN1
Cell Differentiation	0.012575311	1.442996426	MEG3, PTPRU, LRGUK, TENM3, MYRF, ILDR2, TWIST1, EFNA5, DMKN, CLGN, CDC20, RACGAP1, MDK, CHL1, OCSTAMP, INSC, STMN1, NHS, AGR3, 1110017D15RIK, MYBL2, SOX9, TSNAXIP1, CCHCR1, ECT2, MYBL1, **FGF23**, TCP11, WNT4, NTRK1, SRRM4, TESC, OSR1, **WNT5A**, NME5, DLK1, TBX18, DCLK1, CIT, CCNA1, MMP19, MYCBPAP, ROR2, TRIP13, SPATA18, DBN1
Embryo Development	0.015113426	2.787516487	MEG3, RACGAP1, **WNT5A**, CKS2, **BIRC5**, LRAT, BUB1, DLK1, CKS1B
Mast Cell Chemotaxis	0.015322628	14.68092016	CHGA, **CCL11**, CCR3
Positive Regulation of Fibroblast Proliferation	0.016408321	3.011470803	CCNA2, CCNB1, **WNT5A**, **E2F1**, **FN1**, **IGF1**, CDC6, **AGT**
Regulation of Mitotic Cell Cycle	0.016511596	3.96781626	PLK1, CKS2, TRP73, **BIRC5**, FBXO5, CKS1B
Negative Regulation of DNA Biosynthetic Process	0.017598693	6.990914363	MEG3, CHEK1, **ADIPOQ**, KCNK2
Cartilage Development	0.018582161	2.685534176	**EDN1**, **MMP13**, LUM, COL11A1, **WNT5A**, PRRX2, **TIMP1**, SOX9, TYMS
Positive Regulation of Endothelial Cell Proliferation	0.019125342	2.921576152	CCL24, **CCL11**, **ARG1**, **WNT5A**, VASH2, HMGB2, **CCL2**, CCR3
Cerebral Cortex Development	0.020595493	2.878611797	ASPM, **COL3A1**, CDH2, LHX2, MDK, H2AFX, TACC3, KIF14
Chromosome Organization Involved In Meiotic Cell Cycle	0.022366674	12.23410014	RAD51, **CCNE2**, CCNE1
Intramembranous Ossification	0.022366674	12.23410014	COL1A1, CTSK, **MMP2**
Regulation of Cell Proliferation	0.024179707	1.832420285	EGLN3, SPARC, TNC, CDCA7, PRG4, **IGF1**, BRCA1, **CXCL3**, CXCL13, FOXM1, BRCA2, CXCL5, NR3C2, CHEK1, SOX9, PF4, EZH2
Negative Regulation of Smooth Muscle Cell Migration	0.025488237	6.117050068	NDRG4, **IGFBP3**, **ADIPOQ**, **PPARGC1A**
Axon Regeneration	0.025488237	6.117050068	DHFR, CHL1, **APOE**, FOLR1

Positive Regulation of Cellular Protein Metabolic Process	0.025488237	6.117050068	**UHRF1**, **ADIPOQ**, **INHBA**, **AGT**
Negative Regulation of Inflammatory Response	0.025570731	2.531193131	MIR147, TNFAIP6, **SERPINF1**, **ADIPOQ**, PBK, NR1H4, **APOE**, CD276, IL22RA2
Organ Regeneration	0.026050043	3.058525034	CCNA2, PTPRU, NNMT, CDK1, **CCL2**, MKI67, CXCL5
Mitotic Cell Cycle Checkpoint	0.029058918	4.218655219	ZWILCH, CHEK1, **KNTC1**, TTK, MAD2L1
Lung Development	0.030531725	2.170566153	FOXA1, ADAMTS2, MMP14, SPARC, **ARG1**, **WNT5A**, **IGF1**, PTN, ZFPM2, NODAL, CCDC40
DNA Damage Checkpoint	0.032480729	4.078033378	CHEK2, H2AFX, CHEK1, **E2F1**, CLSPN
Synapsis	0.032480729	4.078033378	NDC1, BRIP1, **CCNE2**, FANCD2, CCNE1
Response to Glucocorticoid	0.033130294	2.609941362	ALDH3A1, PTPRU, SPARC, MDK, **PAPPA**, **ADIPOQ**, SCGB1A1, TYMS
Positive Regulation of Epithelial Cell Proliferation	0.035259736	2.575600028	OSR1, **WNT5A**, TWIST1, GAS1, **IGF1**, SOX9, NODAL, TBX18
Response to Nicotine	0.035533216	3.262426703	DHFR, NTRK1, **EDN1**, LYPD1, **HMOX1**, ABAT
Proteolysis	0.036506684	1.429413762	**CFD**, TPSB2, MYRF, MCPT4, CFI, ADAMTS12, MMP23, ADAMTS4, ADAMTS16, UCHL1, ADAM28, ADAMTS2, C1S1, CAPN6, ADAMTS19, CTSK, CLCA2, CPXM1, TPSAB1, ADAMTS7, CMA1, **MMP2**, ECEL1, PRSS35, MMP10, MMP14, **MMP13**, ESPL1, **PAPPA**, **ADAM12**, MMP19, PRTN3, **CFB**, PAPPA2
Positive Regulation of Cell Proliferation	0.037356911	1.44461699	SHC4, COL18A1, TNC, KIF14, PTN, FOXM1, CDC20, ESM1, HAS2, SOX9, **TIMP1**, FGFBP1, **EDN1**, **WNT5A**, CLEC11A, **FN1**, CDC7, **IGF1**, **AGT**, FABP1, ACER2, ALDH3A1, MLXIPL, CENPF, **FABP4**, PRC1, GAS1, PGR, CD248, KIF20B, FOLR2, NODAL
Positive Regulation of DNA-Directed DNA Polymerase Activity	0.039550273	9.175575101	**RFC4**, DSCC1, CHTF18
Astrocyte Cell Migration	0.039550273	9.175575101	CCL12, MMP14, **CCL2**
Heart Development	0.039676611	1.687462088	**EDN1**, SPARC, DNAH5, **MMP2**, OSR1, DAW1, FOXJ1, PTN, CENPF, VCAN, **COL3A1**, **MMP13**, WT1, SOX9, ZFPM2, DRC1, NODAL, FBN1
Positive Regulation of Mitotic Cell Cycle	0.040002162	3.823156292	FOXA1, CCNB1, CDK1, **BIRC5**, BRCA2
Regulation of Blood Pressure	0.040286262	2.76253874	AOC3, CALCA, **EDN1**, COL1A2, C3AR1, **HMOX1**, **AGT**
Osteoblast Differentiation	0.041634336	2.165327458	COL1A1, VCAN, FIGNL1, **IGFBP3**, COL6A1, TNC, **SPP1**, TWIST1, SOX8, **IGF1**
Ossification	0.042407478	2.293893775	MMP14, SPARC, COL11A1, COL5A2, ALOX15, **SPP1**, TWIST1, SOX9, PTN
Positive Regulation of Smooth Muscle Cell Proliferation	0.044674869	2.446820027	**EDN1**, ORC1, C3AR1, **HMOX1**, **IGF1**, SKP2, **PPARGC1A**, WISP1
Positive Regulation of ERK1 and ERK2 Cascade	0.044840304	1.82210002	NTRK1, CCL24, NDRG4, CCL12, **CCL11**, ALOX15, TREM2, ADRA1A, CCL9, CCL8, CCL7, **CCL2**, **FGF23**, NODAL
Brown Fat Cell Differentiation	0.048430684	3.598264746	MRAP, **FABP4**, **ADIPOQ**, **UCP1**, EBF2
Bone Trabecula Formation	0.049499272	8.156066757	FBN2, COL1A1, **MMP2**
Positive Regulation of Exit From Mitosis	0.049499272	8.156066757	UBE2C, CDCA5, **BIRC5**
Negative Regulation of Peptidase Activity	0.050054615	2.091299168	ITIH5, WFDC8, SERPINB2, CD109, **BIRC5**, BC048546, CST9, **TIMP1**, A2M, NAIP1
Placenta Development	0.052314903	2.936184032	GJB3, CCNF, E2F7, NODAL, **DCN**, E2F8
Negative Regulation of Blood Pressure	0.052984033	3.495457182	CALCA, PMCH, **ADIPOQ**, BDKRB1, ABAT
Regulation of Heart Rate	0.057761704	3.398361149	RYR2, CALCA, PMCH, **AGT**, CACNA1G
Positive Regulation of Leukocyte Migration	0.058577083	4.448763686	AOC3, CCL12, **CCL2**, BDKRB1
Cell-Cell Signaling	0.059674383	2.137998082	FGFBP1, **EDN1**, **WNT5A**, CXCL13, **CCR5**, ADRA1A, WISP1, ADRA2A, WNT4
Cellular Response to Vitamin D	0.06023609	7.340460081	TNC, PTN, **FGF23**
Positive Regulation of Mesenchymal Cell Proliferation	0.062761717	3.30651355	**WNT5A**, PRRX2, GAS1, SOX9, TBX18
Cochlea Morphogenesis	0.065394973	4.255339178	**WNT5A**, SOX9, TBX18, CTHRC1
Negative Regulation of Smooth Muscle Cell Proliferation	0.067981582	3.219500036	NDRG4, **IGFBP3**, **ADIPOQ**, **HMOX1**, **PPARGC1A**
Protein Kinase B Signaling	0.067981582	3.219500036	LINGO1, CCL12, **CCL2**, **IGF1**, SOX9
Intrinsic Apoptotic Signaling Pathway In Response to DNA Damage	0.068556158	2.718688919	CHEK2, **E2F1**, **HMOX1**, BRCA1, BRCA2, BOK
Cellular Response to Glucose Stimulus	0.069459154	2.412357773	MLXIPL, LGALS1, CMA1, **SERPINF1**, AQP4, **IGF1**, **PPARGC1A**
Regulation of Cellular Protein Localization	0.071680036	6.673145528	**CCNE2**, CCNE1, **WNT5A**
Negative Regulation of Immune Response	0.071680036	6.673145528	FCRLB, **COL3A1**, FCGR2B
Positive Regulation of Insulin-Like Growth Factor Receptor Signaling Pathway	0.071680036	6.673145528	CDH3, **IGFBP3**, **IGF1**
Negative Regulation of Tumor Necrosis Factor-Mediated Signaling Pathway	0.071680036	6.673145528	**ADIPOQ**, NR1H4, TRAIP
Middle Ear Morphogenesis	0.07255827	4.078033378	**EDN1**, OSR1, PRRX2, GAS1
Complement Activation, Classical Pathway	0.07300579	2.669258211	C1QB, C1QA, C1S1, CFI, HC, **C1QC**
Cell Fate Commitment	0.073295304	2.378852804	**WNT5A**, GAS1, SOX8, ROR2, SOX9, NODAL, WNT4
Regulation of Signal Transduction	0.079068535	3.058525034	CDH2, 2810408A11RIK, **BIRC5**, NAIP1, NODAL
Positive Regulation of Cytosolic Calcium Ion Concentration	0.081559448	1.818582453	**EDN1**, PTGER3, PMCH, C3AR1, **CXCL1**, CXCL13, **CCR5**, **CXCL3**, ADRA1A, **AGT**, CACNA1G
Cellular Response to Hydrogen Peroxide	0.082365321	2.575600028	FABP1, **ARG1**, CDK1, CYP1B1, ECT2, EZH2
Positive Regulation of Cell Growth Involved In Cardiac Muscle Cell Development	0.083755675	6.117050068	**EDN1**, **IGF1**, WISP1
Skin Development	0.087272288	2.531193131	COL1A1, ADAMTS2, **COL3A1**, COL5A1, GJB3, COL5A2
Nervous System Development	0.093627566	1.427852536	NTRK1, SRRM4, NRN1, D130043K22RIK, CRMP1, GFRA1, **IGF1**, EFNA5, DCLK1, CIT, CDC20, DCDC2A, LHX2, CHL1, DPF1, INSC, STMN1, ECT2, EPHB2, EPHB1, DBN1, NODAL
Response to Hypoxia	0.094858812	1.65670106	RYR2, **EDN1**, EGLN3, **MMP2**, **ADIPOQ**, ABAT, LOXL2, ALDH3A1, MMP14, CYP1A1, TACC3, **HMOX1**, **CCL2**
Odontogenesis	0.09599465	3.624918559	OSR1, TWIST1, GAS1, **INHBA**
Fatty Acid Homeostasis	0.096392539	5.646507755	MLXIPL, NR1H4, **APOE**
Short-Term Memory	0.096392539	5.646507755	BRINP1, MDK, **SERPINF1**
Negative Regulation of JAK-STAT Cascade	0.097259589	2.845139566	LRRC15, FLRT2, NYX, ASPN, **DCN**
Microtubule Cytoskeleton Organization	0.097407459	2.039016689	WEE1, GAS2L3, PRC1, TACC3, NUSAP1, CRMP1, **BIRC5**, AURKA
