# Supplementary material for: Inhibition of angiogenesis and regenerative lung growth in Lepob/ob mice through adiponectin-VEGF/VEGFR2 signaling
Source: Front Cardiovasc Med. 2024 Oct 16;11:1491971. doi: 10.3389/fcvm.2024.1491971 (PMC11521822; doi:10.3389/fcvm.2024.1491971)
Supplement: Supplementary file 4 [file Datasheet2.pdf]

Biological Processes	PValue	Fold Enrichment	Genes
Cell Cycle	2.58685E-52	4.98131113	ERCC6L, ZWILCH, DSCC1, GMNN, CCNF, DIXDC1, BUB1B, MKI67, SMC2, CDC20, CHEK2, CHEK1, NUSAP1, RCC1, NEK2, OIP5, KNTC1, FBXO5, BORA, LIG1, TRP73, ESCO2, CDC25C, KNSTRN, MELK, CCNE2, CCNE1, FANCD2, FAM64A, KIF20B, CDCA2, CDCA3, CDCA5, CDCA8, PKMYT1, SKA3, PMF1, NCAPH, SKA1, SKA2, CCNB2, DSN1, CCNB1, BRINP1, RACGAP1, CLSPN, ECT2, FAM83D, CDT1, UBE2C, DDIAS, PLK1, CDC7, CDC6, NDC80, ANLN, TPX2, KIF18B, UBE2S, CDK1, CNTROB, MCM7, MCM8, NCAPG2, BRCA1, KIF11, CHTF18, FOXM1, BRCA2, CKS1B, SMPD3, CHAF1B, CHAF1A, SGOL1, MIS18BP1, GSG2, NUF2, MYBL2, SPDL1, DLGAP5, CEP55, HELLS, H2AFX, CKAP2, KIF23, MASTL, CCSAP, CIT, CCNA2, ASPM, CCNA1, DBF4, PSRC1, INCENP, MCM3, CKS2, BIRC5, GAS1, MCM5, KIF2C, MCM6, MCM2, UHRF1, NSL1, AURKB, AURKA, CDC45, E2F1, BUB1, E2F7, E2F8, CENPW, CDKN2C, SPAG5, TICRR, CENPE, WEE1, APITD1, PRC1, NCAPD2, SPC24, MAD2L1, SPC25, NEK11, CDKN3
Cell Division	9.92865E-41	5.691800598	ERCC6L, ZWILCH, NCAPG2, CCNF, KIF14, BUB1B, KIF11, SMC2, CKS1B, CDC20, SGOL1, MIS18BP1, CHEK2, NUF2, NUSAP1, RCC1, NEK2, OIP5, KNTC1, FBXO5, SPDL1, CEP55, HELLS, BORA, LIG1, KIFC5B, KIF23, MASTL, CDC25C, CCSAP, KNSTRN, CIT, CCNA2, CCNA1, ASPM, PSRC1, CCNE2, KIFC1, CCNE1, INCENP, CKS2, BIRC5, FAM64A, MCM5, KIF2C, KIF20B, CDCA2, CDCA3, CDCA5, CDCA8, SKA3, PMF1, NSL1, AURKB, SKA1, NCAPH, AURKA, SKA2, CCNB2, CCNB1, DSN1, CDC45, RACGAP1, ECT2, FAM83D, BUB1, CENPW, SPAG5, UBE2C, PLK1, CDC7, CDC6, NDC80, ANLN, TPX2, CENPE, WEE1, APITD1, KIF18B, PRC1, UBE2S, CDK1, NCAPD2, CNTROB, SPC24, SPC25, MAD2L1
Mitotic Nuclear Division	6.98062E-40	6.624963972	ERCC6L, ZWILCH, NCAPG2, CCNF, BUB1B, KIF11, SMC2, CDC20, SGOL1, TUBB3, MIS18BP1, CHEK2, NUF2, NUSAP1, RCC1, NEK2, OIP5, KNTC1, FBXO5, SPDL1, CEP55, HELLS, BORA, KIF23, MASTL, CDC25C, CCSAP, KNSTRN, CIT, CCNA2, CCNA1, ASPM, PSRC1, ESPL1, INCENP, BIRC5, FAM64A, KIF2C, KIF20B, CDCA2, CDCA3, CDCA5, CDCA8, SKA3, PMF1, NSL1, AURKB, SKA1, NCAPH, AURKA, SKA2, CCNB2, CCNB1, DSN1, FAM83D, BUB1, CENPW, SPAG5, UBE2C, PLK1, CDC6, NDC80, ANLN, TPX2, CENPE, WEE1, APITD1, KIF18B, CENPH, CDK1, NCAPD2, CENPN, SPC24, SPC25, MAD2L1
DNA Replication	2.07287E-20	7.161424469	PIF1, FEN1, BLM, 2810417H13RIK, MCM7, MCM8, PRIM1, DSCC1, MCM10, BRCA1, CHTF18, CHAF1B, CDC45, CHAF1A, ORC1, POLE, GINS1, POLQ, GINS2, CDT1, RMI2, RRM1, RRM2, RFC4, LIG1, CDC6, ADRA2A, TICRR, POLA1, DBF4, POLE2, MCM3, MCM5, MCM6, DTL, MCM2
Chromosome Segregation	7.07731E-18	7.972784358	TOP2A, CDCA2, BRCA1, KIF11, SKA3, PMF1, NSL1, SKA1, SKA2, DSN1, SGOL1, RCC1, NEK2, OIP5, BUB1, CENPW, SPAG5, ESCO2, KNSTRN, NDC80, CENPE, CENPF, ESPL1, CENPH, INCENP, BIRC5, KIF2C, CENPN, SPC25
Mitotic Sister Chromatid Segregation	6.16451E-13	14.89368712	SPAG5, PLK1, CDCA8, CENPA, KNSTRN, NSL1, NDC80, CIT, DSN1, KIF18B, ESPL1, NUSAP1, NEK2, MAD2L1
Cellular Response to DNA Damage Stimulus	1.16369E-10	2.854623365	TOP2A, FEN1, MCM7, MCM8, MCM10, BRCA1, FOXM1, BRCA2, PIDD1, CHAF1B, CHAF1A, EXO1, CHEK2, CHEK1, POLE, GEN1, LIG1, H2AFX, TRP73, MASTL, MMS22L, NEIL3, RAD51C, FANCD2, SPATA18, DTL, PIF1, 2810417H13RIK, BLM, PARPBP, UHRF1, UNG, RAD54B, RAD51AP1, BRIP1, RAD54L, CLSPN, E2F7, ZRANB3, BARD1, POLQ, EGLN3, ATAD5, DDIAS, TICRR, RAD51, APITD1, UBE2T, CDKN3
DNA Replication Initiation	7.46665E-10	12.23410014	POLA1, CDC45, CCNE2, MCM7, CCNE1, MCM3, CDC7, MCM5, MCM10, MCM6, CDC6, MCM2
Microtubule-Based Movement	1.22575E-08	5.646507755	DNAH5, KIFC5B, KIF14, KIF23, DNAH9, KIF11, KIF22, KIF27, KIF15, KIF4, CENPE, KIF18A, KIF18B, KIFC1, DYNLRB2, KIF2C, KIF20A, KIF20B
DNA Repair	3.26326E-08	2.846928962	PIF1, FEN1, BLM, 2810417H13RIK, ANKLE1, PARPBP, MCM8, UHRF1, BRCA1, FOXM1, BRCA2, UNG, RAD51AP1, BRIP1, CHAF1B, CHAF1A, EXO1, CHEK2, CHEK1, RAD54L, CLSPN, POLE, ZRANB3, GEN1, BARD1, POLQ, LIG1, H2AFX, KIF22, TICRR, MMS22L, NEIL3, RAD51C, RAD51, APITD1, FANCD2, UBE2T
Mitotic Metaphase Plate Congression	5.88643E-08	8.63583539	CENPE, KIF18A, CCNB1, PSRC1, KIFC1, CDCA5, KIFC5B, KIF14, CDCA8, KIF2C, SPDL1, KIF22
Mitotic Cytokinesis	1.7265E-07	8.971673433	ANLN, RACGAP1, PLK1, STMN1, NUSAP1, CKAP2, KIF23, KIF20A, CENPA, CEP55, KIF4
Chemokine-Mediated Signaling Pathway	2.33183E-07	6.22826916	CCL24, CCL12, CCL11, CXCL1, CXCL3, CXCL13, CXCL5, CCL9, CCL8, CCL7, CCR9, CCL2, ACKR1, PF4
Cellular Response to Tumor Necrosis Factor	4.7248E-07	4.226325501	CCL24, CCL12, EDN1, CALCA, CCL11, BRCA1, ADAMTS12, COL1A1, CCL9, CCL8, FABP4, CCL7, OCSTAMP, CCL2, HAS2, PCK1, PPARGC1A, DBN1, ADAMTS7
Cell Adhesion	6.56795E-07	2.270245386	PTPRU, SPON2, COL18A1, COL15A1, TENM3, COL16A1, TNFAIP6, LAMA1, TROAP, TNC, THBS2, NID2, WISP1, HAPLN1, CDH3, FLRT2, CDH2, CHL1, HAS1, SPP1, CLCA2, HAS2, CYP1B1, CDH24, NRCAM, NCAM1, EPHB1, AOC3, CADM4, MYBPC2, FN1, MFAP4, SDK1, VCAN, NFASC, COL5A1, COL6A2, CDHR1, COL6A1, ADAM12, COL6A4, CERCAM, CDHR3, COL6A6, COL6A5
Metaphase Plate Congression	3.38964E-06	14.27311682	CENPE, CENPF, KIF2C, KIF22, FAM83D, CENPQ, NDC80
Cellular Response to Interleukin-1	3.8958E-06	4.587787551	CCL24, CCL12, EDN1, CCL11, SAA3, FN1, ADAMTS12, CCL9, CCL8, CCL7, CCL2, HAS2, SOX9, PCK1, ADAMTS7
Double-Strand Break Repair via Homologous Recombination	1.74004E-05	4.677744169	GEN1, BLM, MCM8, H2AFX, TONSL, BRCA1, BRCA2, RAD54B, RAD51AP1, MMS22L, RAD51, RAD51C, RAD54L
Collagen Fibril Organization	2.10752E-05	6.273897505	COL1A1, ADAMTS2, COL3A1, COL1A2, COL5A1, LUM, COL11A1, COL5A2, CYP1B1, LOXL2
Chemotaxis	2.41355E-05	3.5250797	CCL24, CCL12, CCL11, HMGB2, CXCR5, CXCL3, CXCL13, CXCL5, CCL9, CCL8, CCL7, CCR9, C3AR1, CCL2, CCR5, CCR3, PF4
Collagen Catabolic Process	3.4529E-05	8.156066757	ADAMTS2, MMP14, MMP13, CTSK, MMP2, MMP19, PRTN3, MMP10
G1/S Transition of Mitotic Cell Cycle	3.68705E-05	4.735780698	TCF19, CDKN2C, CCNE2, CCNE1, CDCA5, RCC1, RHOU, INHBA, SKP2, IQGAP3, POLE, CDKN3
G2/M Transition of Mitotic Cell Cycle	4.15239E-05	6.673145528	CHEK2, PLK1, CHEK1, BIRC5, MASTL, CDC25C, SKP2, FOXM1, CIT
Positive Regulation of Cytokinesis	4.15239E-05	6.673145528	RACGAP1, CXCR5, KIF14, KIF23, CDC6, KIF20B, ECT2, AURKB, CIT
Mitotic Spindle Assembly	6.54823E-05	6.291822927	CDC20, TPX2, KIFC1, CHEK2, KIFC5B, BIRC5, NEK2, MYBL2, KIF11
Protein Heterotrimerization	7.78534E-05	9.0146001	COL1A1, COL1A2, COL6A2, C1QTNF6, COL6A1, ADIPOQ, COL6A4

Aging	8.62644E-05	2.828693673	NTRK1, CALCA, ARG1, SERPINF1, ABAT, TYMS, ADRA1A, AURKB, AGT, DCN, ALDH3A1, COL3A1, KRT14, CYP1A1, CCL2, NCAM1, TIMP1, APOE, PCK1, PPARGC1A
Regulation of Attachment of Spindle Microtubules to Kinetochore	8.72876E-05	17.47728591	RACGAP1, SPAG5, NEK2, ECT2, KNSTRN
Cytokinesis	9.99357E-05	5.95172439	PRC1, INCENP, PLK1, BIRC5, KIF23, KIF20A, ECT2, BRCA2, CIT
Mitotic Spindle Organization	0.00010243	6.990914363	STIL, CCNB1, STMN1, RCC1, AURKB, NDC80, SPC25, AURKA
Mitotic Chromosome Condensation	0.000109384	11.29301551	CDCA5, NUSAP1, NCAPG, NCAPD2, NCAPH, SMC2
Cell Activation	0.000164448	10.48637154	LYPD1, FN1, LYPD2, IGF1, TIMP1, SLURP1
Inflammatory Response	0.000190111	2.133854675	CCL12, TPSB2, CALCA, CCL11, CD5L, HMGB2, CXCL1, REG3G, CXCL3, CXCL13, CXCL5, CCL9, CYP26B1, CCL8, CCL7, SPP1, C3AR1, CCL2, BDKRB1, CCR5, CCR3, CCL24, CD163, NR1H4, TRP73, CHIL3, HC, ACKR1, CHIL4, PF4
Positive Regulation of Protein Kinase C Activity	0.000262631	24.46820027	CEMIP, WNT5A, ROR2, AGT
Cellular Response to Retinoic Acid	0.000282918	4.140772353	COL1A1, CYP26A1, CYP26B1, BRINP1, TESC, SERPINF1, OSR1, TNC, CCL2, SOX9, PCK1
Activation of Protein Kinase Activity	0.000309398	5.931684914	TPX2, CALCA, CKS2, KIF14, CLSPN, ECT2, ADRA2A, CKS1B
Chromosome Condensation	0.000335176	9.175575101	TOP2A, NCAPG2, CDK1, NCAPD2, NCAPH, SMC2
Spindle Organization	0.000335176	9.175575101	ASPM, SPAG5, KIF11, KNSTRN, AURKB, AURKA
Response to Drug	0.000344462	2.093149876	COL18A1, NNMT, MCM7, HMGB2, ABAT, PTN, TYMS, ADRA1A, RAD54B, CCNB1, CDH3, LGALS1, MDK, RAD54L, NCAM1, PPARGC1A, ABCB1B, NTRK1, CBX7, ARG1, MMP2, TRP73, INHBA, COL1A1, ALDH3A1, RAD51, SCGB1A1, CYP1A1, CDK1
Axon Guidance	0.000393367	2.791673856	NTRK1, MEG3, LAMA1, WNT5A, CRMP1, EFNA5, EFNA4, NFASC, FLRT2, LHX2, TUBB3, CHL1, GAS1, NCAM1, NRCAM, EPHB2, EPHB1
Cellular Response to Interferon-Gamma	0.000411696	3.95809122	CCL24, CCL9, CCL12, EDN1, CCL8, CCL11, GBP10, CCL7, WNT5A, CCL2, AQP4
Eosinophil Chemotaxis	0.000474593	12.23410014	CCL24, CCL11, CCL7, CCL2, CCR3
DNA Unwinding Involved In DNA Replication	0.000474593	12.23410014	TOP2A, RAD51, MCM7, MCM6, MCM2
Protein Localization to Kinetochore	0.000474593	12.23410014	CDK1, BUB1B, TTK, SPDL1, AURKB
Mitotic Cell Cycle Phase Transition	0.000636599	19.57456022	CKS2, CDK1, NEK11, CKS1B
Endodermal Cell Differentiation	0.000642531	6.343607478	MMP14, COL11A1, MMP2, COL6A1, FN1, INHBA, NODAL
Microtubule Bundle Formation	0.000642531	6.343607478	TTLL6, PSRC1, CAPN6, PRC1, PLK1, KIF20A, GAS2L2
Male Gonad Development	0.000645266	3.058525034	RRM1, TESC, WNT5A, HMGB2, LRRC6, INHBA, ASPM, MMP14, BRIP1, WT1, SOX8, SOX9, WNT4, BOK
DNA Recombination	0.000661701	3.454334156	RAD51AP1, PIF1, BLM, RAD51, RAD51C, LIG1, EXO1, H2AFX, HMGB2, BRCA1, BRCA2, RAG1
Spermatogenesis	0.000672503	1.923789702	LRGUK, HMGB2, BRCA2, CHTF18, AK7, C330027C09RIK, CLGN, BRIP1, ADAMTS2, CCNB1, CYP26B1, RACGAP1, E2F1, 1110017D15RIK, SOX8, SOX9, TSNAXIP1, CCDC33, TCP11, RPL39L, NDC1, H2AFX, NME5, CDC25C, CIT, CCNA1, ASPM, RAD51C, WT1, MYCBPAP, TRIP13, SPATA18
Cell Proliferation	0.000712836	2.335600935	MCM7, UHRF1, MCM10, IGF1, MKI67, BRCA2, AURKB, CKS1B, POLA1, UCHL1, MELK, CKS2, CYP1A1, CDK1, TACC3, FAM83D, BUB1, TSPAN1, CFB, E2F8, BOK
Extracellular Matrix Organization	0.000764012	3.0048667	VIT, COL18A1, OLFML2B, LAMA1, ELN, FN1, NID2, AGT, CCDC80, COL5A3, ADAMTSL2, COL6A4, VWA1, SOX9
Protein Phosphorylation	0.000768588	1.741660089	LAMA1, BUB1B, TTK, MST1R, PKMYT1, AURKB, AURKA, CCNB1, STK33, CHEK2, GSG2, CHEK1, PBK, NEK2, EPHB2, BUB1, EPHB1, PLK4, NTRK1, CDKL4, NEK5, IGFBP3, PLK1, WNT5A, CDC7, PASK, MASTL, MAPK15, DCLK1, CIT, MAPK10, WEE1, MELK, CCNE1, MAP3K19, CDK1, BIRC5, FAM20C, ROR2, CAMK1G, NEK11
Positive Regulation of Inflammatory Response	0.000982989	3.883841313	CCL24, CCL9, CCL12, CCL8, CCL11, FABP4, CCL7, WNT5A, CCL2, CCR5
Regulation of Mitotic Nuclear Division	0.001038661	7.340460081	CENPE, BORA, RCC1, FBXO5, MKI67, KIF20B
Monocyte Chemotaxis	0.001053875	4.893640054	CCL24, CCL9, CCL12, CALCA, CCL8, CCL11, CCL7, CCL2
Collagen Biosynthetic Process	0.001234537	16.31213351	COL1A1, ADAMTS3, COL5A1, ARG1
Mitotic Spindle Midzone Assembly	0.001234537	16.31213351	RACGAP1, KIF23, AURKB, KIF4
G2 DNA Damage Checkpoint	0.001317871	6.990914363	PLK1, CHEK1, CLSPN, BRCA1, DTL, NEK11
Axoneme Assembly	0.00164878	6.673145528	SPAG16, LRGUK, RSPH1, RSPH4A, AK7, CCDC40
Neutrophil Chemotaxis	0.001895059	3.546115981	CCL24, CCL9, CCL12, CCL8, CCL11, CCL7, SPP1, CCL2, CXCL1, CXCL3
Lymphocyte Chemotaxis	0.001952452	5.1902243	CCL24, CCL9, CCL12, CCL8, CCL11, CCL7, CCL2
Microtubule Depolymerization	0.001985089	8.738642954	KIF18A, KIF18B, STMN1, KIF14, KIF2C
Cellular Response to Platelet-Derived Growth Factor Stimulus	0.002036809	6.383008766	CCNA2, ERRFI1, HAS1, CCL2, HAS2, PTN
Phosphorylation	0.00236113	1.639209495	LRGUK, BUB1B, TTK, MST1R, AK5, PKMYT1, AK7, AURKB, AURKA, STK33, CHEK2, GSG2, CHEK1, PBK, NEK2, TK1, EPHB2, BUB1, EPHB1, PLK4, NTRK1, CDKL4, NEK5, PLK1, CDC7, NME4, PASK, MASTL, MAPK15, DCLK1, CIT, MAPK10, WEE1, MELK, MAP3K19, ITPKA, CDK1, FAM20C, ROR2, CAMK1G, NEK11
Replication Fork Processing	0.002487463	6.117050068	ZRANB3, MMS22L, BLM, RAD51, APITD1, TONSL
Positive Regulation of Transcription, DNA-Templated	0.002549274	1.65670106	MEG3, BLM, MYRF, HMGB2, BRCA1, FOXM1, BRCA2, CKS1B, MDK, CHEK2, E2F1, SOX8, SOX9, PPARGC1A, FGF23, WNT4, TESC, ATAD2, WNT5A, TRP73, EBF2, EBF3, IGF1, INHBA, GDF6, KLF15, ETV4, AGT, CCNA2, COL1A1, MLXIPL, PSRC1, WT1, LHX2, CKS2, NFE2L3, PGR, ROR2, ZFPM2
Cilium Movement	0.002670239	4.893640054	RSPH4A, DNAH10, DNAH5, DNAH6, LRRC6, DNALI1, CCDC40

Response to Lipopolysaccharide	0.003005409	2.235673121	SPON2, EDN1, WFDC21, SPARC, PTGER3, HMGB2, FMO1, CXCL1, CXCL3, CXCL13, LOXL1, CXCL5, DCN, SCGB1A1, CYP1A1, BDKRB1, PCK1, PF4
Cellular Response to Hypoxia	0.003196758	2.850664109	FABP1, CCNA2, CCNB1, EDN1, FNDC1, E2F1, TWIST1, HMOX1, PTN, PCK1, PPARGC1A, KCNK2
Regulation of Mitotic Spindle Organization	0.0032506	12.23410014	TPX2, BORA, PSRC1, TACC3
Maternal Process Involved In Parturition	0.0032506	12.23410014	EDN1, CYP1A1, CCL2, NODAL
Response to Activity	0.003537167	3.994808207	NTRK1, EDN1, ADIPOQ, CDK1, CCL2, PTN, PCK1, PPARGC1A
ERK1 and ERK2 Cascade	0.003598882	5.646507755	CCL11, IGF1, SOX9, IQGAP3, CCR3, AGT
Response to Mechanical Stimulus	0.00395037	3.495457182	COL3A1, MMP14, CCNB1, MMP2, TNC, CCL2, BDKRB1, KCNK2, DCN
Double-Strand Break Repair	0.00395037	3.495457182	POLQ, LIG1, CHEK2, CDCA5, RAD54L, TRIP13, ESCO2, BRCA1, BRCA2
Immune Response	0.004000488	1.97904561	H2-EB2, CCL24, CCL12, CCL11, CMA1, MCPT4, CXCR5, CXCL1, PRG4, CXCL3, CXCL13, CXCL5, VPREB3, CCL9, CCL8, CCL7, CCR9, CCL2, ENPP3, CCR5, FCGR2B, PF4
Positive Regulation of Cell Migration	0.004084037	2.169594113	COL18A1, LRRC15, CCL24, EDN1, CEMIP, CCL11, TNFAIP6, MMP2, ARHGEF39, FN1, IGF1, ADRA2A, COL1A1, MMP14, FAM110C, C3AR1, HAS2, ROR2
Cell Chemotaxis	0.004410917	3.136948753	CCL9, CCL12, CCL8, SAA3, HMGB2, CCL2, CXCL1, CXCL13, EPHB1, CXCL5
Protein K6-Linked Ubiquitination	0.004728777	10.87475568	BARD1, UBE2S, UBE2T, BRCA1
Kinetochore Assembly	0.004728777	10.87475568	CENPW, APITD1, CENPH, CENPA
Attachment of Spindle Microtubules to Kinetochore	0.004728777	10.87475568	CENPE, SGOL1, NUF2, NDC80
Regulation of Mitotic Metaphase/Anaphase Transition	0.004728777	10.87475568	CENPE, UBE2C, PLK1, CDC6
Spindle Checkpoint	0.004855424	24.46820027	BIRC5, SPDL1, AURKB
Retinal Rod Cell Differentiation	0.004855424	24.46820027	SOX8, SOX9, PTN
Renal Vesicle Induction	0.004855424	24.46820027	SOX8, SOX9, WNT4
Wound Healing	0.004968517	2.863300032	COL1A1, COL3A1, CDH3, SPARC, SERPINB2, WNT5A, ALOX15, TNC, FN1, TIMP1, DCN
Mitotic Spindle Assembly Checkpoint	0.005329038	6.796722297	CENPE, PLK1, BUB1B, BUB1, MAD2L1
Negative Regulation of Neuron Apoptotic Process	0.005968007	2.293893775	GABRB3, NTRK1, CCL12, KIF14, TRP73, FAIM2, AGT, CHL1, MDK, HMOX1, CCL2, BIRC5, APOE, PPARGC1A, BOK
Regulation of Cell Cycle	0.006057104	2.621592886	FOXA1, 2810417H13RIK, CCNE2, CCNE1, FIGNL1, CCNF, E2F1, TACC3, MASTL, SKP2, DTL, PRR11
Regulation of Microtubule Polymerization Or Depolymerization	0.006551998	9.787280108	STMN1, SKA3, SKA1, SKA2
Positive Regulation of Keratinocyte Proliferation	0.006551998	9.787280108	TGM1, CDH3, HAS2, REG3G
Anaphase-Promoting Complex-Dependent Catabolic Process	0.006551998	9.787280108	CDC20, 1500015O10RIK, UBE2S, UBE2C
Complement Activation, Alternative Pathway	0.006551998	9.787280108	CFD, HC, CFP, CFB
Artery Smooth Muscle Contraction	0.006551998	9.787280108	SMPD3, EDN1, AGT, CACNA1G
Positive Regulation of Cell-Substrate Adhesion	0.006781905	4.078033378	VIT, CCDC80, ALOX15, SPP1, PTN, FBLN2, DBN1
Positive Regulation of Gtpase Activity	0.006846855	2.346265779	NTRK1, CCL24, CCL12, CCL11, RGS16, WNT5A, ELMOD1, CCL9, CCL8, CCL7, CCL2, ECT2, EZH2, WNT4
Negative Regulation of Neuron Death	0.00747482	3.495457182	GABRB3, NTRK1, CHGA, MDK, SERPINF1, IGF1, APOE, PPARGC1A
Positive Regulation of Synapse Assembly	0.007512384	3.145911463	NTRK1, FLRT2, SLITRK4, TPBG, LRRN1, THBS2, EPHB2, EFNA5, EPHB1
Positive Regulation of Cell-Cell Adhesion	0.007909363	6.117050068	WNT5A, CCL2, CCR5, NODAL, TBX18
Response to Ionizing Radiation	0.008229123	3.434133371	RRM1, RAD51, RAD51C, KRT14, RAD54L, BRCA1, TICRR, RAD54B
Extracellular Fibril Organization	0.008738336	8.897527371	MFAP5, MFAP4, COL3A1, COL5A1
Negative Regulation of Angiogenesis	0.00887432	3.058525034	MEG3, SPARC, SERPINF1, CCL2, PTN, THBS2, AGT, DCN, PF4
Response to Axon Injury	0.009010065	4.587787551	NTRK1, LGALS1, ARG1, CDK1, FOLR1, KCNK2
Regulation of Mitotic Centrosome Separation	0.009448217	18.3511502	CHEK1, NEK2, KIF11
Cellular Response to Fatty Acid	0.009460302	5.825761969	CCNB1, EDN1, E2F1, UCP1, NR1H4
Positive Regulation of Angiogenesis	0.010625841	2.426598374	CCL24, CCL11, CMA1, WNT5A, VASH2, C3AR1, CYP1B1, HMOX1, HC, BRCA1, CCR3, NODAL
Calcium Ion Transport Into Cytosol	0.01130184	8.156066757	CACNA1I, RYR2, ADRA1A, CACNA1G
Prostate Gland Epithelium Morphogenesis	0.01130184	8.156066757	FOXA1, MMP2, TNC, IGF1
DNA Damage Response, Signal Transduction By P53 Class Mediator Resulting In Transcription of P21 Class Mediator	0.01130184	8.156066757	CHEK2, TRP73, FOXM1, BRCA2
Response to Gamma Radiation	0.011646232	4.317917695	CCL7, FANCD2, CHEK2, TRP73, CCL2, BRCA2
Cellular Response to Transforming Growth Factor Beta Stimulus	0.011813636	3.208944298	COL1A1, EDN1, ARG1, WNT5A, SOX9, PPARGC1A, DBN1, WNT4
Cell Migration	0.011825033	2.049692169	MMP2, WNT5A, GFRA1, ADAMTS12, MMP14, DEPDC1B, CDH2, COL5A1, MDK, FSCN1, BDKRB1, CD248, FAM83D, TSPAN1, NODAL, CTHRC1
Skeletal System Development	0.012080827	2.515422458	COL1A1, COL3A1, VCAN, EDN1, COL1A2, COL5A2, FAM20C, ROR2, SOX9, HAPLN1, FBN1
Cell Differentiation	0.012575311	1.442996426	MEG3, PTPRU, LRGUK, TENM3, MYRF, ILDR2, TWIST1, EFNA5, DMKN, CLGN, CDC20, RACGAP1, MDK, CHL1, OCSTAMP, INSC, STMN1, NHS, AGR3, 1110017D15RIK, MYBL2, SOX9, TSNAXIP1, CCHCR1, ECT2, MYBL1, FGF23, TCP11, WNT4, NTRK1, SRRM4, TESC, OSR1, WNT5A, NME5, DLK1, TBX18, DCLK1, CIT, CCNA1, MMP19, MYCBPAP, ROR2, TRIP13, SPATA18, DBN1
Embryo Development	0.015113426	2.787516487	MEG3, RACGAP1, WNT5A, CKS2, BIRC5, LRAT, BUB1, DLK1, CKS1B

Positive Regulation of Vascular Endothelial Growth Factor Production	0.015251893	5.097541723	C3AR1, CYP1B1, HC, BRCA1, NODAL
Protein Localization to Chromosome, Centromeric Region	0.015322628	14.68092016	GSG2, BUB1B, CENPA
Double-Strand Break Repair via Break-Induced Replication	0.015322628	14.68092016	GINS2, CDC45, CDC7
Metaphase/Anaphase Transition of Mitotic Cell Cycle	0.015322628	14.68092016	TACC3, BUB1B, CIT
Mast Cell Chemotaxis	0.015322628	14.68092016	CHGA, CCL11, CCR3
Eye Morphogenesis	0.015322628	14.68092016	COL5A1, COL5A2, GAS1
Positive Regulation of Fibroblast Proliferation	0.016408321	3.011470803	CCNA2, CCNB1, WNT5A, E2F1, FN1, IGF1, CDC6, AGT
Regulation of Mitotic Cell Cycle	0.016511596	3.96781626	PLK1, CKS2, TRP73, BIRC5, FBXO5, CKS1B
Negative Regulation of DNA Biosynthetic Process	0.017598693	6.990914363	MEG3, CHEK1, ADIPOQ, KCNK2
Mitotic Cell Cycle	0.01839287	3.863400043	CENPE, CENPF, RRM1, CENPW, KIF18B, AURKA
Chromosome Organization	0.01839287	3.863400043	BLM, CENPW, CDCA8, RAD54L, BRCA2, SMC2
Cartilage Development	0.018582161	2.685534176	EDN1, MMP13, LUM, COL11A1, WNT5A, PRRX2, TIMP1, SOX9, TYMS
Positive Regulation of Endothelial Cell Proliferation	0.019125342	2.921576152	CCL24, CCL11, ARG1, WNT5A, VASH2, HMGB2, CCL2, CCR3
Cerebral Cortex Development	0.020595493	2.878611797	ASPM, COL3A1, CDH2, LHX2, MDK, H2AFX, TACC3, KIF14
Retinoic Acid Metabolic Process	0.021343305	6.524853406	CYP26A1, ALDH1A3, CYP26B1, LRAT
Nucleoside Diphosphate Phosphorylation	0.021343305	6.524853406	NME4, NME5, AK5, AK7
Regulation of Double-Strand Break Repair via Homologous Recombination	0.021343305	6.524853406	RAD51AP1, RAD51, FIGNL1, CHEK1
Chromosome Organization Involved In Meiotic Cell Cycle	0.022366674	12.23410014	RAD51, CCNE2, CCNE1
Negative Regulation of Centrosome Duplication	0.022366674	12.23410014	KIFC1, CCNF, KIFC5B
Intramembranous Ossification	0.022366674	12.23410014	COL1A1, CTSK, MMP2
Positive Regulation of Cyclin-Dependent Protein Serine/Threonine Kinase Activity	0.022884846	4.531148198	STIL, PSRC1, CKS2, CDC6, CKS1B
Regulation of Cell Proliferation	0.024179707	1.832420285	EGLN3, SPARC, TNC, CDCA7, PRG4, IGF1, BRCA1, CXCL3, CXCL13, FOXM1, BRCA2, CXCL5, NR3C2, CHEK1, SOX9, PF4, EZH2
Microtubule-Based Process	0.02486513	3.580712235	TUBA1C, TUBB6, TUBB2B, TUBB5, TUBB3, TUBE1
Epithelial Cilium Movement	0.025488237	6.117050068	NME5, AK7, KIF27, CCDC40
Inner Cell Mass Cell Proliferation	0.025488237	6.117050068	GINS1, CHEK1, NCAPG2, BRCA2
Negative Regulation of Smooth Muscle Cell Migration	0.025488237	6.117050068	NDRG4, IGFBP3, ADIPOQ, PPARGC1A
Sertoli Cell Development	0.025488237	6.117050068	NTRK1, WT1, SOX8, SOX9
Cell Proliferation In Forebrain	0.025488237	6.117050068	RRM1, FABP7, KIF14, DIXDC1
Axon Regeneration	0.025488237	6.117050068	DHFR, CHL1, APOE, FOLR1
Positive Regulation of Cellular Protein Metabolic Process	0.025488237	6.117050068	UHRF1, ADIPOQ, INHBA, AGT
Negative Regulation of Inflammatory Response	0.025570731	2.531193131	MIR147, TNFAIP6, SERPINF1, ADIPOQ, PBK, NR1H4, APOE, CD276, IL22RA2
Organ Regeneration	0.026050043	3.058525034	CCNA2, PTPRU, NNMT, CDK1, CCL2, MKI67, CXCL5
Mitotic Cell Cycle Checkpoint	0.029058918	4.218655219	ZWILCH, CHEK1, KNTC1, TTK, MAD2L1
Spindle Assembly	0.029058918	4.218655219	TPX2, TUBB5, RCC1, FBXO5, KIF11
Negative Regulation of Cytokinesis	0.030475355	10.48637154	E2F7, AURKB, E2F8
Positive Regulation of Male Gonad Development	0.030475355	10.48637154	WT1, SOX9, ZFPM2
Positive Regulation of Mitotic Cell Cycle Spindle Assembly Checkpoint	0.030475355	10.48637154	GEN1, NDC80, MAD2L1
Protein Localization to Chromatin	0.030475355	10.48637154	PLK1, ESCO2, EZH2
Positive Regulation of Ubiquitin Protein Ligase Activity	0.030475355	10.48637154	CDC20, UBE2S, PLK1
Copper Ion Import	0.030475355	10.48637154	ATP7B, STEAP4, STEAP1
Glomerular Visceral Epithelial Cell Differentiation	0.030475355	10.48637154	BASP1, WT1, KLF15
Lung Development	0.030531725	2.170566153	FOXA1, ADAMTS2, MMP14, SPARC, ARG1, WNT5A, IGF1, PTN, ZFPM2, NODAL, CCDC40
DNA Damage Checkpoint	0.032480729	4.078033378	CHEK2, H2AFX, CHEK1, E2F1, CLSPN
Synapsis	0.032480729	4.078033378	NDC1, BRIP1, CCNE2, FANCD2, CCNE1
Response to Glucocorticoid	0.033130294	2.609941362	ALDH3A1, PTPRU, SPARC, MDK, PAPPA, ADIPOQ, SCGB1A1, TYMS
Trophoblast Giant Cell Differentiation	0.034973286	5.437377838	PLK4, GJB5, E2F7, E2F8
Positive Regulation of Epithelial Cell Proliferation	0.035259736	2.575600028	OSR1, WNT5A, TWIST1, GAS1, IGF1, SOX9, NODAL, TBX18
Response to Nicotine	0.035533216	3.262426703	DHFR, NTRK1, EDN1, LYPD1, HMOX1, ABAT
Cellular Response to Ionizing Radiation	0.03612815	3.946483915	RAD51AP1, BLM, RAD51, FIGNL1, ECT2
One-Carbon Metabolic Process	0.03612815	3.946483915	DHFR, CAR3, CAR13, CAR6, ALDH1L2
Proteolysis	0.036506684	1.429413762	CFD, TPSB2, MYRF, MCPT4, CFI, ADAMTS12, MMP23, ADAMTS4, ADAMTS16, UCHL1, ADAM28, ADAMTS2, C1S1, CAPN6, ADAMTS19, CTSK, CLCA2, CPXM1, TPSAB1, ADAMTS7, CMA1, MMP2, ECEL1, PRSS35, MMP10, MMP14, MMP13, ESPL1, PAPPA, ADAM12, MMP19, PRTN3, CFB, PAPPA2

Positive Regulation of Cell Proliferation	0.037356911	1.44461699	SHC4, COL18A1, TNC, KIF14, PTN, FOXM1, CDC20, ESM1, HAS2, SOX9, TIMP1, FGFBP1, EDN1, WNT5A, CLEC11A, FN1, CDC7, IGF1, AGT, FABP1, ACER2, ALDH3A1, MLXIPL, CENPF, FABP4, PRC1, GAS1, PGR, CD248, KIF20B, FOLR2, NODAL
Cellular Response to UV	0.038578796	3.191504383	DDIAS, PBK, TRP73, PTN, BCAT1, AURKB
Positive Regulation of DNA-Directed DNA Polymerase Activity	0.039550273	9.175575101	RFC4, DSCC1, CHTF18
Attachment of Mitotic Spindle Microtubules to Kinetochore	0.039550273	9.175575101	CENPE, KIF2C, NDC80
Astrocyte Cell Migration	0.039550273	9.175575101	CCL12, MMP14, CCL2
Cerebellar Cortex Development	0.039550273	9.175575101	KIF14, DIXDC1, EZH2
Heart Development	0.039676611	1.687462088	EDN1, SPARC, DNAH5, MMP2, OSR1, DAW1, FOXJ1, PTN, CENPF, VCAN, COL3A1, MMP13, WT1, SOX9, ZFPM2, DRC1, NODAL, FBN1
Positive Regulation of Mitotic Cell Cycle	0.040002162	3.823156292	FOXA1, CCNB1, CDK1, BIRC5, BRCA2
Regulation of Blood Pressure	0.040286262	2.76253874	AOC3, CALCA, EDN1, COL1A2, C3AR1, HMOX1, AGT
Oocyte Maturation	0.040305423	5.151200057	CCNB1, FBXO5, TRIP13, BRCA2
Osteoblast Differentiation	0.041634336	2.165327458	COL1A1, VCAN, FIGNL1, IGFBP3, COL6A1, TNC, SPP1, TWIST1, SOX8, IGF1
Ossification	0.042407478	2.293893775	MMP14, SPARC, COL11A1, COL5A2, ALOX15, SPP1, TWIST1, SOX9, PTN
Regulation of Neuron Apoptotic Process	0.044103101	3.707303071	GABRB3, EGLN3, KIF14, TRP73, FAIM2
Positive Regulation of Smooth Muscle Cell Proliferation	0.044674869	2.446820027	EDN1, ORC1, C3AR1, HMOX1, IGF1, SKP2, PPARGC1A, WISP1
Positive Regulation of ERK1 and ERK2 Cascade	0.044840304	1.82210002	NTRK1, CCL24, NDRG4, CCL12, CCL11, ALOX15, TREM2, ADRA1A, CCL9, CCL8, CCL7, CCL2, FGF23, NODAL
DNA Metabolic Process	0.046022374	4.893640054	TOP2A, RAD51, TK1, MKI67
Retinal Ganglion Cell Axon Guidance	0.046022374	4.893640054	NRCAM, EPHB2, EFNA5, EPHB1
Stem Cell Differentiation	0.048430684	3.598264746	SHC4, OSR1, A2M, ETV4, PHF19
Oligodendrocyte Differentiation	0.048430684	3.598264746	CDKN2C, MYRF, SOX8, SOX9, BOK
Brown Fat Cell Differentiation	0.048430684	3.598264746	MRAP, FABP4, ADIPOQ, UCP1, EBF2
toxin Metabolic Process	0.049499272	8.156066757	CYP1A1, FMO1, CYP1B1
Regulation of Chromosome Segregation	0.049499272	8.156066757	KIF2C, MKI67, BUB1
Bone Trabecula Formation	0.049499272	8.156066757	FBN2, COL1A1, MMP2
Negative Regulation of Histone Acetylation	0.049499272	8.156066757	MSX3, TWIST1, BRCA1
Positive Regulation of Exit From Mitosis	0.049499272	8.156066757	UBE2C, CDCA5, BIRC5
Negative Regulation of Peptidase Activity	0.050054615	2.091299168	ITIH5, WFDC8, SERPINB2, CD109, BIRC5, BC048546, CST9, TIMP1, A2M, NAIP1
Protein Complex Assembly	0.052066615	2.59511215	CCNB1, GMNN, CDK1, SOX9, PF4, CLGN, ADD2
Response to X-Ray	0.052116053	4.660609575	BLM, RAD51, TRP73, BRCA2
Determination of Adult Lifespan	0.052116053	4.660609575	MEG3, ANKLE1, RAD54L, RAD54B
Placenta Development	0.052314903	2.936184032	GJB3, CCNF, E2F7, NODAL, DCN, E2F8
Negative Regulation of Blood Pressure	0.052984033	3.495457182	CALCA, PMCH, ADIPOQ, BDKRB1, ABAT
Regulation of Heart Rate	0.057761704	3.398361149	RYR2, CALCA, PMCH, AGT, CACNA1G
Positive Regulation of Leukocyte Migration	0.058577083	4.448763686	AOC3, CCL12, CCL2, BDKRB1
Cell-Cell Signaling	0.059674383	2.137998082	FGFBP1, EDN1, WNT5A, CXCL13, CCR5, ADRA1A, WISP1, ADRA2A, WNT4
Heterophilic Cell-Cell Adhesion via Plasma Membrane Cell Adhesion Molecules	0.060122722	2.823253877	LGALS1, CADM4, TENM3, CDH2, GM609, LGALS7
Nucleotide Biosynthetic Process	0.06023609	7.340460081	DHFR, DCTD, TYMS
Cellular Response to Vitamin D	0.06023609	7.340460081	TNC, PTN, FGF23
Nucleosome Assembly	0.062454544	2.117440408	CHAF1B, CHAF1A, H1FX, PADI4, SOX9, CENPA, HIST1H1B, MCM2, ASF1B
Positive Regulation of Bone Mineralization	0.062761717	3.30651355	FBN2, OSR1, FAM20C, CD276, WNT4
Base-Excision Repair	0.062761717	3.30651355	POLQ, NEIL3, FEN1, LIG1, UNG
Positive Regulation of Mesenchymal Cell Proliferation	0.062761717	3.30651355	WNT5A, PRRX2, GAS1, SOX9, TBX18
Positive Regulation of Cardiac Muscle Cell Proliferation	0.065394973	4.255339178	CCNB1, CDK1, NCAM1, ZFPM2
Cochlea Morphogenesis	0.065394973	4.255339178	WNT5A, SOX9, TBX18, CTHRC1
Negative Regulation of Smooth Muscle Cell Proliferation	0.067981582	3.219500036	NDRG4, IGFBP3, ADIPOQ, HMOX1, PPARGC1A
Protein Kinase B Signaling	0.067981582	3.219500036	LINGO1, CCL12, CCL2, IGF1, SOX9
Intrinsic Apoptotic Signaling Pathway In Response to DNA Damage	0.068556158	2.718688919	CHEK2, E2F1, HMOX1, BRCA1, BRCA2, BOK
Cellular Response to Glucose Stimulus	0.069459154	2.412357773	MLXIPL, LGALS1, CMA1, SERPINF1, AQP4, IGF1, PPARGC1A
Regulation of Cellular Protein Localization	0.071680036	6.673145528	CCNE2, CCNE1, WNT5A
Negative Regulation of Immune Response	0.071680036	6.673145528	FCRLB, COL3A1, FCGR2B
Positive Regulation of Insulin-Like Growth Factor Receptor Signaling Pathway	0.071680036	6.673145528	CDH3, IGFBP3, IGF1
Skin Morphogenesis	0.071680036	6.673145528	COL1A1, ERRFI1, COL1A2

Hepatocyte Differentiation	0.071680036	6.673145528	CYP1A1, E2F7, E2F8
Telomere Maintenance via Recombination	0.071680036	6.673145528	RAD51, RAD51C, BRCA2
Negative Regulation of Tumor Necrosis Factor-Mediated Signaling Pathway	0.071680036	6.673145528	ADIPOQ, NR1H4, TRAIP
Cellular Response to Gamma Radiation	0.07255827	4.078033378	RAD51, DDIAS, CHEK2, H2AFX
Middle Ear Morphogenesis	0.07255827	4.078033378	EDN1, OSR1, PRRX2, GAS1
Anatomical Structure Formation Involved In Morphogenesis	0.07255827	4.078033378	FOXA1, LHX2, NODAL, TBX18
Complement Activation, Classical Pathway	0.07300579	2.669258211	C1QB, C1QA, C1S1, CFI, HC, C1QC
Cell Fate Commitment	0.073295304	2.378852804	WNT5A, GAS1, SOX8, ROR2, SOX9, NODAL, WNT4
Embryonic Cranial Skeleton Morphogenesis	0.073418328	3.136948753	MMP14, PRRX2, TWIST1, GAS1, NODAL
Regulation of Signal Transduction	0.079068535	3.058525034	CDH2, 2810408A11RIK, BIRC5, NAIP1, NODAL
Positive Regulation of DNA Endoreduplication	0.079964515	24.46820027	E2F7, E2F8
Actomyosin Contractile Ring Assembly	0.079964515	24.46820027	RACGAP1, KIF23
Negative Regulation of Endodermal Cell Differentiation	0.079964515	24.46820027	COL5A1, COL5A2
Ureter Urothelium Development	0.079964515	24.46820027	OSR1, SOX9
Sequestering of TGFbeta In Extracellular Matrix	0.079964515	24.46820027	FBN2, FBN1
Minus-End-Directed Vesicle Transport Along Microtubule	0.079964515	24.46820027	KIFC1, KIFC5B
Spindle Assembly Involved In Female Meiosis I	0.079964515	24.46820027	FBXO5, AURKA
Centrosome Separation	0.079964515	24.46820027	NEK2, CNTROB
Dump Biosynthetic Process	0.079964515	24.46820027	DCTD, DUT
Mitotic Recombination-Dependent Replication Fork Processing	0.079964515	24.46820027	RAD51, BRCA2
Posterior Mesonephric Tubule Development	0.079964515	24.46820027	WT1, OSR1
Establishment of Mitotic Spindle Orientation	0.080054708	3.914912043	INSC, SPDL1, CENPA, NDC80
Positive Regulation of Cytosolic Calcium Ion Concentration	0.081559448	1.818582453	EDN1, PTGER3, PMCH, C3AR1, CXCL1, CXCL13, CCR5, CXCL3, ADRA1A, AGT, CACNA1G
Cellular Response to Hydrogen Peroxide	0.082365321	2.575600028	FABP1, ARG1, CDK1, CYP1B1, ECT2, EZH2
Inner Dynein Arm Assembly	0.083755675	6.117050068	LRRC6, ZMYND10, CCDC40
Mesonephros Development	0.083755675	6.117050068	WT1, OSR1, WNT4
Positive Regulation of Cell Growth Involved In Cardiac Muscle Cell Development	0.083755675	6.117050068	EDN1, IGF1, WISP1
Lung Epithelial Cell Differentiation	0.083755675	6.117050068	FOXA1, INSC, SOX9
Nucleoside Triphosphate Biosynthetic Process	0.083755675	6.117050068	NME4, AK5, AK7
Regulation of G2/M Transition of Mitotic Cell Cycle	0.083755675	6.117050068	CCNA2, CENPF, KIF14
Central Nervous System Projection Neuron Axonogenesis	0.083755675	6.117050068	EPHB2, EPHB1, DCLK1
Skin Development	0.087272288	2.531193131	COL1A1, ADAMTS2, COL3A1, COL5A1, GJB3, COL5A2
Mitophagy In Response to Mitochondrial Depolarization	0.091174008	1.853651536	FABP1, FGFBP1, CALCA, CHAF1B, CALCB, SNTG1, KRT15, HC, HAPLN1, ADAMTS7
Meiotic Cell Cycle	0.093397043	2.060480023	CCNA1, RSPH1, EXO1, H2AFX, NEK2, TRIP13, CLGN, AURKA
Nervous System Development	0.093627566	1.427852536	NTRK1, SRRM4, NRN1, D130043K22RIK, CRMP1, GFRA1, IGF1, EFNA5, DCLK1, CIT, CDC20, DCDC2A, LHX2, CHL1, DPF1, INSC, STMN1, ECT2, EPHB2, EPHB1, DBN1, NODAL
Response to Hypoxia	0.094858812	1.65670106	RYR2, EDN1, EGLN3, MMP2, ADIPOQ, ABAT, LOXL2, ALDH3A1, MMP14, CYP1A1, TACC3, HMOX1, CCL2
Odontogenesis	0.09599465	3.624918559	OSR1, TWIST1, GAS1, INHBA
Fatty Acid Homeostasis	0.096392539	5.646507755	MLXIPL, NR1H4, APOE
Hyaluronan Metabolic Process	0.096392539	5.646507755	ITIH5, HAS2, MKI67
Short-Term Memory	0.096392539	5.646507755	BRINP1, MDK, SERPINF1
Somatic Hypermutation of Immunoglobulin Genes	0.096392539	5.646507755	POLQ, EXO1, UNG
Negative Regulation of JAK-STAT Cascade	0.097259589	2.845139566	LRRC15, FLRT2, NYX, ASPN, DCN
Peptidyl-Threonine Phosphorylation	0.097259589	2.845139566	OSR1, CHEK1, PBK, CDK1, TTK
Microtubule Cytoskeleton Organization	0.097407459	2.039016689	WEE1, GAS2L3, PRC1, TACC3, NUSAP1, CRMP1, BIRC5, AURKA
