# Supplementary material for: Inhibition of angiogenesis and regenerative lung growth in Lepob/ob mice through adiponectin-VEGF/VEGFR2 signaling
Source: Front Cardiovasc Med. 2024 Oct 16;11:1491971. doi: 10.3389/fcvm.2024.1491971 (PMC11521822; doi:10.3389/fcvm.2024.1491971)
Supplement: Supplementary file 6 [file Datasheet4.pdf]

Biological Processes	PValue	Fold Enrichmer	Genes
Cell Cycle	2.5868E-52	4.98131113	ERCC6L, ZWILCH, DSCC1, GMNN, CCNF, DIXDC1, BUB1B, MKI67, SMC2, CDC20, CHEK2, CHEK1, NUSAP1, RCC1, NEK2, OIP5, KNTC1, FBXO5, BORA, LIG1, TRP73, ESCO2, CDC25C, KNSTRN, MELK, CCNE2, CCNE1, FANCD2, FAM64A, KIF20B, CDCA2, CDCA3, CDCA5, CDCA8, PKMYT1, SKA3, PMF1, NCAPH, SKA1, SKA2, CCNB2, DSN1, CCNB1, BRINP1, RACGAP1, CLSPN, ECT2, FAM83D, CDT1, UBE2C, DDIAS, PLK1, CDC7, CDC6, NDC80, ANLN, TPX2, KIF18B, UBE2S, CDK1, CNTROB, MCM7, MCM8, NCAPG2, BRCA1, KIF11, CHTF18, FOXM1, BRCA2, CKS1B, SMPD3, CHAF1B, CHAF1A, SGOL1, MIS18BP1, GSG2, NUF2, MYBL2, SPDL1, DLGAP5, CEP55, HELLS, H2AFX, CKAP2, KIF23, MASTL, CCSAP, CIT, CCNA2, ASPM, CCNA1, DBF4, PSRC1, INCENP, MCM3, CKS2, BIRC5, GAS1, MCM5, KIF2C, MCM6, MCM2, UHRF1, NSL1, AURKB, AURKA, CDC45, E2F1, BUB1, E2F7, E2F8, CENPW, CDKN2C, SPAG5, TICRR, CENPE, WEE1, APITD1, PRC1, NCAPD2, SPC24, MAD2L1, SPC25, NEK11, CDKN3
DNA Replication	2.0729E-20	7.161424469	PIF1, FEN1, BLM, 2810417H13RIK, MCM7, MCM8, PRIM1, DSCC1, MCM10, **BRCA1**, CHTF18, CHAF1B, CDC45, CHAF1A, ORC1, POLE, GINS1, POLQ, GINS2, CDT1, RMI2, RRM1, RRM2, RFC4, LIG1, CDC6, ADRA2A, TICRR, POLA1, DBF4, POLE2, MCM3, MCM5, MCM6, DTL, MCM2
Chromosome Segregation	7.0773E-18	7.972784358	TOP2A, CDCA2, **BRCA1**, KIF11, SKA3, PMF1, NSL1, SKA1, SKA2, DSN1, SGOL1, RCC1, NEK2, OIP5, BUB1, CENPW, SPAG5, ESCO2, KNSTRN, NDC80, CENPE, CENPF, ESPL1, CENPH, INCENP, BIRC5, KIF2C, CENPN, SPC25
Cellular Response to DNA Damage Stimulus	1.1637E-10	2.854623365	TOP2A, FEN1, MCM7, MCM8, MCM10, BRCA1, FOXM1, BRCA2, PIDD1, CHAF1B, CHAF1A, EXO1, CHEK2, CHEK1, POLE, GEN1, LIG1, H2AFX, TRP73, MASTL, MMS22L, NEIL3, RAD51C, FANCD2, SPATA18, DTL, PIF1, 2810417H13RIK, BLM, PARPBP, UHRF1, UNG, RAD54B, RAD51AP1, BRIP1, RAD54L, CLSPN, E2F7, ZRANB3, BARD1, POLQ, EGLN3, ATAD5, DDIAS, TICRR, RAD51, APITD1, UBE2T, CDKN3
DNA Repair	3.2633E-08	2.846928962	PIF1, FEN1, BLM, 2810417H13RIK, ANKLE1, PARPBP, MCM8, UHRF1, BRCA1, FOXM1, BRCA2, UNG, RAD51AP1, BRIP1, CHAF1B, CHAF1A, EXO1, CHEK2, CHEK1, RAD54L, CLSPN, POLE, ZRANB3, GEN1, BARD1, POLQ, LIG1, H2AFX, KIF22, TICRR, MMS22L, NEIL3, RAD51C, RAD51, APITD1, FANCD2, UBE2T
Chemokine-Mediated Signaling Pathway	2.3318E-07	6.22826916	CCL24, CCL12, CCL11, CXCL1, CXCL3, CXCL13, CXCL5, CCL9, CCL8, CCL7, CCR9, **CCL2**, ACKR1, **PF4**
Cellular Response to Tumor Necrosis Factor	4.7248E-07	4.226325501	CCL24, CCL12, EDN1, CALCA, CCL11, **BRCA1**, ADAMTS12, COL1A1, CCL9, CCL8, FABP4, CCL7, OCSTAMP, **CCL2**, HAS2, PCK1, PPARGC1A, DBN1, ADAMTS7
Cellular Response to Interleukin-1	3.8958E-06	4.587787551	CCL24, CCL12, EDN1, CCL11, SAA3, FN1, ADAMTS12, CCL9, CCL8, CCL7, **CCL2**, HAS2, **SOX9**, PCK1, ADAMTS7
Double-Strand Break Repair via Homologous Recombination	1.74E-05	4.677744169	GEN1, BLM, MCM8, H2AFX, TONSL, **BRCA1**, **BRCA2**, RAD54B, RAD51AP1, MMS22L, RAD51, RAD51C, RAD54L
Chemotaxis	2.4136E-05	3.5250797	CCL24, CCL12, CCL11, HMGB2, CXCR5, CXCL3, CXCL13, CXCL5, CCL9, CCL8, CCL7, CCR9, C3AR1, **CCL2**, CCR5, CCR3, **PF4**
Collagen Catabolic Process	3.4529E-05	8.156066757	ADAMTS2, MMP14, MMP13, CTSK, **MMP2**, MMP19, PRTN3, MMP10
Aging	8.6264E-05	2.828693673	NTRK1, CALCA, ARG1, SERPINF1, ABAT, TYMS, ADRA1A, AURKB, AGT, DCN, ALDH3A1, COL3A1, KRT14, CYP1A1, **CCL2**, NCAM1, TIMP1, **APOE**, PCK1, PPARGC1A
Cytokinesis	9.9936E-05	5.95172439	PRC1, INCENP, PLK1, BIRC5, KIF23, KIF20A, ECT2, **BRCA2**, CIT
Cell Activation	0.00016445	10.48637154	LYPD1, FN1, LYPD2, **IGF1**, TIMP1, SLURP1
Inflammatory Response	0.00019011	2.133854675	CCL12, TPSB2, CALCA, CCL11, CD5L, HMGB2, CXCL1, REG3G, CXCL3, CXCL13, CXCL5, CCL9, CYP26B1, CCL8, CCL7, SPP1, C3AR1, **CCL2**, BDKRB1, CCR5, CCR3, CCL24, CD163, NR1H4, TRP73, CHIL3, HC, ACKR1, CHIL4, **PF4**
Cellular Response to Retinoic Acid	0.00028292	4.140772353	COL1A1, CYP26A1, CYP26B1, BRINP1, TESC, SERPINF1, OSR1, TNC, **CCL2**, **SOX9**, PCK1
Response to Drug	0.00034446	2.093149876	COL18A1, NNMT, MCM7, HMGB2, ABAT, PTN, TYMS, ADRA1A, RAD54B, CCNB1, CDH3, LGALS1, MDK, RAD54L, NCAM1, PPARGC1A, ABCB1B, NTRK1, CBX7, ARG1, **MMP2**, TRP73, INHBA, COL1A1, ALDH3A1, RAD51, SCGB1A1, CYP1A1, CDK1
Cellular Response to Interferon-Gamma	0.0004117	3.95809122	CCL24, CCL9, CCL12, EDN1, CCL8, CCL11, GBP10, CCL7, WNT5A, **CCL2**, AQP4
Eosinophil Chemotaxis	0.00047459	12.23410014	CCL24, CCL11, CCL7, **CCL2**, CCR3
Endodermal Cell Differentiation	0.00064253	6.343607478	MMP14, COL11A1, **MMP2**, COL6A1, FN1, INHBA, NODAL
Male Gonad Development	0.00064527	3.058525034	RRM1, TESC, WNT5A, HMGB2, LRRC6, INHBA, ASPM, MMP14, BRIP1, **WT1**, SOX8, **SOX9**, WNT4, BOK
DNA Recombination	0.0006617	3.454334156	RAD51AP1, PIF1, BLM, RAD51, RAD51C, LIG1, EXO1, H2AFX, HMGB2, **BRCA1**, **BRCA2**, RAG1
Spermatogenesis	0.0006725	1.923789702	LRGUK, HMGB2, **BRCA2**, CHTF18, AK7, C330027C09RIK, CLGN, BRIP1, ADAMTS2, CCNB1, CYP26B1, RACGAP1, **E2F1**, 1110017D15RIK, SOX8, **SOX9**, TSNAXIP1, CCDC33, TCP11, RPL39L, NDC1, H2AFX, NME5, CDC25C, CIT, CCNA1, ASPM, RAD51C, **WT1**, MYCBPAP, TRIP13, SPATA18
Cell Proliferation	0.00071284	2.335600935	MCM7, UHRF1, MCM10, **IGF1**, MKI67, **BRCA2**, AURKB, CKS1B, POLA1, UCHL1, MELK, CKS2, CYP1A1, CDK1, TACC3, FAM83D, BUB1, TSPAN1, CFB, E2F8, BOK
Extracellular Matrix Organization	0.00076401	3.0048667	VIT, COL18A1, OLFML2B, LAMA1, ELN, FN1, NID2, AGT, CCDC80, COL5A3, ADAMTSL2, COL6A4, VWA1, **SOX9**
Protein Phosphorylation	0.00076859	1.741660089	LAMA1, BUB1B, TTK, MST1R, PKMYT1, AURKB, AURKA, CCNB1, STK33, CHEK2, GSG2, CHEK1, PBK, NEK2, EPHB2, BUB1, EPHB1, PLK4, NTRK1, CDKL4, NEK5, IGFBP3, PLK1, WNT5A, CDC7, PASK, MASTL, MAPK15, DCLK1, CIT, MAPK10, WEE1, MELK, CCNE1, MAP3K19, CDK1, BIRC5, FAM20C, ROR2, CAMK1G, NEK11
Positive Regulation of Inflammatory Response	0.00098299	3.883841313	CCL24, CCL9, CCL12, CCL8, CCL11, FABP4, CCL7, WNT5A, **CCL2**, CCR5
Monocyte Chemotaxis	0.00105388	4.893640054	CCL24, CCL9, CCL12, CALCA, CCL8, CCL11, CCL7, **CCL2**
G2 DNA Damage Checkpoint	0.00131787	6.990914363	PLK1, CHEK1, CLSPN, **BRCA1**, DTL, NEK11
Neutrophil Chemotaxis	0.00189506	3.546115981	CCL24, CCL9, CCL12, CCL8, CCL11, CCL7, SPP1, **CCL2**, CXCL1, CXCL3
Lymphocyte Chemotaxis	0.00195245	5.1902243	CCL24, CCL9, CCL12, CCL8, CCL11, CCL7, **CCL2**
Cellular Response to Platelet-Derived Growth Factor Stimulus	0.00203681	6.383008766	CCNA2, ERRFI1, HAS1, **CCL2**, HAS2, PTN
Positive Regulation of Transcription, DNA-Templated	0.00254927	1.65670106	MEG3, BLM, MYRF, HMGB2, **BRCA1**, FOXM1, **BRCA2**, CKS1B, MDK, CHEK2, **E2F1**, SOX8, **SOX9**, PPARGC1A, **FGF23**, WNT4, TESC, ATAD2, WNT5A, TRP73, EBF2, EBF3, **IGF1**, INHBA, GDF6, KLF15, ETV4, AGT, CCNA2, COL1A1, MLXIPL, PSRC1, **WT1**, LHX2, CKS2, NFE2L3, PGR, ROR2, ZFPM2
Response to Lipopolysaccharide	0.00300541	2.235673121	SPON2, EDN1, WFDC21, SPARC, PTGER3, HMGB2, FMO1, CXCL1, CXCL3, CXCL13, LOXL1, CXCL5, DCN, SCGB1A1, CYP1A1, BDKRB1, PCK1, **PF4**
Cellular Response to Hypoxia	0.00319676	2.850664109	FABP1, CCNA2, CCNB1, EDN1, FNDC1, **E2F1**, TWIST1, HMOX1, PTN, PCK1, PPARGC1A, KCNK2
Maternal Process Involved In Parturition	0.0032506	12.23410014	EDN1, CYP1A1, **CCL2**, NODAL
Response to Activity	0.00353717	3.994808207	NTRK1, EDN1, ADIPOQ, CDK1, **CCL2**, PTN, PCK1, PPARGC1A
ERK1 and ERK2 Cascade	0.00359888	5.646507755	CCL11, **IGF1**, **SOX9**, IQGAP3, CCR3, AGT
Response to Mechanical Stimulus	0.00395037	3.495457182	COL3A1, MMP14, CCNB1, **MMP2**, TNC, **CCL2**, BDKRB1, KCNK2, DCN
Double-Strand Break Repair	0.00395037	3.495457182	POLQ, LIG1, CHEK2, CDCA5, RAD54L, TRIP13, ESCO2, **BRCA1**, **BRCA2**
Immune Response	0.00400049	1.97904561	H2-EB2, CCL24, CCL12, CCL11, CMA1, MCPT4, CXCR5, CXCL1, PRG4, CXCL3, CXCL13, CXCL5, VPREB3, CCL9, CCL8, CCL7, CCR9, **CCL2**, ENPP3, CCR5, FCGR2B, **PF4**
Positive Regulation of Cell Migration	0.00408404	2.169594113	COL18A1, LRRC15, CCL24, EDN1, CEMIP, CCL11, TNFAIP6, **MMP2**, ARHGEF39, FN1, **IGF1**, ADRA2A, COL1A1, MMP14, FAM110C, C3AR1, HAS2, ROR2
Cell Chemotaxis	0.00441092	3.136948753	CCL9, CCL12, CCL8, SAA3, HMGB2, **CCL2**, CXCL1, CXCL13, EPHB1, CXCL5
Protein K6-Linked Ubiquitination	0.00472878	10.87475568	BARD1, UBE2S, UBE2T, **BRCA1**
Retinal Rod Cell Differentiation	0.00485542	24.46820027	SOX8, **SOX9**, PTN
Renal Vesicle Induction	0.00485542	24.46820027	SOX8, **SOX9**, WNT4
Negative Regulation of Neuron Apoptotic Process	0.00596801	2.293893775	GABRB3, NTRK1, CCL12, KIF14, TRP73, FAIM2, AGT, CHL1, MDK, HMOX1, **CCL2**, BIRC5, **APOE**, PPARGC1A, BOK
Regulation of Cell Cycle	0.0060571	2.621592886	FOXA1, 2810417H13RIK, CCNE2, CCNE1, FIGNL1, CCNF, **E2F1**, TACC3, MASTL, SKP2, DTL, PRR11
Complement Activation, Alternative Pathway	0.006552	9.787280108	CFD, HC, **CFP**, CFB
Positive Regulation of Gtpase Activity	0.00684686	2.346265779	NTRK1, CCL24, CCL12, CCL11, RGS16, WNT5A, ELMOD1, CCL9, CCL8, CCL7, **CCL2**, ECT2, EZH2, WNT4

Categories
Adhesion/Migration
Cell Cycle/Apoptosis
Inflammatory/Immune Response
Cell Signaling
